# Protected area connectivity: Shortfalls in global targets and country-level priorities

**DOI:** 10.1016/j.biocon.2017.12.020

**Published:** 2018-03

**Authors:** Santiago Saura, Bastian Bertzky, Lucy Bastin, Luca Battistella, Andrea Mandrici, Grégoire Dubois

**Affiliations:** European Commission, Joint Research Centre (JRC), Directorate D - Sustainable Resources, Via E. Fermi 2749, I-21027 Ispra, VA, Italy

**Keywords:** Protected areas, Connectivity indicators, Aichi Targets, Ecological networks

## Abstract

Connectivity of protected areas (PAs) is crucial for meeting their conservation goals. We provide the first global evaluation of countries' progress towards Aichi Target 11 of the Convention on Biological Diversity that is to have at least 17% of the land covered by well-connected PA systems by 2020. We quantify how well the terrestrial PA systems of countries are designed to promote connectivity, using the Protected Connected (ProtConn) indicator. We refine ProtConn to focus on the part of PA connectivity that is in the power of a country to influence, i.e. not penalizing countries for PA isolation due to the sea and to foreign lands. We found that globally only 7.5% of the area of the countries is covered by protected connected lands, which is about half of the global PA coverage of 14.7%, and that only 30% of the countries currently meet the Aichi Target 11 connectivity element. These findings suggest the need for considerable efforts to improve PA connectivity globally. We further identify the main priorities for improving or sustaining PA connectivity in each country: general increase of PA coverage, targeted designation of PAs in strategic locations for connectivity, ensuring permeability of the unprotected landscapes between PAs, coordinated management of neighbouring PAs within the country, and/or transnational coordination with PAs in other countries. Our assessment provides a key contribution to evaluate progress towards global PA connectivity targets and to highlight important strengths and weaknesses of the design of PA systems for connectivity in the world's countries and regions.

## Highlights

• We evaluate countries' efforts in designing well-connected terrestrial protected area (PA) systems.
• We distinguish the PA isolation caused by limitations in a country's PA system, by the sea and by foreign lands.
• Of the global land area, only 7.5% is protected and connected, which is about half of the 14.7% under protection.
• Only a third of countries meet Aichi Target 11 of having 17% of land covered by well-connected PAs.
• We identify the main priorities for improving or sustaining PA connectivity in each country.

## Graphical abstract

**Figure:**
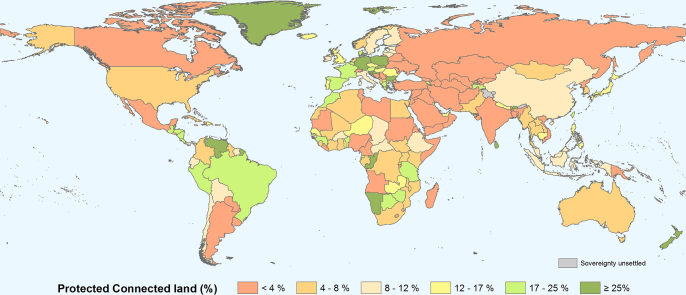

## 1 Introduction

Protected areas (PAs) are critical for biodiversity conservation. Well designed and managed PA systems can effectively safeguard species and ecosystems, and deliver essential ecosystem services to people (Rands et al., 2010, Watson et al., 2014, UNEP-WCMC and IUCN, 2016). Connectivity of PA systems is necessary to facilitate large-scale ecological and evolutionary processes such as gene flow, migration and species range shifts. These processes are all essential for the persistence of viable populations, especially when facing climatic and environmental changes in increasingly transformed and fragmented landscapes (Kuussaari et al., 2009, Krosby et al., 2010, Beale et al., 2013). Improving or sustaining PA connectivity is therefore a primary concern for the effective conservation and management of biodiversity (Ervin et al., 2010, Laurance et al., 2012, Juffe-Bignoli et al., 2014).

The importance of PA connectivity is also recognized in global biodiversity targets adopted by the world's governments. In 2010, the parties to the United Nations Convention on Biological Diversity (CBD) adopted a Strategic Plan for Biodiversity for the 2011–2020 period, including the twenty Aichi Biodiversity Targets (CBD, 2010). In Aichi Target 11 the international community agreed to increase by 2020 the terrestrial area under protection to at least 17% in ‘effectively and equitably managed, ecologically representative and well-connected systems of protected areas’ (CBD, 2010). However, the CBD has neither provided a clear definition of the term ‘well-connected’, nor guidance on how to measure PA connectivity (Bertzky et al., 2012, Butchart et al., 2016), which has made it difficult to stimulate and track progress towards the Aichi Target 11 connectivity element. Recently, the first global assessments on PA connectivity have been reported (Santini et al., 2016, Saura et al., 2017). In particular, Saura et al. (2017) proposed the Protected Connected indicator (ProtConn), which considers different categories of land (unprotected, protected or transboundary) through which movement between protected locations may occur. ProtConn can be compared with PA coverage and used directly to quantify shortfalls, or successes, in achieving the connectivity element of Aichi Target 11. Saura et al. (2017) used ProtConn to examine the connectivity of PA systems for all terrestrial ecoregions, but a country-level evaluation can provide a more policy-relevant assessment since most political decisions on development and management of PA networks are taken at the national level. In addition, the analyses in Saura et al. (2017) did not distinguish between different causes of PA isolation but amalgamated them in the obtained connectivity scores. When extending the method to a country-level analysis, it is important to recognise that some of these causes will be outside the control or jurisdiction of a country; for example, PAs that are naturally separated by the sea or that are separated by the lands of other nations (e.g. Alaska and the rest of the USA).

Here we provide the first global evaluation of countries' progress towards the Aichi Target 11 element on well-connected PA systems. We quantify the percentage of each country that is covered by lands that are both protected and connected, using a refined version – called ProtConn_Bound_ – of the ProtConn indicator. ProtConn_Bound_ is obtained by separating three different potential sources of PA isolation: limitations of the design of the PA system in a country, natural isolation of PAs by the sea, and isolation due to intervening land belonging to other countries. Through this distinction, ProtConn_Bound_ focuses on the part of PA connectivity that is within the power of a country to influence, allowing for a fair comparison which does not systematically penalize countries with multiple islands or landmasses. In our assessment, we consider a range of median dispersal distances (1–100 km) encompassing the dispersal abilities of the large majority of terrestrial vertebrates, and we do not account for heterogeneity of the unprotected land between PAs. Our focus is on the structure and spatial arrangement of the network of PAs, and on the number, size and coverage of PAs in a country, rather than on the particularities of the landscape matrix and of the variable species-specific responses to it. Our aim is to assess how well the PA systems themselves are designed to support or promote connectivity, and how self-sufficient they are to do so in the long term.

We further interpret the detailed results of the indicator to identify, for each country, the main strategic priorities to improve or sustain PA connectivity. Our classification shows, from a connectivity perspective, whether a country should prioritize: general increase of PA coverage; targeted designation of PAs in strategic locations for connectivity; ensuring permeability of the unprotected landscapes in between PAs; coordinated management of neighbouring PAs within the country; or transnational coordination with PAs in other countries. This classification approach highlights important strengths and weaknesses of the design of PA systems for connectivity in the world's countries and regions, and is illustrated in more detail for selected examples.

Our final goal is to provide a country-level indicator of PA connectivity which can be directly used by the CBD, its Parties and the CBD-mandated Biodiversity Indicators Partnership (BIP^1^), to promote and assess progress towards Aichi Target 11 and future PA connectivity targets.

## 2 Methods

### 2.1 Protected areas

We downloaded the public version of the World Database on Protected Areas (WDPA) for June 2016 from Protected Planet.^2^ The WDPA is managed by the World Conservation Monitoring Centre (WCMC) of the United Nations Environment Programme (UNEP) in collaboration with the International Union for Conservation of Nature (IUCN), and is collated from national and regional datasets (IUCN and UNEP-WCMC, 2016). In June 2016, the WDPA contained around 200,000 terrestrial PAs. As in other recent global PA assessments (e.g. UNEP-WCMC and IUCN, 2016), we (i) excluded PAs with a “proposed” or “not reported” status, sites reported as points without an associated reported area, and UNESCO Man and the Biosphere Reserves, (ii) considered all PA types, including PAs for which the WDPA does not indicate an IUCN management category (“not reported” or “not assigned”), and (iii) included those PAs provided in the WDPA as points with unknown boundaries but with a reported area (about 6% of all PAs), using a geodesic circular buffer with an area equal to the reported value. The PA polygons (including the buffered points) were dissolved to remove all overlaps between different designation types and avoid double counting (e.g. where the same area is designated both as a National Park and as a World Heritage Site). The PA polygons in the dissolved layer could hence correspond to several overlapping or adjacent PAs. In order to facilitate computation of the inter-PA distance calculations on the dissolved vector layer (see Section 2.3) we reduced the number of vertices in the polygons using the Simplify Geometries tool in QGIS 2.12 with a tolerance of 100 m.

### 2.2 Land and countries

To exclude marine PAs and the marine portion of coastal PAs from our analysis, we clipped the above-described PA polygon layer with the land mask obtained from the Global Administrative Unit Layers (GAUL) for year 2015, developed by the Food and Agricultural Organization (FAO) of the United Nations.^3^ The resultant polygons were converted to single parts, yielding a set of individual terrestrial PA polygons, hereafter referred to simply as PAs for brevity. We calculated the area of each PA using the Mollweide projection. For computational feasibility of the connectivity calculations, we removed those PA polygons with an area smaller than 1 km^2^. This size threshold is consistent with other previous analysis on PAs at global or European scales (Leroux et al., 2010, Opermanis et al., 2012, Santini et al., 2016, Saura et al., 2017) and retained 99.8% of the total land area covered by PAs globally.

PAs were assigned to each country based on the ISO3 country code reported in the WDPA. For 17 PAs, most of them World Heritage Sites, multiple ISO3 codes were reported in the WDPA; these PAs were intersected with the GAUL country boundaries to distribute their area between the countries. Consistent with other global assessments (UNEP-WCMC and IUCN, 2016) and information systems on PAs,^4^ we here refer to the ISO3 coded geographical entities as “countries”, and report our results at this country level. It should be noted, however, that in some cases these ISO3 codes correspond to territories under the sovereignty of other nations, even if they are usually separately considered in this type of assessments. Some examples are Réunion Island, a French overseas territory located in the Indian Ocean, or Greenland, a self-governing territory that is part of the Kingdom of Denmark. In our analyses, we obtained, after excluding Antarctica, a total of 233 countries (ISO3 codes) that had some area under protection according to the PA size threshold used in this study, i.e. with at least one PA polygon with an area exceeding or equal to 1 km^2^. The area of each country, which is used in the calculation of the indicators described below, was calculated from GAUL using the Mollweide projection, excluding any disputed territories (sovereignty unsettled) as mapped in GAUL. The use of the ISO3 codes in the WDPA and of the GAUL definitions of country boundaries and disputed territories does not imply any endorsement by the authors, nor any official position by the European Commission, on the sovereignty of these lands.

### 2.3 Dispersal kernels, inter-PA distances and transboundary PAs

To assess the connectivity of PA systems, we considered four median dispersal distances (*d*_*med*_) of 1, 10, 30 and 100 km. This 1–100 km range covers the median dispersal abilities of the majority of terrestrial species (Saura et al., 2017), and matches the one used in a previous PA connectivity analysis for the USA (Minor and Lookingbill, 2010). Since 10 km is the central value of the log-transformed range of dispersal distances considered (1–100 km), we selected 10 km as the reference *d*_*med*_ for which we preferentially show the results of the connectivity analysis, but results for all other *d*_*med*_ values are also available.

The probability of direct dispersal (*p*_*ij*_) between two PAs *i* and *j* was calculated through a negative exponential function of the distance separating the edges of the PAs, in which *p*_*ij*_ = 0.5 for those PAs separated by a distance equal to the species median dispersal distance (*d*_*med*_), as shown in Fig. A1 in Appendix A. The negative exponential dispersal kernel gives a continuous variation in the strength of a connection in response to inter-PA distance (Fig. A1), and is widely used in connectivity or metapopulation analyses (e.g. Hanski and Ovaskainen, 2000, Saura and Pascual-Hortal, 2007, Visconti and Elkin, 2009, Gurrutxaga et al., 2011, Maiorano et al., 2015, Santini et al., 2016, Engelhard et al., 2017). Note that, according to this kernel, there is some likelihood of dispersal (0.5 > *p*_*ij*_ > 0) for inter-PA distances larger than the median *d*_*med*_ (Fig. A1). See Appendix A1 for further details on this dispersal kernel.

For each country, we converted the PA layer to an azimuthal equidistant projection centered on the centroid of all the country's PAs. Using this projection, we buffered the country's PAs by a distance of 500 km. All PAs not belonging to the country that fell (entirely or partially) within this buffer were considered as transboundary areas which could potentially contribute to connectivity between the PAs of the country under consideration. In this way, we accounted for the possibility that the connectivity of two PAs within a country could be enhanced by a PA located outside the country that functioned as a corridor or stepping stone between them. This transboundary contribution is considered in the equation for the ProtConn indicator presented in Section 2.4 below. A buffer of 500 km was used because this distance is much larger than the maximum considered median dispersal distance (*d*_*med*_ = 100 km), and therefore includes all pairs of PAs between which dispersal movements may be likely. Even for *d*_*med*_ = 100 km, the probability of a dispersal movement between PAs separated by > 500 km is only 0.03 (see Appendix A1).

Using the country-specific azimuthal equidistant projection, we calculated the distances between the edges (boundaries) of all the PAs (including both the country PAs and the transboundary PAs), which were then converted into direct dispersal probabilities (*p*_*ij*_) using the above-mentioned negative exponential dispersal kernel.

### 2.4 Protected Connected (ProtConn) and its fractions

The ProtConn indicator of PA connectivity was recently presented, together with its four fractions (Table 1), by Saura et al. (2017). It is based on previous network metrics, namely the Probability of Connectivity and the Equivalent Connected Area (Saura and Pascual-Hortal, 2007, Saura et al., 2011, Saura, 2017, Saura and De la Fuente, 2017). ProtConn is defined as the percent of a country or region covered by protected and connected lands (Table 1). ProtConn considers both intra-PA and inter-PA connectivity, i.e. it accounts for both the amount of protected land that is available within individual PAs and that reachable by moving between different PAs. In this way, ProtConn acknowledges that the amount (or percent) of protected connected lands in a country may increase in two ways. First, through the designation of larger PAs, even if this results in a single PA that encompasses several previous smaller and well inter-connected PAs (see Figs. A2 and A3 in Appendix A2). Second, through more numerous or stronger connections between different PAs. Accounting for both intra-PA and inter-PA is necessary to provide a meaningful indicator of PA connectivity where increasing values result only from desirable conservation progress. In this way, for example, the indicator will decrease in response to the replacement of a PA by multiple smaller PAs covering a smaller proportion of the originally protected land. See Appendix A2 for related details and illustrative examples in Figs. A2 and A3.

**Table 1:** Protected Connected and related fractions and indicators, all expressed as percentages. ProtConn_Bound_ and the three fractions of ProtUnconn are newly presented here, while the other indicators were introduced by Saura et al. (2017). In this table, and throughout the rest of the article, the fractions of ProtUnconn are expressed as a percentage of the total land area of the country, and hence they sum to the ProtUnconn value. The fractions of ProtConn are expressed as a percentage of the ProtConn value and so they sum to 100. See Methods and Appendices B and C for further details. PA coverage (Prot) is not a connectivity indicator but it is included because of its widespread use in assessing PA systems and because it is a key benchmark for the connectivity indicators described.

Indicator name (acronym)	Description
*Indicators referring to the entire connectivity, coverage or isolation of PAs in a country*	
Protected Connected land (ProtConn)	Percentage of the country covered by connected protected lands.

Protected Connected land bounded to the possibilities of the country (ProtConn_Bound_)	Protected Connected land considering the part of PA connectivity that is in the power of the country to influence, i.e. excluding the isolation of PAs that is naturally imposed by the sea, and that due to foreign lands (out of the jurisdiction of the country). It is the same as ProtConn for countries with PAs distributed in a single landmass that is naturally and politically continuous (i.e. no PAs in land portions separated by the sea or by other nations), and higher than ProtConn otherwise. ProtConn_Bound_ is calculated as the difference between Prot and ProtUnconn[Design] (see below).

PA Coverage or Protected land (Prot)	Percentage of the country covered by PAs (either connected or not). Prot can never be smaller than ProtConn or ProtConn_Bound_.

Protected Not Connected land (ProtUnconn)	Percentage of the country covered by protected lands that are isolated. It is simply the difference between Prot and ProtConn.

*Protected Not Connected due to…* (*fractions of ProtUnconn referring to specific causes of PA isolation*)	
…the sea (ProtUnconn[Sea])	Percentage of the country land that is protected but not connected because of the natural isolation of terrestrial PAs imposed by the sea. This fraction will be higher than zero only in those countries in which PAs are distributed over multiple islands or landmasses

…foreign lands (ProtUnconn[Outland])	Percentage of the country land that is protected but not connected because of the lack of protection in foreign lands. This fraction will be higher than zero for a given country only when other nations dissect the country in several disjoint land portions in such a way that it is not possible to move between some of the country's PAs without traversing unprotected foreign lands.

…limitations in the PA system (ProtUnconn[Design])	Percentage of the country land that is protected but not connected because of limitations or deficiencies in the design of the terrestrial PA system of the country. This is the part of the PA isolation for which a country can be made accountable, i.e. that which is under the control of a country. It is the difference between Prot and ProtConn_Bound_.

*Protected Connected by moving*… (*fractions of ProtConn referring to specific components of PA connectivity*)	
…within Individual PAs (ProtConn[Within])	Percentage of the Protected Connected land that can be reached by moving only within individual PAs, i.e. how much land can be accessed by species if they move only within the limits of individual PAs.

…through Contiguous PAs (ProtConn[Contig])	Percentage of the Protected Connected land that can be reached by moving through sets of immediately adjacent (contiguous) PAs, without traversing any unprotected lands. This percentage excludes the protected land that can be reached by moving within a single PA, which is given by ProtConn[Within].

…through Unprotected lands (ProtConn[Unprot])	Percentage of the Protected Connected land that can be reached by moving through unprotected areas. It includes movements between PAs that entirely happen through unprotected lands and others that traverse unprotected lands in the initial and final stretches but that may use some protected land in between. The value of this fraction will be lower when PAs are separated by larger tracts of unprotected lands, making inter-PA movements less likely, particularly when the distances that need to be traversed through unprotected lands are large compared to the dispersal distance.

…through Transboundary Protected lands (ProtConn[Trans])	Percentage of the Protected Connected land within the country that can be reached by moving through PAs located outside the country's boundaries. It includes the effect of both transboundary PAs in the strict sense (i.e. individual PAs that extend across country boundaries) as well as of other PAs that, located outside the country, promote the connectivity between PAs in the country by acting as corridors or stepping stones between them.

ProtConn is given by the following equation:

$$\mathrm{ProtConn}=100∙\frac{\sqrt{\sum_{i=1}^{n+t}\sum_{j=1}^{n+t}a_{i}a_{j}p_{\mathrm{ij}}^{*}}}{A_{L}}$$

where *n* is the number of PAs within the country, *t* is the number of PAs in the transboundary buffer (here 500 km) outside the country's PAs, *a*_*i*_ and *a*_*j*_ are the attribute of PAs *i* and *j*, *A*_*L*_ is the maximum possible attribute value (here country land area), and *p*^⁎^_*ij*_ is the maximum product probability of all paths connecting nodes *i* and *j*. Both direct and indirect (stepping-stone) movements between PAs are accounted for by *p*^⁎^_*ij*_. By definition *p*^⁎^_*ij*_ ≥ *p*_*ij*_, since *p*_*ij*_ only accounts for direct dispersal movements; *p*^⁎^_*ij*_ will be higher than *p*_*ij*_ when some intermediate stepping-stone PAs make dispersal between *i* and *j* more likely than in the case of direct movement (not using any stepping stone) (Saura and Pascual-Hortal, 2007, Saura and Rubio, 2010). Note that the case *i* = *j* (intra-PA connectivity) is included in the sum of the ProtConn equation; *p*^⁎^_*ij*_ = 1 when *i* = *j* (i.e. it is assumed that there is no restriction on movement within PAs). The attribute of the PAs is equal to their area for those PAs within the country, and equal to 0 for the transboundary PAs outside the country. In this way, we analyze a network in which the sources and destinations of the dispersal fluxes are only those PAs within the country (those with *a*_*i*_ > 0), but in which we consider the potential role of PAs outside the country as connectors or stepping stones between PAs in the country. See Saura et al. (2017) for further details.

The ProtConn indicator can be partitioned into four fractions depicting different categories of land through which movement between protected locations may occur: ProtConn[Within], ProtConn[Contig], ProtConn[Unprot] and ProtConn[Trans]. These four fractions are described in Table 1; equations and further details for these fractions are provided in Saura et al. (2017).

### 2.5 ProtConn_Bound_: assessing the PA connectivity levels for which a country is accountable

The ProtConn indicator, as defined above, quantifies PA connectivity as the combined result of different factors influencing PA isolation. We here present an adjusted version of the ProtConn indicator, ProtConn_Bound_, which quantifies progress of a country towards PA connectivity targets by accounting only for that part of PA connectivity that is in the power of the country to influence through an adequate design and reinforcement of its PA system (the subscript _Bound_ refers to the progress that can be made within the boundaries and possibilities of the country).

ProtConn_Bound_ is obtained through the novel partitioning of the *protected not connected* land (ProtUnconn) into three fractions, each quantifying a different cause of terrestrial PA isolation (Table 1), and all expressed as a percentage of the total country land area:

$$\text{ProtUnconn}=\text{ProtUnconn}\left[\mathrm{Sea}\right]+\text{ProtUnconn}\left[\text{Outland}\right]+\text{ProtUnconn}\left[\text{Design}\right]$$

These ProtUnconn fractions are defined in Table 1; further details on the procedures used for their calculation are provided in Appendix B.

While ProtConn is equal to the difference between PA coverage (Prot) and ProtUnconn (Table 1), ProtConn_Bound_ is calculated as:

$${\text{ProtConn}}_{\text{Bound}}=\text{Prot}-\text{ProtUnconn}\left[\text{Design}\right]$$

Therefore, ProtConn_Bound_ only penalizes a country by the PA isolation that results from limitations in the design of its PA system. In other words, only what is *protected not connected* because of the responsibility of the country (ProtUnconn[Design]) decreases the amount of protected connected lands (ProtConn_Bound_) compared to the total coverage of protected land (Prot). Since, by definition ProtUnconn[Design] ≤ ProtUnconn and ProtUnconn = Prot − ProtConn, then ProtConn_Bound_ ≥ ProtConn. On the other hand, since by definition ProtUnconn[Design] ≥ 0, then ProtConn_Bound_ ≤ Prot. In words, ProtConn_Bound_ will never be higher than the PA coverage, and will never be smaller than ProtConn.

The non-adjusted ProtConn indicator will systematically undervalue PA connectivity efforts made in countries with islands or disjoint lands, biasing comparisons with those efforts made in single-landmass countries. ProtConn_Bound_ is free from such effects, providing a fair and more meaningful assessment of countries' progress on the connectivity of their terrestrial PA systems. Appendix C provides further details on the possible range of values for ProtConn_Bound_, its differences to ProtConn and their interpretation. Finally, note that ProtConn_Bound_ could be computed in the same way for other scales or units of analysis, such as ecoregions, basins or administrative units other than countries.

All the network (graph-based) analyses for obtaining the connectivity indicators described in 2.4, 2.5 were performed using the command line version of the software package Conefor (Saura and Torné, 2009), version 2.6, available from www.conefor.org

### 2.6 Global, regional and continental averages of the country-level indicators

The indicator values were aggregated at the global, continental and regional level by calculating the weighted average of the country-level indicator values (excluding Antarctica). The land area of the country was used as the weight for averaging all the indicators that were expressed as a percentage of total land area of the country (all except the four ProtConn fractions; see Table 1), while the product of the country land area and ProtConn was used as the weight for averaging those indicators expressed as a percentage of the ProtConn value (i.e. the four ProtConn fractions).

The country-level indicator values were summarized at the continental and regional level by using the country groupings of the M49 standard of the Statistics Division of the United Nations Secretariat, available at https://unstats.un.org/unsd/methodology/m49/ (accessed May 2017). Regional and continental values are, therefore, influenced by these country groupings, such as the Russian Federation being included in Europe (continent) and Eastern Europe (region), Greenland being included within America (continent) and Northern America (region), or the United States Minor Outlying Islands being included in Oceania (continent) and Micronesia (region), among other examples. If aggregated indicator values for other country groupings are of interest for particular applications, they can be produced using the indicator values for individual countries calculated in this study. We also considered the aggregated indicator values for the European Union (EU), given that the EU has promoted and implemented the largest international coordinated network of protected areas, the Natura 2000 network.^5^ Note however that for the EU both Natura 2000 sites and nationally designated sites are included in this assessment. The EU-level values were obtained considering the 28 countries which are currently part of the EU (EU-28), and excluding PAs and territories whose reported ISO3 in the WDPA was different from those 28 countries, even if under the sovereignty of a EU member state, as in the case of Greenland or Réunion Island.

### 2.7 Classification of countries based on the main priorities for PA connectivity

We classified all countries by their main priorities for improving or sustaining PA connectivity, as assessed through the information provided by the Protected Connected set of indicators (Table 2). This classification is partially hierarchical in that it first focuses on meeting the Aichi Target 11 element on well-connected PA systems. Although the definition and supporting material for Aichi Target 11 (CBD, 2011) does not specify how PA connectivity should be measured, we here assume that the connectivity element of the target would be met if at least 17% of the country is covered by protected and connected lands while considering the limitations to PA connectivity that are out of the control of the country (for which the country is not accountable), i.e. when ProtConn_Bound_ ≥ 17%. When there is a shortfall in ProtConn_Bound_, the 17% target can only be achieved by designating new PAs in the country (priorities A in Table 2). If the 17% target of Aichi Target 11 as measured by ProtConn_Bound_ is already met in a country, then there is no need to designate new PAs in the country to meet the connectivity element of this global target, and other actions or strategies important for connectivity should be prioritized in the country (priorities B in Table 2).

**Table 2:** Classification of country-level priorities for improving or sustaining PA connectivity, as given by the country values of the ProtConn-related indicators and fractions for a reference median dispersal distance of 10 km. The highlighted priorities, however, do not exclude the possible relevance or additional necessity of the other priorities in a given country; further details can be obtained by examining the country-level values of ProtConn_Bound_ (Fig. 2) and of its fractions as separately presented in Fig. 4. Some priorities are mutually exclusive (A vs B, A1 vs A2, B1 vs B3, B2 vs B3), but the others are not (e.g. it is possible for a country to be assigned to both B1 and B2, or to C and to any other priority). C is treated separately from the other priorities (A and B) because it involves PAs outside the control or jurisdiction of the country. PA management effectiveness for connectivity is in fact assumed by the ProtConn indicator, and it is therefore a priority for all the countries (and not just for B3). The top third (33% percentile) is used as a threshold for C, rather than the top half (i.e. above the median) as in B, because the half in B is taken only from the subset of countries which already meet ProtConn_Bound_ ≥ 17%, while the condition in C applies to all countries and hence needs to be more restrictive.

Current status of the PA system in the country	Priority	Conditions to be met (as given by the ProtConn-related indicators in the country)		Why is it a priority?	
A. There are deficiencies in the design of the PA system for connectivity that need to be addressed by…	A1. General increase of PA coverage	ProtConn_Bound_ < 17%	17% − Prot > ProtUnconn[Design]	There is a shortfall in ProtConn_Bound_ compared to the 17% reference value of Aichi Target 11. Such 17% level cannot be achieved unless new PAs are designated.	Low PA coverage (Prot) is the main reason for the shortfall. Even if the current amount of protected land was all connected (giving, in the extreme, ProtUnconn[Design] = 0), most of the pending progress towards the 17% target would remain unaddressed. In addition, when PA coverage is too low, PAs tend to be small, scattered and/or far from each other, which may result in inherently low PA connectivity.
	A2. Targeted designation of PAs in strategic locations for connectivity		17% − Prot ≤ ProtUnconn[Design]		The isolation of existing PAs (ProtUnconn[Design]), and not the lack of protected land, is the main factor behind the shortfall. Rather than increasing PA coverage in general, PAs need to be strategically designated in key locations where they can efficiently function as stepping stones or corridors between other PAs. This includes, obviously, all cases in which PA coverage (Prot), but not ProtConn_Bound_, is already above the 17% target, but also those cases with Prot below 17% but closer to that value than the amount due to the isolation of existing PAs.

B. The PA system is well designed for connectivity. Rather than in designating new PAs, the priority consists in ensuring the…	B1. Permeability of the unprotected landscapes in between PAs	ProtConn_Bound_ ≥ 17%	ProtConn[Unprot] higher than the median for the countries classified as B	The design of the PA system is satisfactory for connectivity given current global targets, since the 17% reference value for Aichi Target 11 is already met as measured by ProtConn_Bound_. From that point of view, the priority if not designating new PAs, but rather to focus on other particular aspects on which the connectivity of the existing PA system depends.	PA connectivity depends largely on the ability of species to move through unprotected landscapes, which therefore requires particular emphasis in conserving or restoring the permeability of the landscape matrix in between PAs.
	B2. Coordinated management of adjacent PAs within the country		ProtConn[Contig] higher than the median for the countries classified as B		Movement through protected lands largely depends on the possibility of traversing contiguous PAs (frequently with different designations and IUCN management categories), which would allow reaching considerably more habitat resources than what is possible within the limits of individual PAs. Therefore, the coordinated management of these different PAs for connectivity is necessary to allow them to function as an effective movement pathway.
	B3. No specific priority other than PA management effectiveness for connectivity		ProtConn[Unprot] and ProtConn[Contig] both below the median for the countries classified as B		Unlike in B1 and B2, individual PAs in the country cover large tracts of land, allowing species to reach many protected habitat resources with comparatively less need to use unprotected landscapes or to move to other adjacent PAs. The fraction ProtConn[Within] dominates the ProtConn value, and this fraction is much larger than ProtConn[Unprot] or ProtConn[Contig]. The single main priority therefore is PA management effectiveness for connectivity (avoiding limitations to connectivity within PAs).

C. Connectivity of PAs within a country depends on using transboundary PAs	C. Coordinated management of linkages with transboundary PAs	ProtConn[Trans] in the top third values (33% percentile) of this fraction for all countries		Connectivity between PAs of the country depends significantly on movement through PAs in other countries. Therefore, the connectivity of PAs in the country will benefit from a coordinated transboundary management with those PAs already existing in other countries. Note that this priority and ProtConn[Trans] fraction refer to the case in which the connectivity between two PAs of the same country is promoted by a PA in a different country; the connectivity of two PAs in different countries is not assessed here.	

## 3 Results

**Fig. 1:**
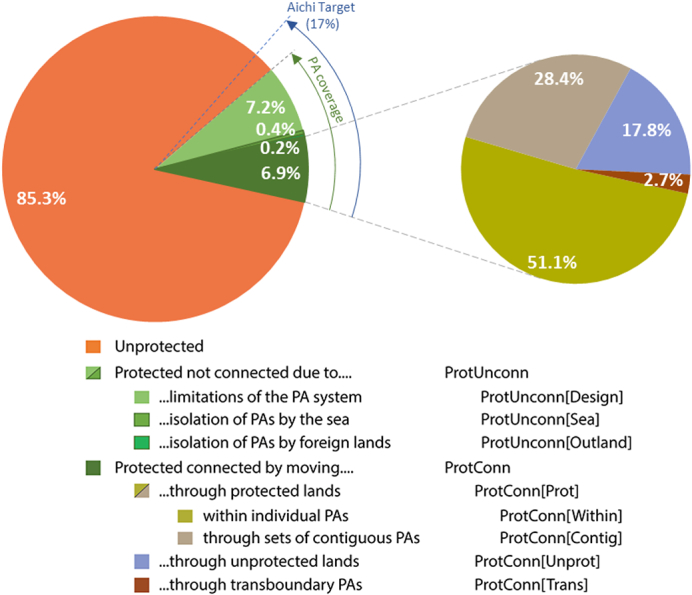
Global values of the ProtConn-related indicators for a reference median dispersal distance of 10 km. These global values have been obtained as a weighted average of the calculated country-level indicator values (see Methods). The global PA coverage is 14.7% (100% − 85.3%, or 6.9% + 0.2% + 0.4% + 7.2%), which means that protected connected lands (ProtConn = 6.9%) make up less than half of the lands under protection. However, once the PA isolation caused by the sea (ProtUnconn[Sea] = 0.4%) and by foreign lands (ProtUnconn[Outland] = 0.2%) is factored out from the country scores, ProtUnconn[Design] is 7.2% and the level of connectivity bounded to the efforts that can be really made by the countries rises to ProtConn_Bound_ = 7.5% (14.7% − 7.2%). Appendix D provides several examples of these pie charts for individual countries, including cases with larger values of ProtUnconn[Sea] and ProtUnconn[Outland]. The same global pie charts but for other dispersal distances are provided in Fig. E1 in Appendix E.

Globally, ProtConn_Bound_ is 7.5% km (area-weighted average for all countries) for a reference median dispersal distance of *d*_*med*_ = 10, which is about half of the global PA coverage of 14.7% (Fig. 1). If the isolation of the country's terrestrial PAs by the sea and foreign lands is not factored out (ProtConn), then only 6.9% of the area of the world's countries is covered by protected connected lands for *d*_*med*_ = 10 km (Fig. 1). ProtConn_Bound_ ranges from 6.9% to 9.9% and ProtConn ranges from 6.1% to 9.5% for *d*_*med*_ from 1 to 100 km. The majority of the difference between ProtConn_Bound_ and ProtConn is due to natural isolation of terrestrial PAs by the sea (ProtUnconn[Sea] = 0.4% for *d*_*med*_ = 10 km), with a smaller effect from foreign territory which separates a country into non-continuous land portions (ProtUnconn[Outland] = 0.2% for *d*_*med*_ = 10 km) (Fig. 1). For some countries, ProtUnconn[Sea] and ProtUnconn[Outland] are much larger than the global average. For example, for *d*_*med*_ = 10 km, ProtUnconn[Sea] = 7.3% for the Philippines, ProtUnconn[Sea] = 4.6% for Greece, ProtUnconn[Outland] = 3.6% for the USA (due to the separation of Alaska from the conterminous states), and ProtUnconn[Outland] = 3.1% for Brunei; see Appendix D for examples and further details. The global ProtConn average is lower at the country level, which is 6.9% for *d*_*med*_ = 10 km as reported above, than at the ecoregion level, which is 9.3% for *d*_*med*_ = 10 km as reported in Saura et al. (2017). This is mainly because the 233 countries here considered are, on average, larger than the 827 terrestrial ecoregions of the world. The larger the unit considered (country or ecoregion), the more difficult it tends to be to deploy a network of PAs that is well connected throughout its whole extent. This is shown by a relatively low but significant correlation between ProtConn_Bound_ and country area: *r* = − 0.136 (*p* = 0.037) with no variable transformation, and *r* = − 0.339 (*p* = 1.13e − 07) if the log of the country area is used, in both cases for *d*_*med*_ = 10 km (similar correlation values are found for ProtConn, for other distances, and for ecoregions).

**Fig. 2:**
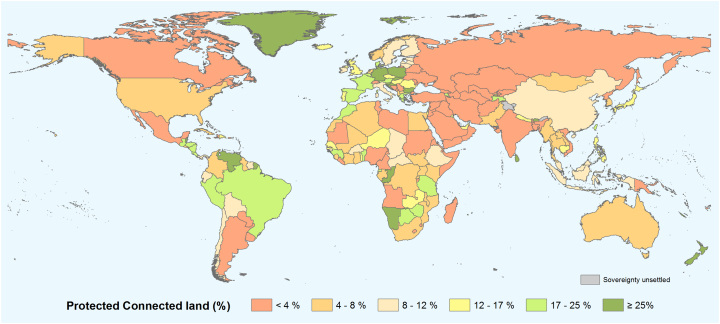
Protected Connected land for all the world's countries (ProtConn_Bound_) for a reference median dispersal distance of 10 km. ProtConn_Bound_ measures the percentage of country area covered by protected and connected lands considering the part of the PA connectivity that is in the power of a country to influence, i.e. factoring out the PA isolation due to the sea and to foreign lands. The two green classes include the countries that already meet the Aichi Target 11 element on connectivity, as assumed to be given by ProtConn_Bound_ ≥ 17%.

**Fig. 3:**
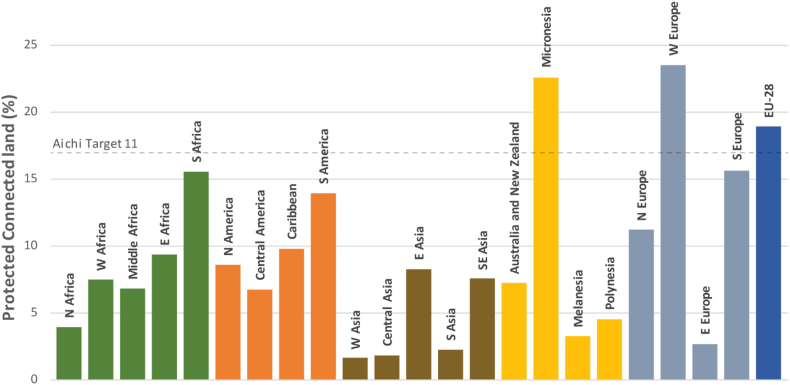
Protected Connected indicator considering the part of the PA connectivity that is in the power of a country to influence (ProtConn_Bound_) for all regions of the world and for the European Union (EU-28) for a reference median dispersal distance of 10 km. Note that the Russian Federation is included within Eastern Europe, which has a large influence on the values for this region (see Methods). Regional ProtConn_Bound_ values for other dispersal distances are shown in Fig. E2 (Appendix E). Continental-level ProtConn_Bound_ values are provided in Fig. F1 (Appendix F).

The level of PA connectivity is quite uneven across countries (Fig. 2) and regions (Fig. 3). Only 30.5% of countries currently meet the Aichi Target 11 element on well-connected PA systems, as given by ProtConn_Bound_ ≥ 17% for *d*_*med*_ = 10 km (Fig. 2). Five countries meet the 17% target as measured by ProtConn_Bound_ but not as measured by ProtConn (Appendix D). This means that these countries meet the target only when the isolation of terrestrial PAs by the sea and other countries is factored out. Even if the very large median dispersal distance *d*_*med*_ = 100 km is considered, only 38.6% of the countries meet the Aichi Target 11 connectivity element as measured by ProtConn_Bound_.

At the regional level, only Micronesia, Western Europe and the European Union already meet the 17% target for *d*_*med*_ = 10 km (Fig. 3). Southern Africa, South America and Southern Europe are close to this target (Fig. 3), with ProtConn_Bound_ values above 17% for *d*_*med*_ = 100 km (Fig. E2 in Appendix E). The average ProtConn_Bound_ = 22.6% for the region of Micronesia and *d*_*med*_ = 10 km (Fig. 3) is highly influenced by the Prot = ProtConn_Bound_ = 100% value in the United States Minor Outlying Islands. All other countries in Micronesia have a ProtConn_Bound_ value below the regional average, although three of them (Palau, Kiribati and Guam) also have ProtConn_Bound_ above 17% for *d*_*med*_ = 10 km. The EU scores higher in ProtConn_Bound_ that any of the five continents (Fig. F1 in Appendix F). The lowest regional values are found in most of Asia, Eastern Europe, Northern Africa, Melanesia and Polynesia (Figs. 3 and E2). The values for Eastern Europe are highly influenced by the low ProtConn_Bound_ = 1.5% for the Russian Federation, which is included within this region (see Methods); the rest of the countries of Eastern Europe, which all together cover a much smaller area than the Russian Federation, have an aggregated ProtConn_Bound_ = 14.3% for *d*_*med*_ = 10 km.

**Fig. 4:**
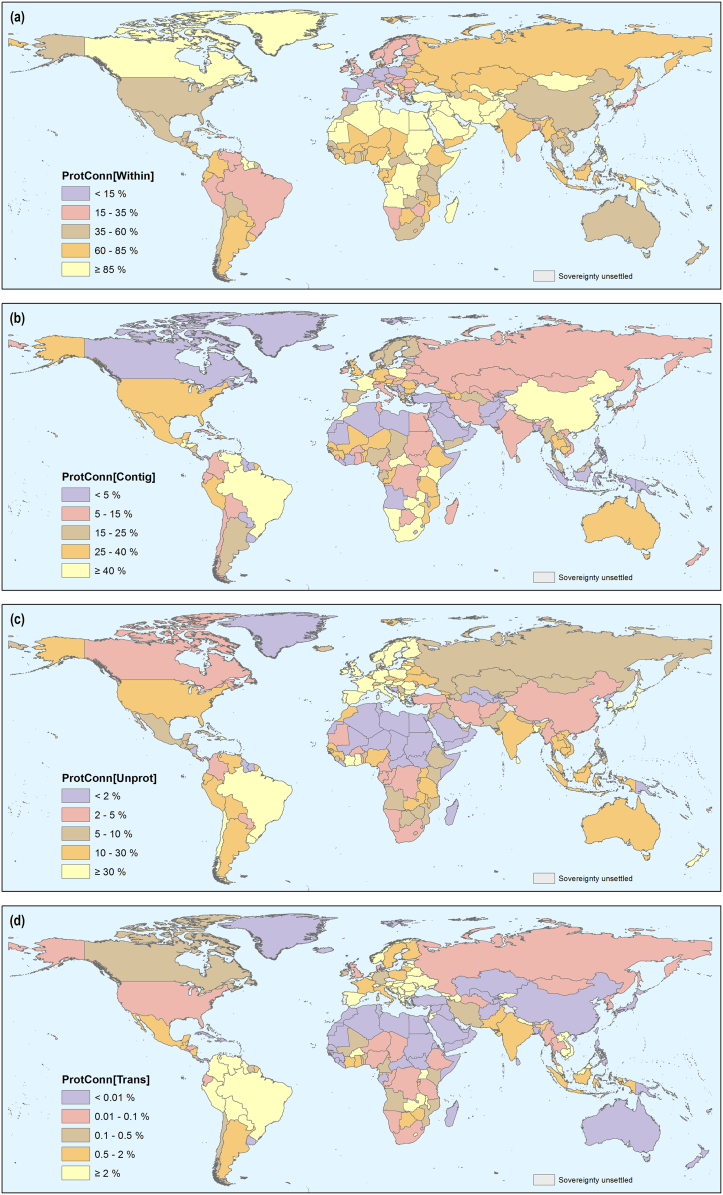
Country values of the four fractions of the Protected Connected indicator (ProtConn), which assess the percentage of the protected connected land in a country that (a) can be reached within individual PAs: ProtConn[Within], (b) can be reached by moving through adjacent PAs: ProtConn[Contig], (c) depends on movement through unprotected lands: ProtConn[Unprot], (d) depends on transnational linkages, i.e. on using PAs outside a country when moving between two PAs of the country: ProtConn[Trans]. All values correspond to a reference median dispersal distance of 10 km. See Table 1 for a more detailed description of these fractions.

**Fig. 5:**
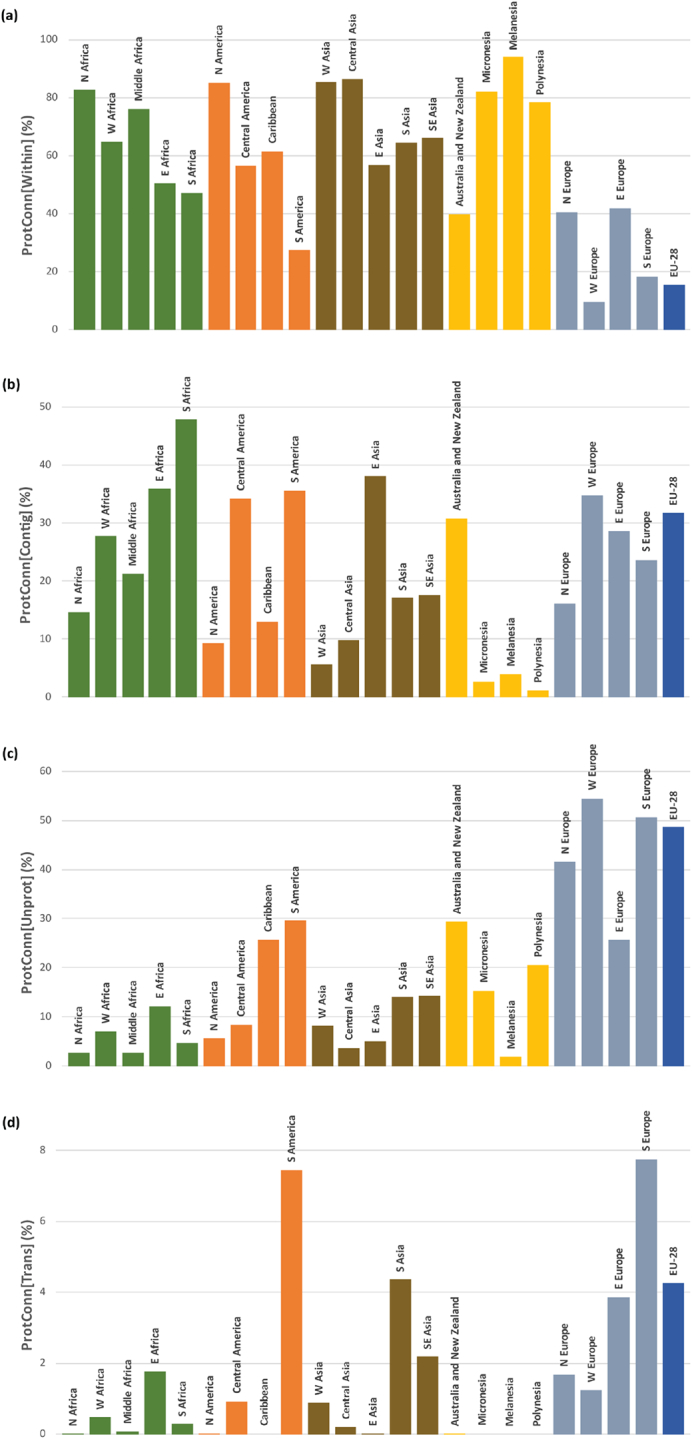
Values of the four ProtConn fractions for all regions, assessing the percentage of the protected connected land that (a) can be reached within individual PAs: ProtConn[Within], (b) can be reached by moving through adjacent PAs: ProtConn[Contig], (c) depends on movement through unprotected lands: ProtConn[Unprot], (d) depends on transnational linkages, i.e. on using PAs outside a country when moving between two PAs of the country: ProtConn[Trans]. All values correspond to a reference median dispersal distance of 10 km. Table 1 provides a more detailed description of these fractions. Continental-level values are provided in Appendix F.

The four fractions of the ProtConn indicator deliver relevant information on the characteristics of the PA systems in each country. In Europe is where the connectivity of PAs is more dependent on the possibility of movement through unprotected landscapes, as quantified by the ProtConn[Unprot] fraction (Figs. 4c and 5c). Other countries like Brazil or New Zealand are in a similar situation, with high ProtConn[Unprot] values (Fig. 4c), although not as high as in many European countries. In several countries of Southern and Eastern Africa, South and Central America and Europe, and in China, the possibility of reaching larger amounts of protected land significantly depends on the possibility of traversing contiguous PAs, often with different designations and IUCN management categories, as indicated by larger values of the ProtConn[Contig] fraction (Figs. 4b and 5b). In countries with larger individual PAs there is comparatively less need to move to other nearby/adjacent PAs or through unprotected landscapes to reach a certain amount of protected land. This is the case for Canada, Greenland, large parts of Africa (particularly Northern and Middle Africa) and Asia (particularly Western and Central Asia), as indicated by the higher values of the ProtConn[Within] fraction (Figs. 4a and 5a). At the other extreme, ProtConn[Within] is particularly low in most of Europe (Figs. 4a and 5a). In South America, Southern Europe, Eastern Europe, the European Union, and Southern Asia the connectivity between a country's PAs depends to a larger extent on movement through PAs in other countries, indicated by higher ProtConn[Trans] values compared to other regions (Figs. 4d and 5d). In the case of Southern Asia, this result is heavily driven only by Nepal, in which ProtConn[Trans] = 26.4% (see Appendix G7), the third highest in the world (only after the Czech Republic and Portugal), and more than ten times larger than in any other country in Southern Asia.

**Fig. 6:**
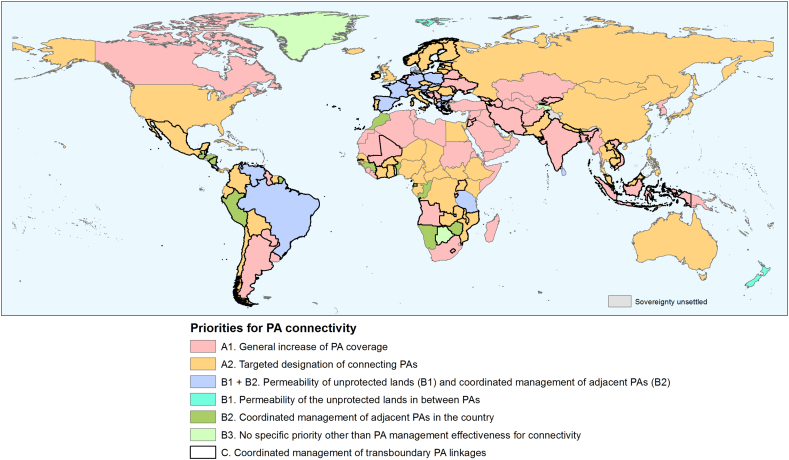
Priorities for improving or sustaining PA connectivity in each country. These priorities are identified as a function of the values of the ProtConn-related indicators (Table 2). The highlighted priorities, however, do not necessarily exclude the additional relevance or necessity of the other priorities in a given country, even if to a lower extent; more detailed information in this regard can be obtained by examining the country values of ProtConn_Bound_ (Fig. 2) and of the ProtConn fractions as separately presented in Fig. 4. Some priorities are mutually exclusive (A vs B, A1 vs A2, B1 vs B3, B2 vs B3), but the others are not (e.g. it is possible for a country to be assigned to both B1 and B2, or to C and to any other priority). In fact, as can be noticed in this figure, the large majority of the countries that have B1 as a priority also have B2 as a priority. PA management effectiveness for connectivity is an assumption of the ProtConn indicator, and it is therefore a priority for all countries (and not just for those in B3). Appendix G gives some illustrative examples of countries under the different types of priorities, showing a map of the PA system in the country together with the indicator values for that country.

The information provided by these four ProtConn fractions is summarized and integrated with the values of ProtConn_Bound_ (taking as a reference the 17% of Aichi Target 11) and of other related indicators, in a country classification of priorities for PA connectivity that shows contrasting needs across the world, as shown in Fig. 6 and with a few detailed examples in Appendix G. Europe in particular stands out as region where many countries need to focus on both ensuring the permeability of unprotected landscapes between PAs and on coordinating the management of different PAs to support coherent connecting pathways through sets of contiguous PAs within the country as well as through transboundary PAs (Fig. 6). This is also the case in countries such as Brazil (Fig. 6). In other regions, particularly North America, Asia, and large parts of Africa (Fig. 6), there is still a substantial need to designate new PAs to meet Aichi Target 11 on well-connected PA systems and to promote the longer-term traversability and self-sufficiency of the PA network.

## 4 Discussion

### 4.1 Protected Connected indicator: interpretation, caveats, insights and further enrichment

The ProtConn set of indicators provides a useful and multifaceted view of the design, structural performance, progress and needs of PA systems regarding connectivity. It is however necessary to adequately understand its assumptions and limitations to properly interpret its results and messages.

#### 4.1.1 Effective conservation management of PAs is a prerequisite for their connectivity performance

ProtConn assumes that PAs are effectively managed for connectivity, i.e. that there are no important barriers for species movements and other ecological flows within PAs. Given that this is an assumption of the indicator, ProtConn cannot identify where more effective conservation management of PAs is necessary; from the indicator perspective, this is to be understood as a priority for all PAs and countries. More importantly, management effectiveness is the key expectation once PAs are designated; without it, it is not possible to achieve the conservation objectives for which PAs are declared (Watson et al., 2014). If PAs are not conserved and managed effectively, and connectivity is constrained even within protected lands, the full connectivity potential of the PA system will not be fulfilled. Therefore, in such a case the actual connectivity levels will be lower than those reported by the indicator. Efforts have been made in assessing PA conservation and management effectiveness (Geldmann et al., 2013, Juffe-Bignoli et al., 2014, Coad et al., 2015, Gray et al., 2016, UNEP-WCMC and IUCN, 2016). However, no global study has, to our knowledge, focused specifically on the connectivity performance of PA management. This remains as a potential avenue for further research, ultimately allowing further enrichment of the ProtConn indicator with this type of information in the future.

#### 4.1.2 The heterogeneity of the landscape matrix

In its present form, ProtConn does not consider the heterogeneity of unprotected landscapes. The indicator, as currently presented, considers PAs to be favourable for movement (see previous section) and unprotected landscapes to be all equally hostile. The likelihood of a successful movement between PAs is assumed to decrease with the distance to be traversed through unprotected land. In some cases, however, there might be unsurmountable barriers to the movement of some species, such as highways, urbanized areas or intensified agricultural lands, which would impose additional limitations to PA connectivity not captured by the indicator. This is a limitation of the indicator as currently presented; it does not account for the effects that particular land covers or land uses may have on species movements.

Such a matrix-independent assessment, however, also comes with the advantage of providing a more general view on PA systems that is not dependent on the particularities of certain species. This is of interest because what is favourable for the movement of some species (e.g. a bear or a capercaillie dispersing along a wide forest corridor) may be a barrier for others (e.g. an open-habitat butterfly or a steppe bird). Obviously, species-specific traits and needs should be considered in more detailed, focused connectivity studies for particular taxa and habitats; such detailed studies are valuable and indispensable. However, we here intend to provide a coarser overarching picture on how PA systems are designed to support connectivity (considering the spatial arrangement, number and size of PAs) and whether they are self-sufficient to do so in the long term, rather than focusing on species-specific perceptions and current conditions for connectivity of the landscape matrix. We acknowledge that unprotected landscapes may in some cases have good conditions for connectivity, approaching those in PAs. ProtConn, however, evaluates how the PA system is able, by itself, to support connectivity in the long run. Unprotected landscapes, even if they have good conditions today, are often more susceptible to developing increased resistance to species movement through degradation and land use change, and likely to experience higher mortality rates of dispersing individuals due to various pressures (e.g. poaching, road kills, etc.) in the future (DeFries et al., 2005, Hansen and DeFries, 2007, Goetz et al., 2009, Wade and Theobald, 2010, Laurance et al., 2012, Piquer-Rodríguez et al., 2012, Barber et al., 2014, De Moraes et al., 2017). The intensity of these threats to landscape permeability can be highly variable across countries, as well as in different landscapes within a country. For instance, remote, inaccessible areas may retain good conditions for connectivity even in the absence of dedicated conservation efforts. The opposite may be true in areas close to the deforestation front, or in areas where agricultural intensification or urban expansion may more rapidly result in the loss of the remaining natural vegetation. More detailed national assessments should evaluate, and incorporate in their planning, the interplay between current landscape conditions, potential land cover change, and the identification of areas where conservation efforts are more urgent.

There are good examples, at the country or regional level, of accounting for landscape matrix resistance in the analysis of connectivity between PAs or conservation units, such as those by Rabinowitz and Zeller (2010), Gurrutxaga et al. (2011), Belote et al. (2016), Blázquez-Cabrera et al. (2016) and Dickson et al. (2017). These studies have mapped connectivity patterns or priority areas, but have not provided an indicator that can be used to assess PA connectivity targets, such as the connectivity element of Aichi Target 11. The methods tested and successfully applied in these studies could support an enriched version of the ProtConn indicator that accounts for landscape heterogeneity between PAs. This is part of our planned future work.

#### 4.1.3 ProtConn as a flagging system for connectivity priorities

Although ProtConn does not consider landscape matrix heterogeneity, it is able to pinpoint, through the ProtConn[Unprot] fraction, countries (or regions) in which the connectivity of the PA system is more dependent on movement through, and on the permeability of, unprotected landscapes. Such dependency will be lower in countries where a large majority of the protected land can already be reached by moving only within or through contiguous PAs, as captured by ProtConn[Within] and ProtConn[Contig]. Similarly, ProtConn does not use as an input any information on whether different PAs are being managed in a coherent and coordinated way regarding connectivity, but it is able to highlight, through the ProtConn[Contig] fraction, countries or regions in which it is more critical to ensure the coordinated management of adjacent PAs. Regarding these aspects, the ProtConn set of indicators can be seen as a flagging system highlighting where certain strategies need to focus more management efforts, rather than informing about whether such management efforts are actually being put in place. Therefore, the priorities here highlighted should be complemented with evaluations of the implementation and effectiveness of the related management efforts in a given country or region.

#### 4.1.4 Connectivity, representativeness and other aspects of PA performance

The ProtConn indicators provide important, policy relevant information for evaluating progress on the connectivity of PA systems. However, more comprehensive assessments must also consider other aspects of PA performance such as ecological representativeness, protection of areas of particular importance for biodiversity and ecosystem services, and effective and equitable management (CBD, 2010). Several other global biodiversity indicators have been developed and are in use by the CBD for some of these requirements, such as PA coverage of ecoregions and key biodiversity areas, and some progress is being made on assessments of PA management effectiveness (Juffe-Bignoli et al., 2014, CBD, 2016, BIP, 2016).

Regarding representativeness, it is worth noting that a certain ProtConn value in a country, as here reported, may be different from the indicator value that may be obtained if calculated for a particular habitat type. The value for a specific habitat would be lower than the overall country-level ProtConn if this habitat is not well represented in the PA system or if, though represented, there are no PAs that can support the connectivity between the different habitat patches. Therefore, a given country may reach the Aichi Target 11 for connectivity overall, but this does not mean that the target is met for each particular habitat, species or ecosystem; some of them may lag significantly behind that level. For example, we here find Brazil to have ProtConn_Bound_ = 17.1% for *d*_*med*_ = 10 km, i.e. just above the 17% level of Aichi Target 11 for 2020 (Fig. 2). However, Saura et al. (2017) reported PA connectivity to be much higher in the Amazon than in the Atlantic forest ecoregions of Brazil, agreeing with the modest levels of connectivity for jaguars provided by PAs in the Brazilian Atlantic forests as found by Diniz et al. (2017). Fajardo et al. (2014) also found a similar pattern in Peru, with the coastal region having significantly lower levels of protection and PA connectivity than the Amazon region of this country. In general, these issues could be further examined through separate connectivity analyses for each of these regions, species, habitats or ecosystem types, although this is out of the scope of this study.

#### 4.1.5 The importance of the quality of the information on PAs

As for any indicator, ProtConn is influenced by the completeness, quality and update frequency of the underlying data on PAs (Visconti et al., 2013, Han et al., 2017). If the WDPA data does not accurately reflect the reality of the current PA system in a country – a concern in some data-poor regions – indicators such as ProtConn will be impacted. For instance, about 6% of the PAs in the WDPA were provided as points with a reported area but no specified boundaries. As in other studies (e.g. Gray et al., 2016, UNEP-WCMC and IUCN, 2016), we dealt with this issue by generating circular buffers around the points with an area equal to the reported one. The actual boundaries of these PAs, however, may significantly differ from such a circular shape. For instance, some PAs may follow dendritic patterns along rivers or other physical features; in such cases, the ProtConn values calculated from the circular buffers will underestimate the actual connectivity provided by these PAs. The magnitude of this potential underestimate is, however, much lower than that which would result from excluding these point PAs from the analysis. In any case, ongoing efforts to reduce the number of PAs without a precise polygon delineation in the WDPA will benefit the resultant indicator values in the future.

### 4.2 Country-level priorities for PA connectivity: what needs to be done and where?

#### 4.2.1 Considerable global shortfalls in PA connectivity, but very different national priorities

Our results have shown that considerable efforts are needed to improve PA connectivity globally. Currently, the amount of protected connected land is only about half of the area under protection, and most countries lag behind the Aichi Target 11 element on well-connected PA systems. Despite this situation, connectivity was not considered in most of the PA gap assessments for the countries or regions reviewed in Ervin et al. (2010).

At the same time, there are large differences in the current status of PA systems in different countries, as reflected by the values of the ProtConn-related indicators. These differences translate into distinct priorities regarding the strategies and actions required to improve the performance or unlock the full potential of PA systems for connectivity (Table 2, Fig. 6 and Appendix G), as discussed next.

#### 4.2.2 Expansion of the PA systems is needed in most of the countries, and the strategic location of the new PAs is crucial

In those countries below the Aichi Target 11 element on well-connected PA systems, which we here understand to be met if ProtConn_Bound_ ≥ 17%, shortfalls can only be addressed by designating new PAs (priorities A in Table 2 and Fig. 6). A very low PA coverage in a country inevitably leads to either (i) a scattered pattern of distant PAs that are poorly connected to each other (Santini et al., 2016), such as in Turkey (Appendix G1), Iraq, Uruguay or Ukraine, or to (ii) PAs or clusters of PAs that are well connected locally but that, all together, can only provide a small amount of connected area, as in Djibouti, Mali or Bangladesh. Neither of these two situations can deliver a high ProtConn_Bound_ value, which can never be above PA coverage; large-scale designation of new PAs would be needed in the country (priority A1).

As the deficits in PA coverage are reduced, the strategy needs to progressively shift towards an increasing focus on filling key PA connectivity gaps in the country (priority A2). These gaps could be addressed through the designation of strategically located PAs to link the rest of the PAs by acting as corridors or stepping stones between them. This is a priority in countries like USA, Mexico, the Russian Federation, China, Australia, or Cameroon (Appendix G2), among others (Fig. 6). A compelling and remarkable example of successful PA system design for connectivity is that of Bhutan (Wangchuk, 2007), a case that is further described in Appendix G5 and that could serve as a reference for other countries.

The case of the USA may be illustrative. Strategic linkages between PAs were also highlighted as a conservation priority for the USA by Minor and Lookingbill (2010), particularly for small mammals, in their analysis of PA connectivity in that country. Minor and Lookingbill (2010) suggested that, for these species, new acquisitions should be located so that they serve as stepping stones between larger PAs. Interestingly, the USA is the country whose ProtConn_Bound_ value is closest to the global average (ProtConn_Bound_ = 7.5% in both cases). Therefore, the USA might be regarded as representing a typical situation of the PA systems in the world's countries. It is possible to treat the globally aggregated values of the ProtConn indicators as those for the individual countries in Table 2 and Fig. 6. By doing so, we find that PA expansion through targeted designation of PAs in strategic locations for connectivity (A2 priority) is the most important priority for the overall global network of PAs, as is the case for the USA and other countries. The ProtConn_Bound_ level of 7.5% is however reached in the USA with a PA coverage of 13.0%, which is lower than the global 14.7% PA coverage. This suggests a higher relative connectivity efficiency of the spatial arrangement of PAs in the USA than in the global average, even if the resultant percentage of reachable protected land, and the main priority (A2), is the same in both cases.

Finally, it is worth noting that there are potential synergies between the country connectivity priorities proposed here and other PA conservation goals and criteria. Many countries for which we suggest designation of new PAs to increase connectivity were also highlighted by Pouzols et al. (2014) as top global priorities for PA network expansion to protect vertebrate species ranges and ecoregions. This is the case, for instance, for Mexico, Panamá, Colombia, Ecuador, Madagascar, Indonesia, Papua New Guinea, Malaysia, Vietnam, Nepal and the Philippines.

#### 4.2.3 Countries that do not need PA expansion to reach the 17% level but that do require the permeability of their unprotected lands and the coordinated management of national and transnational PAs

There is a comparatively smaller set of countries where the 17% target for 2020 as measured by ProtConn_Bound_ has already been met (priorities B in Table 2). This is the case for many countries in the European Union and Micronesia, some countries in South and Central America and in Southern Africa, and for Greenland or New Zealand (Fig. 2 and Appendix G). This is a positive result that reflects a good design of these countries' PA systems from a connectivity perspective, as also noted by Maiorano et al. (2015) for the EU. However, this result does not mean that effective PA connectivity in the country will be guaranteed without paying attention to other important management-related aspects as highlighted in Fig. 6. These aspects are the permeability of unprotected landscapes, the coordinated management for connectivity of neighbouring PAs, and transboundary connectivity (Table 2), in addition to the effective conservation management of PAs for connectivity that, as noted above, is a priority in all cases. The potential of a well-designed PA system for connectivity will not be fulfilled in the absence of adequate management strategies focusing on the highlighted priorities for each country (Fig. 6).

For instance, PA connectivity in the European Union is particularly dependent on the possibility of movement through unprotected landscapes, much more than in any other region (Fig. 5c); see an example for Spain in Appendix G3. This is because, in the EU, PAs are generally small (low ProtConn[Within] values), as compared to other regions or continents (see Figs. 4a and 5a). This has also been reported in previous studies (Maiorano et al., 2007, EEA, 2012, Santini et al., 2016). Because they are small, and they are embedded in unprotected landscapes, it is unlikely that they are sufficient to ensure, individually, the conservation goals for which they were declared. Meeting these goals will only be possible if PAs function as an effective network of linked sites (Jongman et al., 2004, Von Haaren and Reich, 2006, Maiorano et al., 2007), which necessarily involves the conservation or restoration of green infrastructure elements in the intervening unprotected landscapes (Navarro and Pereira, 2015). Promoting the permeability of the lands located in between PAs is, therefore, a priority for the EU, and this needs to involve multiple sectors and actions, from the agro-environmental measures of the Common Agricultural Policy (Donald and Evans, 2006, Navarro and Pereira, 2015) to the defragmentation of transport infrastructure in key locations for connectivity (Gurrutxaga et al., 2011). Other countries like New Zealand (Appendix G4) or Brazil may be in a similar situation (Figs. 4c and 6), though to a lesser degree since they do not suffer from such small ProtConn[Within] values as the EU (Fig. 4a).

An updated, realistic and complete management plan is key to the conservation success of a PA. However, the frequent separate planning for each individual PA is unlikely to deliver the desired connectivity outcomes. We have shown that, for many countries in the world, coordinated management across different PAs is key to allow for larger-scale ecological processes across connecting pathways that can be formed by the concatenation of nearby sites through PA networks. Such coordinated management involves national-level strategies supporting the coherent functioning of multiple PAs within the country and/or transnational plans reinforcing the linkages across borders (Opermanis et al., 2012, Santini et al., 2016); see an example for Nepal in Appendix G7. In many countries and regions, such as South America and Europe, both within-country and transnational coordinated management of PAs are highlighted as a priority to unlock the full connectivity potential of the PA system (Fig. 6). This finding further supports the importance of improving transboundary PA connectivity, as was highlighted by Opermanis et al. (2012) in Europe and by Santini et al. (2016) globally. In particular, Santini et al. (2016) showed that continental PA networks perform worse than national networks, and that transboundary connectivity is often weak and should be improved. These results are also in conceptual agreement with Pouzols et al. (2014), who showed that global to continental scale conservation planning and international cooperation is vital for reaching high quality conservation outcomes. The expansion of PAs by considering only national priorities would produce more fragmented PA networks than when the global or transnational context is taken into account (Pouzols et al., 2014).

The country-level priorities here suggested should be interpreted and adjusted at the national level, considering the more detailed context and management challenges in each particular situation. This is also the case when defining which particular measures should be applied to improve the connectivity of PAs and unprotected landscapes. For instance, while the conservation benefits of corridors have been shown to largely outweigh their potential disadvantages (Gilbert-Norton et al., 2010, Haddad et al., 2014), specific measures aimed at improving connectivity may in certain cases result in undesired outcomes. This can include the spread of invasive species, diseases, or predators, the proliferation of edge-affiliated species, or an increase in wildlife-human conflicts in some areas (Madden, 2004, Chetkiewicz et al., 2006, Hilty et al., 2006, Haddad et al., 2014, Resasco et al., 2014). The strategies considered here (expansion of PAs, coordinated management of PAs, etc.) do not exclude, and in fact must give full consideration to, context-specific consideration of the measures needed to achieve the desired levels of connectivity and other conservation goals. These measures should be designed and applied in the right form, and in the right places, to promote some processes (e.g. gene flow for species of conservation concern) while halting others (e.g. pressure from predators or spread of invasive species).

#### 4.2.4 ProtConn allows evaluating shortfalls and national priorities in other current or future targets

It is important to note that we designed this country classification of priorities with reference to the 17% level of Aichi Target 11 for year 2020, which is a global political target. Although all CBD Parties committed to Aichi Target 11, individual countries or regions are free to set their own targets, as has happened in some cases for PA coverage (Butchart et al., 2015). The application of the overall 17% target at country (or ecoregion) level is a common approach for analyses of PA coverage, representativeness and connectivity (Butchart et al., 2015, UNEP-WCMC and IUCN, 2016, Saura et al., 2017). It does not consider, however, specific targets that may be set by countries or regions. In addition, a different global target may be set for the post-2020 period. For example, Dinerstein et al. (2017) suggest the goal of protecting 50% of the Earth's land mass to address the species-extinction crisis and conserve global ecological heritage. Therefore, our set of priorities should be seen as an indicative roadmap to 2020, or as the pending efforts for that target. The same approach and set of ProtConn indicators can be used to provide a modified or updated classification of priorities and pending efforts when more ambitious or different targets emerge at national, regional or global scale.

### 4.3 Conclusions

We have quantified, through a novel set of ProtConn-related indicators, how well PA systems are designed for connectivity in all countries. Using these indicators, we have highlighted where additional efforts are required to reach Aichi Target 11 for connectivity, as well as key factors potentially contributing to or limiting PA connectivity in each country. This has allowed us to identify the main priorities for improving or sustaining PA connectivity in each country, as an indicative roadmap that should facilitate and stimulate targeted action to 2020 and beyond. We hope this information can help address gaps in national strategies and action plans, where PA connectivity may not be explicitly considered. We also contribute to focus efforts and resources on those interventions – e.g. general or targeted PA expansion, management of the landscape matrix, or transboundary cooperation – that would most benefit PA connectivity in each country and globally. The final aim of these interventions would be to produce more efficient PA networks, and thus contribute to the persistence of biodiversity in the face of climatic and environmental changes.

While the global PA system is approaching the CBD's 17% terrestrial protection target for 2020 in terms of coverage (currently at 14.7%), it still falls substantially short of the CDB requirement on connectivity. The current amount of protected connected land is only about half of the area under protection, and most countries lag significantly behind the Aichi Target 11 connectivity element. While the 17% protected connected target may be challenging to achieve by 2020 in many regions, our analysis also highlights success stories regarding the design of PA systems for connectivity. This is the case of the European Union and Micronesia, where the target has already been met regionally (but not for all the countries); of other regions, such as South America or Southern Africa, which are not far away from the target; and of compelling examples for specific countries such as Bhutan.

Most, if not all, of this progress has so far been achieved in the absence of agreed quantitative indicators or targets for PA connectivity. With our analysis and ProtConn family of indicators, we fill an important gap in the current set of indicators for PA systems, and we aim to contribute to future efforts to promote, monitor and report progress on PA connectivity globally. Such efforts would benefit from a clearer definition and specific, measurable, time-bound target for the connectivity of PA systems, as well as better guidance on how to measure it, which we propose could be provided through ProtConn. The newly proposed ProtConn_Bound_ indicator, in particular, focuses specifically on the PA connectivity that a country has the power to influence. By doing so, it provides a policy relevant indicator at the scale at which most political decisions on the development and management of PAs networks are taken. Therefore, our contribution can be used to compare countries on an equal footing, and to guide countries in their own efforts to improve PA connectivity, for example by evaluating different scenarios of future PA system expansion. Current and future values of ProtConn_Bound_ and related indicators will be made available through the Digital Observatory for Protected Areas (DOPA) of the Joint Research Centre of the European Commission (Dubois et al., 2016), which can be accessed at http://dopa.jrc.ec.europa.eu/. This may include ProtConn updates incorporating new releases of the World Database on Protected Areas, as well as enriched or adapted versions of the indicator that relax some of its current limitations or assumptions to consider, for example, actual PA management effectiveness for connectivity or the heterogeneity of the landscape matrix in between PAs.

## Appendices A-G Supplementary data

Supplementary material Image 1

## Footnotes

1 https://www.bipindicators.net/.

2 https://www.protectedplanet.net/.

3 http://www.fao.org/geonetwork/srv/en/metadata.show?id=12691.

4 https://www.protectedplanet.net/, http://dopa-explorer.jrc.ec.europa.eu/dopa_explorer/.

5 http://ec.europa.eu/environment/nature/natura2000/index_en.htm.

## References

[bb0005] Barber C.P., Cochrane M.A., Souza C.M., Laurance W.F. (2014). Roads, deforestation, and the mitigating effect of protected areas in the Amazon. Biol. Conserv..

[bb0010] Beale C.M., Baker N.E., Brewer M.J., Lennon J.J. (2013). Protected area networks and savannah bird biodiversity in the face of climate change and land degradation. Ecol. Lett..

[bb0015] Belote R.T., Dietz M.S., McRae B.H., Theobald D.M., McClure M.L., Irwin G.H., McKinley P.S., Gage J.A., Aplet G.H. (2016). Identifying corridors among large protected areas in the United States. PLoS One.

[bb0020] Bertzky B., Corrigan C., Kemsey J., Kenney S., Ravilious C., Besançon C., Burgess N. (2012). Protected Planet Report 2012: Tracking Progress Towards Global Targets for Protected Areas.

[bb0025] BIP (2016). Open Consultation for Indicators for the Strategic Plan for Biodiversity 2011–2020: Consultation Results. https://www.bipindicators.net/resources/governance-documents/open-consultation-report.

[bb0030] Blázquez-Cabrera S., Gastón A., Beier P., Garrote G., Simón M.A., Saura S. (2016). Influence of separating home range and dispersal movements on characterizing corridors and effective distances. Landsc. Ecol..

[bb0035] Butchart S.H.M., Clarke M., Smith R.J., Sykes R.E., Scharlemann J.P.W., Harfoot M., Buchanan G.M. (2015). Shortfalls and solutions for meeting national and global conservation area targets. Conserv. Lett..

[bb0040] Butchart S.H.M., Di Marco M., Watson J.E.M. (2016). Formulating smart commitments on biodiversity: lessons from the Aichi targets. Conserv. Lett..

[bb0045] CBD (2010). Decision UNEP/CBD/COP/DEC/X/2 Adopted by the Conference of the Parties to the Convention on Biological Diversity at Its Tenth Meeting. https://www.cbd.int/decision/cop/?id=12268.

[bb0050] CBD (2011). Strategic Plan for Biodiversity 2011–2020. Further information related to the technical rationale for the Aichi Biodiversity Targets, including potential indicators and milestones. UNEP/CBD/COP/10/INF/12/Rev.1.

[bb0055] CBD (2016). Decision CBD/COP/DEC/XIII/28: Indicators for the Strategic Plan for Biodiversity 2011–2020 and the Aichi Biodiversity Targets. https://www.cbd.int/conferences/2016/cop-13/documents.

[bb0060] Chetkiewicz C.L.B., St. Clair C.C., Boyce M.S. (2006). Corridors for conservation: integrating pattern and process. Annu. Rev. Ecol. Evol. Syst..

[bb0065] Coad L., Leverington F., Knights K., Geldmann J., Eassom A., Kapos V., Kingston N., de Lima M., Zamora C. (2015). Measuring impact of protected area management interventions: current and future use of the Global Database of Protected Area Management Effectiveness. Philos. Trans. R. Soc. B.

[bb0070] De Moraes M.C.P., de Mello K., Toppa R.H. (2017). Protected areas and agricultural expansion: biodiversity conservation versus economic growth in the Southeast of Brazil. J. Environ. Manag..

[bb0075] DeFries R., Hansen A., Newton A.C., Hansen M.C. (2005). Increasing isolation of protected areas in tropical forests over the past twenty years. Ecol. Appl..

[bb0080] Dickson B.G., Albano C.M., McRae B.H., Anderson J.J., Theobald D.M., Zachmann L.J., Sisk T.D., Dombeck M.P. (2017). Informing strategic efforts to expand and connect protected areas using a model of ecological flow, with application to the western United States. Conserv. Lett..

[bb0085] Dinerstein E., Olson D., Joshi A., Vynne C., Burgess N.D. (2017). An ecoregion-based approach to protecting half the terrestrial realm. Bioscience.

[bb0090] Diniz M.F., Machado R.B., Bispo A.A., Brito D. (2017). Identifying key sites for connecting jaguar populations in the Brazilian Atlantic Forest. Anim. Conserv..

[bb0095] Donald P.F., Evans A.D. (2006). Habitat connectivity and matrix restoration: the wider implications of agri-environment schemes. J. Appl. Ecol..

[bb0100] Dubois G., Bastin L., Bertzky B., Mandrici A., Conti M., Saura S., Cottam A., Battistella L., Martínez-López J., Boni M., Graziano M. (2016). Integrating multiple spatial datasets to assess protected areas: lessons learnt from the Digital Observatory for Protected Areas (DOPA). ISPRS Int. J. Geo-Inf..

[bb0105] EEA, 2012. Protected Areas in Europe — An Overview. European Environment Agency (EEA), Copenhagen. EEA Report No 5/2012. ISSN 1725-9177.

[bb0110] Engelhard S.L., Huijbers C.M., Stewart-Koster B., Olds A.D., Schlacher T.A., Connolly R.M. (2017). Prioritising seascape connectivity in conservation using network analysis. J. Appl. Ecol..

[bb0115] Ervin J., Sekhran N., Dinu A., Gidda S., Vergeichik M., Mee J. (2010). Protected Areas for the 21st Century: Lessons from UNDP/GEF's Portfolio.

[bb0120] Fajardo J., Lessmann J., Bonaccorso E., Devenish C., Muñoz J. (2014). Combined use of systematic conservation planning, species distribution modelling, and connectivity analysis reveals severe conservation gaps in a megadiverse country (Peru). PLoS One.

[bb0125] Geldmann J., Barnes M., Coad L., Craigie I. (2013). Effectiveness of terrestrial protected areas in reducing habitat loss and population declines. Biol. Conserv..

[bb0130] Gilbert-Norton L., Wilson R., Stevens J.R., Beard K.H. (2010). A meta-analytic review of corridor effectiveness. Conserv. Biol..

[bb0135] Goetz S.J., Jantz P., Jantz C.A. (2009). Connectivity of core habitat in the Northeastern United States: parks and protected areas in a landscape context. Remote Sens. Environ..

[bb0140] Gray C.L., Hill S.L., Newbold T., Hudson L.N., Börger L., Contu S., Hoskins A.J., Ferrier S., Purvis A., Scharlemann J.P. (2016). Local biodiversity is higher inside than outside terrestrial protected areas worldwide. Nat. Commun..

[bb0145] Gurrutxaga M., Rubio L., Saura S. (2011). Key connectors in protected forest area networks and the impact of highways: a transnational case study from the Cantabrian Range to the Western Alps. Landsc. Urban Plan..

[bb0150] Haddad N.M., Brudvig L.A., Damschen E.I., Evans D.M., Johnson B.L., Levey D.J., Orrock J.L., Resasco J., Sullivan L.L., Tewksbury J.J., Wagner S.A., Weldon A.J. (2014). Potential negative ecological effects of corridors. Conserv. Biol..

[bb0155] Han X., Josse C., Young B.E., Smyth R.L., Hamilton H.H., Bowles-Newark N. (2017). Monitoring national conservation progress with indicators derived from global and national datasets. Biol. Conserv..

[bb0160] Hansen A.J., DeFries R. (2007). Ecological mechanisms linking protected areas to surrounding lands. Ecol. Appl..

[bb0165] Hanski I., Ovaskainen O. (2000). The metapopulation capacity of a fragmented landscape. Nature.

[bb0170] Hilty J.A., Lidicker W.Z., Merenlender A. (2006). Corridor Ecology: The Science and Practice of Linking Landscapes for Biodiversity Conservation.

[bb0175] IUCN, UNEP-WCMC (2016). The World Database on Protected Areas (WDPA). http://www.protectedplanet.net.

[bb0180] Jongman R.H., Külvik M., Kristiansen I. (2004). European ecological networks and greenways. Landsc. Urban Plan..

[bb0185] Juffe-Bignoli D., Burgess N.D., Bingham H., Belle E.M.S., de Lima M.G. (2014). Protected Planet Report 2014.

[bb0190] Krosby M., Tewksbury J., Haddad N.M., Hoekstra J. (2010). Ecological connectivity for a changing climate. Conserv. Biol..

[bb0195] Kuussaari M., Bommarco R., Heikkinen R.K., Helm A., Krauss J., Lindborg R., Öckinger E., Pärtel M., Pino J., Rodà F., Stefanescu C., Teder T., Zobel M., Steffan-Dewenter I. (2009). Extinction debt: a challenge for biodiversity conservation. Trends Ecol. Evol..

[bb0200] Laurance W.F., Useche D.C., Rendeiro J., Kalka M., Bradshaw C.J.A. (2012). Averting biodiversity collapse in tropical forest protected areas. Nature.

[bb0205] Leroux S.J., Krawchuk M.A., Schmiegelow F., Cumming S.G., Lisgo K., Anderson L.G., Petkova M. (2010). Global protected areas and IUCN designations: do the categories match the conditions?. Biol. Conserv..

[bb0210] Madden F. (2004). Creating coexistence between humans and wildlife: global perspectives on local efforts to address human–wildlife conflict. Hum. Dimens. Wildl..

[bb0215] Maiorano L., Falcucci A., Garton E.O., Boitani L. (2007). Contribution of the Natura 2000 network to biodiversity conservation in Italy. Conserv. Biol..

[bb0220] Maiorano L., Amori G., Montemaggiori A., Rondinini C., Santini L., Saura S., Boitani L. (2015). On how much biodiversity is covered in Europe by national protected areas and by the Natura 2000 network: insights from terrestrial vertebrates. Conserv. Biol..

[bb0225] Minor E.S., Lookingbill T.R. (2010). A multiscale network analysis of protected-area connectivity for mammals in the United States. Conserv. Biol..

[bb0230] Navarro L., Pereira H. (2015). Rewilding European Landscapes.

[bb0235] Opermanis O., MacSharry B., Aunins A., Sipkova Z. (2012). Connectedness and connectivity of the Natura 2000 network of protected areas across country borders in the European Union. Biol. Conserv..

[bb0240] Piquer-Rodríguez M., Kuemmerle T., Alcaraz-Segura D., Zurita-Milla R., Cabello J. (2012). Future land use effects on the connectivity of protected area networks in southeastern Spain. J. Nat. Conserv..

[bb0245] Pouzols F.M., Toivonen T., Di Minin E., Kukkala A.S., Kullberg P., Kuusterä J., Lehtomäki J., Tenkanen H., Verburg P.H., Moilanen A. (2014). Global protected area expansion is compromised by projected land-use and parochialism. Nature.

[bb0250] Rabinowitz A., Zeller K.A. (2010). A range-wide model of landscape connectivity and conservation for the jaguar, *Panthera onca*. Biol. Conserv..

[bb0255] Rands M.R.W., Adams W.M., Bennun L., Butchart S.H.M., Clements A. (2010). Biodiversity conservation: challenges beyond 2010. Science.

[bb0260] Resasco J., Haddad N.M., Orrock J.L., Shoemaker D., Brudvig L.A., Damschen E.I., Tewksbury J.J., Levey D.J. (2014). Landscape corridors can increase invasion by an exotic species and reduce diversity of native species. Ecology.

[bb0265] Santini L., Saura S., Rondinini C. (2016). Connectivity of the global network of protected areas. Divers. Distrib..

[bb0270] Saura S. (2017). Node self-connections in network metrics. Ecol. Lett..

[bb0275] Saura S., De la Fuente B., Gergel S.E., Turner M.G. (2017). Connectivity as the amount of reachable habitat: conservation priorities and the roles of habitat patches in landscape networks. Learning Landscape Ecology.

[bb0280] Saura S., Pascual-Hortal L. (2007). A new habitat availability index to integrate connectivity in landscape conservation planning: comparison with existing indices and application to a case study. Landsc. Urban Plan..

[bb0285] Saura S., Rubio L. (2010). A common currency for the different ways in which patches and links can contribute to habitat availability and connectivity in the landscape. Ecography.

[bb0290] Saura S., Torné J. (2009). Conefor Sensinode 2.2: a software package for quantifying the importance of habitat patches for landscape connectivity. Environ. Model. Softw..

[bb0295] Saura S., Estreguil C., Mouton C., Rodríguez-Freire M. (2011). Network analysis to assess landscape connectivity trends: application to European forests (1990–2000). Ecol. Indic..

[bb0300] Saura S., Bastin L., Battistella L., Mandrici A., Dubois G. (2017). Protected areas in the world's ecoregions: how well connected are they?. Ecol. Indic..

[bb0305] UNEP-WCMC, IUCN (2016). Protected Planet Report 2016.

[bb0310] Visconti P., Elkin C. (2009). Using connectivity metrics in conservation planning: when does habitat quality matter?. Divers. Distrib..

[bb0315] Visconti P., Di Marco M., Álvarez-Romero J.G., Januchowski-Hartley S.R., Pressey R.L., Weeks R., Rondinini C. (2013). Effects of errors and gaps in spatial data sets on assessment of conservation progress. Conserv. Biol..

[bb0320] Von Haaren C., Reich M. (2006). The German way to greenways and habitat networks. Landsc. Urban Plan..

[bb0325] Wade A.A., Theobald D.M. (2010). Residential development encroachment on US protected areas. Conserv. Biol..

[bb0330] Wangchuk S. (2007). Maintaining ecological resilience by linking protected areas through biological corridors in Bhutan. Trop. Ecol..

[bb0335] Watson J.E.M., Dudley N., Segan D.B., Hockings M. (2014). The performance and potential of protected areas. Nature.

