# Supplementary material for: Protected area connectivity: Shortfalls in global targets and country-level priorities
Source: Biol Conserv. 2018 Mar;219:53–67. doi: 10.1016/j.biocon.2017.12.020 (PMC5825384; doi:10.1016/j.biocon.2017.12.020)
Supplement: Supplementary file 1 — Supplementary material [file mmc1.pdf]

Supplementary content

This file contains the supplementary content of the article:

Saura, S., Bertzky, B., Bastin, L., Battistella, L., Mandrici, A., Dubois, G. 2018. Protected area connectivity: shortfalls in global targets and country-level priorities. Biological Conservation, DOI 10.1016/j.biocon.2017.12.020.

This content is structured in the following seven appendices:

- **Appendix A**. Details on the dispersal kernel and on the importance of considering both intra-PA and inter-PA connectivity.
- **Appendix B**. Detailed methods and equations for the ProtUnconn fractions: Sea, Outland and Design.
- **Appendix C**. Details on the $ProtConn_{Bound}$ possible values and interpretation.
- **Appendix D**. Examples of countries with remarkable ProtUnconn[Sea] and ProtUnconn[Outland] values.
- **Appendix E**. Global and regional averages of the ProtConn indicators for other dispersal distances.
- **Appendix F**. Continental averages of the ProtConn indicators.
- **Appendix G**. Examples of countries illustrating the different priorities for PA connectivity.

Appendix A. Details on the dispersal kernel and on the importance of considering both intra-PA and inter-PA connectivity

A1. The negative exponential dispersal kernel

We used the negative exponential dispersal kernel to calculate the probability of direct dispersal between two PAs *i* and *j* (p_{ij}) as a function of the distance between their edges (d_{ij}), as follows:

$$p_{ij} = e^{-\alpha \cdot d_{ij}}$$

Where $1/\alpha$ is the mean dispersal distance of the considered species. The probability p_{ij} always decreases for larger distances d_{ij} (Fig. A1), although this decrease is more rapid as α increases, i.e. as the dispersal abilities of the considered species decrease (Fig. A1). When $d_{ij} = 0$ then $p_{ij} = 1$ (it is assumed that any PA can be reached from itself).

This negative dispersal kernel is widely used in assessments of the connectivity of protected areas, habitat patches or populations. This includes assessments rooted either in network analysis (e.g., Saura and Pascual-Hortal, 2007; Visconti and Elkin, 2009; Gurrutxaga et al., 2011; Pereira et al., 2011; Rodríguez-Pérez et al., 2014; Maiorano et al., 2015; Santini et al., 2016; Englehard et al., 2017) or in metapopulation models (e.g., Hanski and Ovaskainen, 2000; Moilanen and Nieminen, 2002; Visconti and Elkin, 2009). This kernel is widely used because it combines simplicity (only one parameter, α) with a reasonably good match to observed dispersal distance patterns; even if it is conservative in estimating long distance dispersal, it has been recommended when researchers and managers wish to minimize the risk of overestimating dispersal probabilities (Sutherland et al., 2000). In addition, the kernel modulates the strength of the connections between two PAs as a continuous function of the distance separating these PAs (Fig. A1).

In this study, we used the median (d_{med}), rather than the mean dispersal distance ($1/\alpha$), to characterise the dispersal range of the species considered. We did so because the median is a more appropriate measure of the central tendency of highly skewed distributions such as dispersal distance distributions. For this reason, dispersal studies most commonly report the median rather than the mean dispersal distance of a species (e.g., Sutherland et al., 2000). In any case, it is straightforward to relate the values of d_{med} and α, simply by taking natural logarithms in the equation above, and making $d_{ij} = d_{med}$ and $p_{ij} = 0.5$ (since, by definition, half of all individuals reach a distance equal to the median when dispersing), which gives:

$$\alpha = {}^{-\ln(0.5)}\!/_{d_{med}}$$

As noted in the main text (section 2.3), we considered a maximum distance of 500 km around a country's PAs to identify those transnational PAs that could contribute to upholding connectivity between the PAs of the country. We can calculate the probability p_{ij} of a dispersal event up to a distance of 500 km, here denoted as $p_{ij,500}$, depending on the median dispersal distance (d_{med}) of the species in question. To do so, we simply set d_{ij} = 500 km in the first equation in this appendix, substitute α for d_{med} using the equation above, and provide d_{med} also in kilometres, giving:

$$p_{ij,500} = e^{\frac{500 \ln(0.5)}{d_{med}}}$$

By this calculation, the probability of dispersing 500 km (see Fig. A1b) ranges from a practically negligible $p_{ij,500} = 3\times10^{-151}$ for d_{med} = 1 km up to $p_{ij,500} = 0.03125$ for the largest considered d_{med} = 100 km. For the median dispersal distance of 10 km, taken as a reference for the PA connectivity analysis and ProtConn values, $p_{ij,500}$ is as low as 9×10^{-16}. Therefore, the 500 km buffer distance (see section 2.3) is deemed sufficient to capture any likely movement through transnational PAs even for the full range of dispersal distances considered (Fig. A1b).

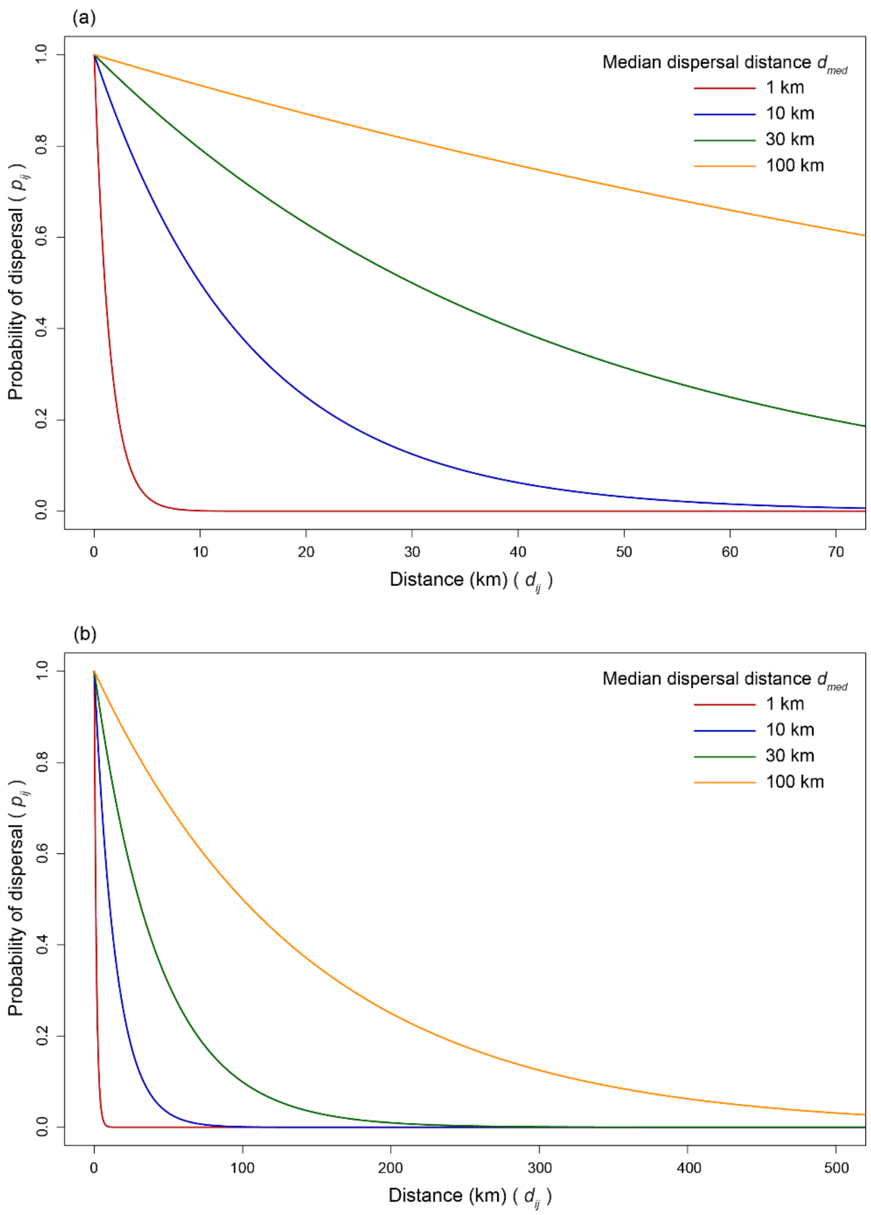

Fig. A1. The negative exponential dispersal kernel, giving the probability of direct dispersal between two PAs *i* and *j* (p_{ij}) as a function of the distance between them (d_{ij}), for the four median dispersal distances considered (1 km, 10 km, 30 km and 100 km), which encompass the dispersal abilities of the majority of terrestrial vertebrates. Panel (a) shows the negative exponential curves up to an inter-PA distance of 70 km. Panel (b) shows the same curves but up to an inter-PA distance of 500 km.

A2. The need to consider both intra-PA and inter-PA connectivity when assessing the connectivity of PA systems

Accounting for both intra-patch and inter-patch connectivity has been shown to be essential for providing meaningful connectivity metrics and assessments (Tischendorf and Fahrig, 2000; Pascual-Hortal and Saura, 2006; Saura and Pascual-Hortal, 2007; Saura, 2017; Saura and De la Fuente, 2017). Considering only inter-patch connectivity, without attention to the resources accessible within a patch, can provide misleading conservation insights when the connectivity of different patch networks, or their changes through time, are compared. For example, focusing only on inter-patch connectivity may lead to advocating the replacement of a PA by multiple smaller PAs that, while well interconnected, cover only a (small) part of the land encompassed by the original PA. This is illustrated in Figure A2 below, where cases II or I may result from downsizing or degazetting significant portions of the larger PAs in case III. A similar case would be the one in Figure A3 below, where the PA system in Y may be replaced by that in X.

To avoid these and other problems, network (graph-based) metrics that account for both intra-patch and inter-patch connectivity have been developed in recent years (Pascual-Hortal and Saura, 2006; Saura and Pascual-Hortal, 2007; Saura and Rubio, 2010; Saura and De la Fuente, 2017): the Integral Index of Connectivity (IIC), the Probability of Connectivity (PC), and the Equivalent Connected Area (ECA). These metrics underlie the ProtConn indicator used in this study, as noted in the main text.

Connectivity metrics that account for both intra-patch and inter-patch connectivity, such as the PC metric that underlies ProtConn, have been shown to outperform metrics that only consider inter-patch connectivity in their ability to explain metapopulation viability (Visconti and Elkin, 2009), species occupancy (Pereira et al., 2011) or seed deposition from the dispersal of frugivorous birds (Rodríguez-Pérez et al., 2014). An overview of these and other related studies is provided by Saura (2017).

For these reasons, recent studies have considered both intra-PA and inter-PA connectivity in the assessment of PA systems (Gurrutxaga et al., 2011; Mazaris et al., 2013; Maiorano et al., 2015; Santini et al., 2016; Saura et al., 2017). This is also the case of our study, and of the ProtConn indicator that we here use and further develop, which models and evaluates both components of connectivity. See Figures A2 and A3 below for some schematic illustrative examples.

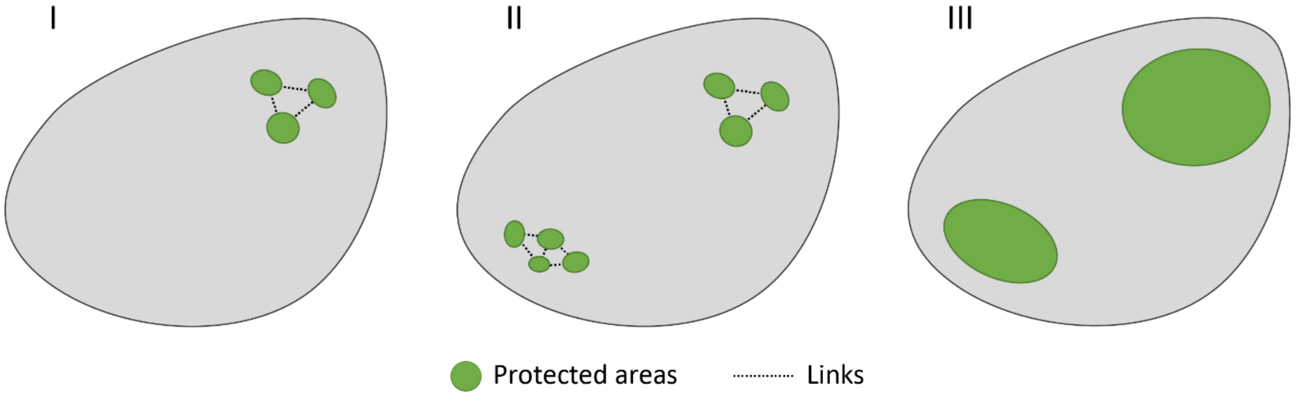

Fig. A2. Three hypothetical countries with different coverage and spatial arrangement of PAs, adapted from Saura et al. (2017). The countries are ordered by increasing ProtConn from I to III. In countries I and II there are links (connections) between some of the PAs, while in III the two PAs are isolated from each other (there is no link between them). For simplicity, in this example we assume that the probability of dispersal between two different PAs *i* and *j* is $p_{ij} = 1$ for the links shown, and $p_{ij} = 0$ otherwise. In case I, all PAs are fully connected to each other, while in II and III some PAs are isolated from some others (the PAs at opposite sides of the country). The inter-PA connectivity is hence higher in I than in II or III. However, I is the least desirable case among the three from a conservation and PA design point of view, and this is reflected by I having the lowest ProtConn value. ProtConn is higher in II than in I, because in II species can use an additional set of four connected PAs in the lower right part of the country; even though dispersal between the two groups of PAs may be rare or nonexistent, in II there are two sets of locally connected PAs while in I there is only one. Removing (degazzeting) the four PAs in the lower right part of II would result in the same PA system as in I, where inter-PA connectivity is high for all existing pairs of PAs. However, since the amount of reachable protected land is reduced by this action, it would be misleading to consider this change as beneficial for the conservation potential of the PA system. An analysis which ignores intra-patch connectivity could lead to perverse outcomes such as removing all but a small cluster of connected PAs in a country, or avoiding the designation of PAs beyond small localized clusters. On the other hand, even though in III the only two existing PAs are isolated from each other, ProtConn is higher in III than in I and II. This is because, no matter how well connected the PAs are in I and II, the amount (area) of protected land that can be reached within the two big PAs in III (intra-PA connectivity) is much larger than all the protected land that can be reached through the links between the different PAs in I and II (inter-PA connectivity). In fact, the configurations in I and II could be the result of a habitat loss and fragmentation process acting on scenario III (shrinking and degazzeting of parts of the two large PAs in III). From the standpoint of conservation, fragmentation of one large PA into several smaller PAs (even if they remain connected) should not be deemed more beneficial (or more connected) than the original continuous PA. Obviously, ProtConn would increase, both in II and III, if there was an additional link connecting the PAs in the opposite sides of the country; but even in the current setting, without such an additional link, ProtConn is highest in III and lowest in I.

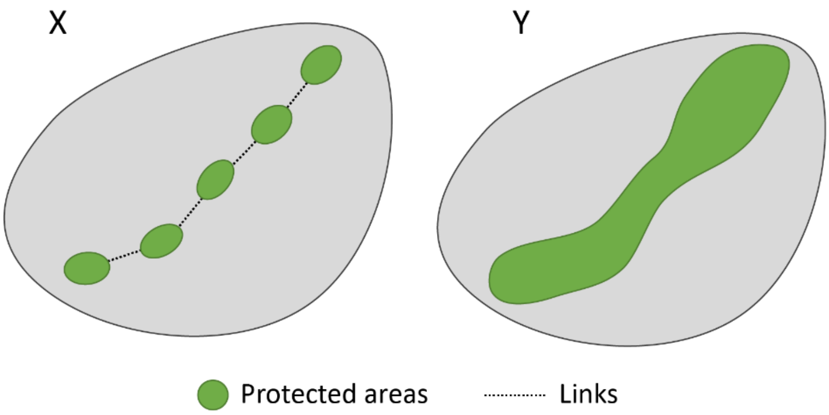

Fig. A3. Two hypothetical countries with different PA systems. In country X there is a set of PAs that are connected to each other forming a set of consecutive stepping stones, eventually allowing species to shift their ranges from one side of the country to the other. In country Y there is a single PA, hence not connected to any other PA, that would also allow the same range shifts as efficiently as, or more efficiently than, in the PA system in country X. Focusing only on inter-PA connectivity would wrongly indicate that the PA system in X is more connected than in Y, since in X every PA is connected to at least some other PA while in Y there is a single isolated PA. Focusing only on inter-PA connectivity would even indicate as desirable for connectivity to downsize and degazette the PA in Y to give several smaller PAs, linked as in X. These misleading results are avoided in the ProtConn indicator by accounting for both intra-PA and inter-PA connectivity: ProtConn is higher in Y than in X, because the intra-PA connectivity in the single PA in Y exceeds the combined inter-PA and intra-PA connectivity for all PAs in X.

References cited in Appendix A

Engelhard, S.L., Huijbers, C.M., Stewart-Koster, B., Olds, A.D., Schlacher, T.A., Connolly, R.M., 2017. Prioritising seascape connectivity in conservation using network analysis. J. Appl. Ecol. 54, 1130–1141.

Gurrutxaga, M., Rubio, L., Saura S., 2011. Key connectors in protected area networks and the impact of highways: a transnational case study from the Cantabrian Range to the Western Alps. Landsc. Urban Plan. 101, 310–320.

Hanski, I., Ovaskainen, O., 2000. The metapopulation capacity of a fragmented landscape. Nature 404, 755–758.

Maiorano, L., Amori, G., Montemaggiori, A., Rondinini, C., Santini, L., Saura, S., Boitani, L., 2015. On how much biodiversity is covered in Europe by national protected areas and by the Natura 2000 network: insights from terrestrial vertebrates. Conserv. Biol. 29, 986–995.

Mazaris, A.D., Papanikolaou, A.D., Barbet-Massin, M., Kallimanis, A.S., Jiguet, F., Schmeller, D.S., Pantis, J.D., 2013. Evaluating the connectivity of a protected areas' network under the prism of global change: the efficiency of the European Natura 2000 network for four birds of prey. PLoS ONE 8, e59640.

Moilanen, A., Nieminen, M., 2002. Simple connectivity measures in spatial ecology. Ecology 83, 1131–1145.

Pascual-Hortal, L., Saura, S., 2006. Comparison and development of new graph-based landscape connectivity indices: towards the priorization of habitat patches and corridors for conservation. Landsc. Ecol. 21, 959–967.

Pereira, M., Segurado, P., Neves, N., 2011. Using spatial network structure in landscape management and planning: a case study with pond turtles. Landsc. Urban Plan. 100, 67–76.

Rodríguez-Pérez, J., García, D., Martínez, D., 2014. Spatial networks of fleshy-fruited trees drive the flow of avian seed dispersal through a landscape. Funct. Ecol. 28, 990–998.

Santini, L., Saura, S., Rondinini, C., 2016. Connectivity of the global network of protected areas. Divers. Distrib. 22, 199–211.

Saura, S., Pascual-Hortal, L., 2007. A new habitat availability index to integrate connectivity in landscape conservation planning: comparison with existing indices and application to a case study. Landsc. Urban Plan. 83, 91–103.

Saura, S., Rubio, L., 2010. A common currency for the different ways in which patches and links can contribute to habitat availability and connectivity in the landscape. Ecography 33, 523–537.

Saura, S., 2017. Node self-connections in network metrics. Ecology Letters, in press.

Saura, S., De la Fuente, B., 2017. Connectivity as the amount of reachable habitat: conservation priorities and the roles of habitat patches in landscape networks. In: Gergel, S.E., Turner, M.G. (Eds.), Learning Landscape Ecology, 2nd edition, pp. 229-254. Springer-Verlag, New York. http://dx.doi.org/10.1007/978-1-4939-6374-4_14.

Saura, S., Bastin, L., Battistella, L., Mandrici, A., Dubois, G., 2017. Protected areas in the world's ecoregions: How well connected are they? Ecol. Indic. 76, 144–158.

Sutherland, G. D., Harestad, A. S., Price, K., Lertzman, K. P., 2000. Scaling of natal dispersal distances in terrestrial birds and mammals. Conserv. Ecol. 4(1), 16.

Tischendorf, L., Fahrig, L., 2000. How should we measure landscape connectivity? Landsc. Ecol. 15, 633–641.

Visconti, P., Elkin, C., 2009. Using connectivity metrics in conservation planning: when does habitat quality matter? Divers. Distrib. 15, 602–612.

Appendix B. Details on the methods and equations for the ProtUnconn fractions: Sea, Outland and Design

B1. The ProtUnconn[Sea] fraction

ProtUnconn[Sea] quantifies the natural isolation of terrestrial PAs by the sea and is calculated as:

$$\text{ProtUnconn[Sea]} = 100 \cdot \frac{\sum_{i=1}^{n} a_i}{A_L} - 100 \cdot \frac{\sqrt{\sum_{i=1}^{n+t+q+r} \sum_{j=1}^{n+t+q+r} a_i a_j \, p_{ij}^*}}{A_L}$$

where *q* is the number of land portions in the country (i.e. number of non-contiguous land polygons for the country), *r* is the number of land portions of other countries that fall entirely or partially within a transboundary buffer (here of 500 km) outside the PAs of the country[1], and everything else is as described for ProtConn in section 2.4 of the main text. The calculation of ProtUnconn[Sea] uses a network in which the PAs within the country (*n*) have an attribute equal to their area, and all other polygons (*t*+*q*+*r*) are also included but with an attribute equal to zero[2]. All land portions, either of the country (*q*) or of other countries (*r*), have been here obtained from the land polygons as mapped in GAUL 2015.

The difference between the equation for ProtConn (section 2.4 of the main text) and the right term of the equation for ProtUnconn[Sea] consists in that, when calculating ProtUnconn[Sea], the country and foreign land portions (*q*+*r*) are included in the network (as polygons with an attribute equal to zero). These zero-attribute polygons will contribute only as connectors between the polygons with non-zero attributes (the PAs belonging to the country), as given by the connector fraction of the PC metric (Saura and Rubio, 2010) that underlies ProtConn.

Given that the distances between the polygons are calculated as edge-to-edge distances (see section 2.3 in the main text), in ProtUnconn[Sea] the probability of movement between two PAs *i* and *j* (p^*_{ij}) will only be $p^*_{ij} < 1$ when movement needs to happen, even if only partially, outside the set of considered polygons (*n*+*t*+*q*+*r*) to be able to reach one PA from the other. Since any terrestrial PA of a country will overlap at least one of the land portions of the country, the only case in this network setting in which two PAs can have $p^*_{ij} < 1$ is when the two PAs are separated by the sea (i.e. located in different islands or land masses). If it is possible to move between any pair of terrestrial PAs in the country without traversing the sea, then all pairs of PAs in the country will have $p^*_{ij} = 1$ and $\sqrt{\sum_{i=1}^{n+t+q+r} \sum_{j=1}^{n+t+q+r} a_i a_j \, p_{ij}^*}$ will be equal to $\sum_{i=1}^{n} a_i$, giving ProtUnconn[Sea] = 0 (no isolation of PAs by the sea as considered by ProtUnconn[Sea]). This includes the

---

[1] Disputed territories (i.e. lands with sovereignty disputed by two or more countries) as mapped in GAUL 2015 are included as part of *q* in the calculations when the country has declared to the WDPA one or several PAs falling (partially or entirely) within such disputed territory, hence accepting, for the purposes of this assessment, what the countries submit to the WDPA. If two different countries claim, through the information they submit to the WDPA, to have PAs under their jurisdiction in the same disputed territory, such disputed territory is included as part of *q* for both countries. Any disputed territory that has no PAs declared by the country (as given by the WDPA) is considered as part of *r* for that country (as long as such territory falls, partially or entirely, within the 500 km buffer around the PAs of the country). This is the procedure applied for dealing with disputed territories in the calculation of the three ProtUnconn fractions, and hence also of $\text{ProtConn}_{\text{Bound}}$. This analytical procedure by no means implies any endorsement or recognition by the authors, nor any official position by the European Commission, on the sovereignty of any disputed territory.

[2] In theory, all transnational PAs outside the country (*t*) should be located within a foreign land portion (*r*). Therefore, all the land covered by the *t* polygons would be already covered by the *r* polygons, making it unnecessary to include *t* if *r* is already included in the analysis, given that both types of polygons are treated in the same way in the network (i.e. as nodes with attribute equal to zero). However, in a few cases there might be small mismatches between the boundaries of a PA submitted by a country to the WDPA (using their own maps and datasets) and the boundaries of the country as mapped in a worldwide country layer such as GAUL. For this reason, it is advisable to include both *t* and *r* to avoid the effect of any potential (even if small) mismatches between the layers, and hence to provide a fully consistent correction and calculation across the different networks and set of polygons used to obtain the three ProtUnconn fractions.

case in which the movement between two PAs in the same island or landmass would need to traverse larger distances through land than the distance of a movement directly across the sea, such as two PAs in opposite sides of a fjord or other similar situations, all of which will give ProtUnconn[Sea] = 0.

ProtUnconn[Sea] will be equal to zero in three cases:

- Obviously, when $q = 1$ (the country is constituted of a single continuous land portion).
- When $q > 1$ but the land portions are separated only by other countries and not by the sea.
- When $q > 1$ and some of the country's land portions are separated by the sea, but all the PAs of the country are located in just one of the land portions, or they are located in portions separated by other countries but not by the sea. If all the PAs of the country fall within the same land portion, the isolation that they may have is not due to the sea but to the lack of an adequate design of the PA system within that land portion. If all the PAs of the country fall within different land portions that are separated by other countries and not by the sea, the isolation that they may have is not due to the sea but to foreign lands and/or to the lack of an adequate design of the PA system within the land portions of the country.

When in the country there are land portions ($q > 1$) with PAs separated by the sea (i.e., excluding the three abovementioned cases) then ProtUnconn[Sea] > 0. This is the case of countries distributed across several islands, like the Philippines or Antigua and Barbuda, or of countries distributed between island and mainland territories, like Greece or Italy; see the detailed values of ProtUnconn[Sea] and of other ProtConn-related indicators for some of these countries in Appendix D.

In a country in which the sea separates its territory into multiple land portions ($q > 1$), the value of ProtUnconn[Sea] will increase in response to:

- Increasing number of land portions (q) that, having PAs, are separated by the sea.
- Increasing evenness with which the area covered by PAs in the country is distributed among the different land portions. For example, a tiny PA in a small island belonging to a country with nearly all its land area and PA coverage in a large continuous mainland will make ProtUnconn[Sea] only very slightly (perhaps unnoticeably) larger than zero.
- Increasing distances through the sea that separate the different land portions with PAs, combined with smaller species dispersal distance. This is because the combination of the distance between the land portions and the species dispersal abilities will determine the actual probability of movement (p^*_{ij}) between the land portions separated by the sea.

Finally, it may be noted that also inland waters (mainly big lakes), and not just the sea, can contribute to separate the territory of a country into disjoint land portions (inland islands). In practice, however, this only happens in a very few cases that have little importance for the national and global assessment conducted here; for example, the islands in the Great Lakes (North America), such as Isle Royale (USA) in Lake Superior, or Amherst Island (Canada) in Lake Ontario. These inland islands are also accounted for in the calculation of ProtUnconn[Sea] and of the rest of the connectivity indicators, and are treated in the same way as the sea-separated land portions. These inland islands are, however, very few compared to the sea-separated islands, very small compared to the size of other land portions (either the mainland or the sea-separated islands), and located at much smaller distances from the mainland than the land portions separated by the sea. Therefore, they contribute very little to the isolation of PAs at the country level. They have a largely unnoticeable effect in the final value of ProtUnconn[Sea], as well as of the rest of the PA connectivity indicators, and hence we refer to this fraction simply as the "Sea" fraction. For this reason, we also frame all the description and discussion on this fraction (and the related ones) in the context of the PA separation by the sea.

B2. The ProtUnconn[Outland] fraction

ProtUnconn[Outland] quantifies the isolation of the terrestrial PAs of the country due to unprotected foreign lands, and is calculated as:

$$\mathrm{ProtUnconn[Outland]} = 100 \cdot \frac{\sqrt{\sum_{i=1}^{n+t+q+r} \sum_{j=1}^{n+t+q+r} a_i a_j \, p_{ij}^*}}{A_L} - 100 \cdot \frac{\sqrt{\sum_{i=1}^{n+t+q} \sum_{j=1}^{n+t+q} a_i a_j \, p_{ij}^*}}{A_L}$$

where all the variables are as described above.

According to this equation, ProtUnconn[Outland] is given by the difference between the following two terms (the two numerators in the equation above) that are calculated using two different networks:

i) The first network (*n+t+q+r*, to the left in the previous equation) corresponds to that already described above for ProtUnconn[Sea], i.e. a network in which the PAs within the country (*n*) have an attribute equal to their area and all other polygons (*t+q+r*) are also included but with an attribute equal to zero. It holds that:

   - This term *n+t+q+r* will only be lower than $\sum_{i=1}^{n} a_i$ because of the separation of the country land portions by the sea, as described in the previous section B1 for ProtUnconn[Sea].

ii) The second network (*n+t+q*, to the right in the equation above) corresponds to a network in which the PAs within the country (*n*) have an attribute equal to their area, the transboundary PAs (*t*) are included with an attribute equal to zero, the land portions of the country (*q*) are included with an attribute equal to zero, and the land portions of other countries within the transnational buffer (*r*) are not included. It holds that:

   - The term *n+t+q+r* can never be lower than the term *n+t+q*, since the latter includes fewer nodes (polygons) and fewer potential connections, all of which are accounted for in the former.
   - The term *n+t+q* will be lower than $\sum_{i=1}^{n} a_i$ because of the separation by both sea and foreign lands. Given that the previous term *n+t+q+r* will only be lower than $\sum_{i=1}^{n} a_i$ because of the separation by the sea, the difference between these two terms will determine the isolation of PAs caused by foreign lands, i.e. ProtUnconn[Outland].

The term *n+t+q+r* will only be larger than the term *n+t+q*, and hence ProtUnconn[Outland] will only be larger than zero, when the following three conditions (c1, c2 and c3) are all met:

c1) First, that there is at least one foreign land portion that separates the country land into several non-contiguous portions. If the transnational buffer (here 500 km) includes several foreign land portions *r* but these foreign portions do not dissect the country land portions (i.e. it is possible to move between all the PAs of the country without traversing foreign lands), then ProtUnconn[Outland] = 0. Obviously, if *q* = 1 or if *q* > 1 but all the country land portions are separated by the sea and not by foreign lands, then ProtUnconn[Outland] = 0.

c2) Second, that there is no protection (no transboundary PAs) in at least part of the foreign land that needs to be traversed to move between the country land portions. In other words, this second condition will not be met if the PAs in the foreign land portion are set up in a way that provides full continuity of protected land via such movement through foreign lands.

c3) Third, there are PAs of the country in at least two of the country land portions that are separated by the foreign lands. If all the PAs of the country fall within the same land portion, the isolation that they may have is just due to the lack of an adequate design of the PA system within that land portion. If all the PAs of the country are distributed across several land portions, but still there are no PAs in

the country land portions that are separated from the rest of the nation by foreign lands, the isolation that PAs may have is not due to foreign lands but due to the sea or to the lack of an adequate design of the PA system within the land portions of the country.

These three conditions are met for example in the USA, where Alaska is separated by Canada from the conterminous states, or in Brunei, separated into two parts by the state of Sarawak in Malaysia. A special case of condition c1 above is the following: two (or more) land portions of a country are separated by the sea (for example the Greek island of Lesvos and mainland Greece) but in between these portions there is land of another country located in such a way that it could be used as stepping-stone land when moving between the country portions to reduce the distance that needs to be traversed through the sea (e.g. using Turkish land in the movement between the island of Lesvos and mainland Greece). In this example, Greece will have ProtUnconn[Outland] > 0 as long as condition c2 above is also met for the land and PAs in Turkey and condition c3 is met as well (that PAs are declared both in Lesvos and mainland Greece). All these conditions are actually met for the case of Greece, which gives ProtUnconn[Outland] > 0 (Appendix D) due not only to Lesvos but also to a few other Greek islands in a similar case. See the detailed values of ProtUnconn[Outland] and of other ProtConn-related indicators for some countries like Brunei or Greece in Appendix D.

Finally, and even if it is implicitly given by the conditions above, it is worth noting that the potential lack of transboundary PAs in the foreign country lands (*r*) will not always translate into ProtUnconn[Outland] > 0. This will only happen if the movement between the PAs of the country could not be completed by using only protected lands within the country once the country had already designated all the necessary PAs within its land to try to make such movement possible. Therefore, ProtUnconn[Outland] will be larger than zero only if the transboundary PAs would continue to be necessary to connect two PAs of a country even in the theoretical case of the whole country being covered by PAs. In other words, the responsibility of PA isolation is only assigned to other countries (ProtUnconn[Outland]) when the considered country alone has no possibility (even if theoretical) of providing a continuous protected pathway for movement between its PAs, i.e. when that would not be possible even if the country had declared all its land as protected.

When all the above mentioned conditions (c1, c2 and c3) are met, ProtUnconn[Outland] for a country will increase in response to:

- Increasing number of land portions (*q*) that, having PAs, are separated by unprotected foreign lands.
- Greater evenness with which the area covered by PAs in the country is distributed among these different land portions separated by unprotected foreign lands.
- Larger distances through unprotected foreign lands that need to be traversed to move between the country's different land portions with PAs, particularly with smaller species dispersal distance. This is because the combination of the distance to be traversed through unprotected foreign lands when moving between the country land portions and the species dispersal abilities will determine the actual probability of movement (p^*_{ij}) between the land portions separated by unprotected foreign lands.

B3. The ProtUnconn[Design] fraction

ProtUnconn[Design] quantifies the isolation of PAs which stems from limitations in the design of the terrestrial PA system of the country (this is the part of the PA isolation for which a country can be made accountable), and is calculated as:

$$\text{ProtUnconn[Design]} = 100 \cdot \frac{\sqrt{\sum_{i=1}^{n+t+q} \sum_{j=1}^{n+t+q} a_i a_j \, p_{ij}^*}}{A_L} - 100 \cdot \frac{\sqrt{\sum_{i=1}^{n+t} \sum_{j=1}^{n+t} a_i a_j \, p_{ij}^*}}{A_L}$$

where all terms are as described above. In this equation, ProtUnconn[Design] is obtained from the difference between two terms (the two numerators in this equation) that are calculated using two different networks. The first network (n+t+q, to the left in the equation above) considers all PAs within the country (n) with an attribute equal to their land area, all the transboundary PAs (t) with an attribute equal to zero, and all the the land portions of the country (q) with an attribute equal to zero. The second network (n+t, to the right in the equation above) only considers the PAs: the PAs within the country (n) with an attribute equal to their area, and the transboundary PAs (t) with an attribute equal to zero. The term n+t+q can never be lower than the term n+t, since the latter includes fewer nodes (polygons) and fewer potential connections, all of which are accounted for in the former. The difference between the two terms will be different from zero, and hence ProtUnconn[Design] > 0, when the movement between the PAs of the country requires traversing some unprotected lands within the country, i.e. when there is a lack of connectivity and continuity in the PAs within the country. In other words, ProtUnconn[Design] > 0 when the connectivity between the PAs of the country would increase if other currently unprotected lands of the country were designated as PAs serving as corridors between the rest of the PAs in the country.

Appendix C. Details on the $\text{ProtConn}_{\text{Bound}}$ possible values and interpretation

C1. When will $\text{ProtConn}_{\text{Bound}}$ be equal to ProtConn and/or Prot?

$\text{ProtConn}_{\text{Bound}}$ is the version of the Protected Connected indicator adjusted to account only for the part of PA connectivity that it is within the power of the country to influence (excluding the effect of PA isolation caused by the sea and by unprotected foreign lands). As noted in the main text, $\text{ProtConn}_{\text{Bound}}$ is calculated as:

$$\text{ProtConn}_{\text{Bound}} = \text{Prot} - \text{ProtUnconn[Design]}$$

Which is the same as calculating $\text{ProtConn}_{\text{Bound}}$ as:

$$\text{ProtConn}_{\text{Bound}} = \text{ProtConn} + \text{ProtUnconn[Sea]} + \text{ProtUnconn[Outland]}$$

Since, as already noted in the main text, ProtUnconn[Design] ≤ ProtUnconn, then $\text{ProtConn}_{\text{Bound}} \geq$ ProtConn. On the other hand, since ProtUnconn[Design] ≥ 0, then $\text{ProtConn}_{\text{Bound}} \leq$ Prot. In words, $\text{ProtConn}_{\text{Bound}}$ will never be higher than the PA coverage, and will never be smaller than the non-adjusted ProtConn. In particular, the following three cases hold regarding the values of $\text{ProtConn}_{\text{Bound}}$, ProtConn and Prot:

- $\text{ProtConn}_{\text{Bound}}$ will only be equal to the PA coverage (Prot) when, given any two point locations selected within the PAs of the country, the movement between these locations can be entirely accomplished by traversing either protected lands, the sea, or unprotected lands in other countries, but with no need to traverse any unprotected lands within the country.

- $\text{ProtConn}_{\text{Bound}}$ will only be equal to both Prot and ProtConn when, given any two locations selected within the PAs of the country, the movement between these locations can be entirely accomplished by traversing protected lands only, with no need to traverse the sea nor any unprotected lands (within or outside the country).

- $\text{ProtConn}_{\text{Bound}}$ will only be smaller than Prot but equal to ProtConn when, given any two locations selected within the PAs of the country, it is necessary that part of the movement between at least two of these locations traverses some unprotected land within the country but there is no need to traverse the sea or unprotected lands in other countries.

C2. ProtConn penalizes the PA connectivity scores for countries with several non-contiguous land portions

As given by the equation for ProtConn in section 2.4 in the main text, ProtConn will only be equal to Prot, and ProtUnconn will only be equal to zero, when the numerator of ProtConn, i.e. the Equivalent Connected Area (ECA) (Saura et al., 2011; Saura and de la Fuente 2017), equals the total area of land protected in the country ($\sum_{i=1}^{n} a_i$). Whether this will actually happen or not depends on the country's efforts to design a well-connected terrestrial PA system, but not solely on these efforts. It will also depend, as noted in Appendix B, on whether the country land is separated into several non-contiguous land portions (q) which have PAs. The following cases can be differentiated:

I. A country with its land distributed in a single continuous landmass (q = 1). In this case, ProtUnconn[Sea] = 0, ProtUnconn[Outland] = 0, ProtConn = $\text{ProtConn}_{\text{Bound}}$, and the country may, in theory, achieve ProtUnconn = 0 and ProtConn = Prot, or get as close as desired to that situation, through an adequate design and reinforcement of its terrestrial PA system.

II. A country in which the sea separates its territory into multiple land portions (q > 1), each containing some of the PAs of the country. This is the case of countries distributed across several islands, like the Philippines, or distributed between island and mainland territories, like Greece. In this case, because of the isolation imposed by the sea, ProtUnconn ≥ ProtUnconn[Sea] > 0, ProtConn < $\text{ProtConn}_{\text{Bound}}$, ECA will never be as high as $\sum_{i=1}^{n} a_i$, and ProtConn < Prot, no matter how well the terrestrial PA system in the country is designed. The detailed values for the example of Greece are shown in Appendix D.

III. A country in which its territory is separated in several non-continuous portions because of the land of other nations being located in between ($q > 1$), in such a way that any terrestrial movement between at least one pair of PAs of the country needs to traverse some distance across foreign lands. This is the case for example of the USA, where Alaska is separated by Canada from the conterminous states, or Brunei, separated into two parts by the state of Sarawak in Malaysia (see the detailed values of the ProtConn-related indicators for the example of Brunei in Appendix D). Two subcases can be differentiated here, the first being the more common of the two:

IIIa. The distance that needs to be traversed across foreign lands is, partially or fully, unprotected. In this subcase, ProtUnconn ≥ ProtUnconn[Outland] > 0, ProtConn < $\text{ProtConn}_{\text{Bound}}$, and ProtConn will decrease (will be lower than Prot) for this reason. In other words, when the lack of PAs designated by a different country imposes some distance to be traversed through unprotected lands in the movement between the PAs of the country under consideration, this will impose some decrease in the value of ProtConn. This decrease will be for a reason (ProtUnconn[Outland]) that is not under the control or jurisdiction of the country whose PAs are being considered. This is the subcase that applies for the examples of USA and Brunei.

IIIb. If there is a pathway that can be followed to traverse the foreign lands entirely through PAs designated by the other country, there is no PA isolation that can be attributed to a different country. In this subcase, ProtUnconn may or may not be larger than zero, but even if it was larger than zero, and assuming that there is no isolation by the sea, it would be entirely because of limitations in the design of the PA system in the considered country, with no responsibility falling on other countries. In other words, in this subcase there would be no decrease in the ProtConn value for the country that could be attributed to the lack of PAs in other countries (ProtUnconn[Outland] = 0, ProtConn = $\text{ProtConn}_{\text{Bound}}$).

IV. Finally, it is obviously possible that there are several non-contiguous land portions in a country ($q > 1$) as a combined result of the two previous circumstances II and IIIa (separation both by the sea *and* by unprotected foreign lands). This is the case, for example, for the USA, where Alaska is separated by Canada from the conterminous USA but where the country's land is also partially distributed across islands like Hawaii. In this case, ProtUnconn[Sea] > 0, ProtUnconn[Outland] > 0, ProtConn < $\text{ProtConn}_{\text{Bound}}$ and ProtConn will obviously also be lower than Prot, no matter how well the terrestrial PA system in the country is designed.

To give an illustrative example, a country may have achieved a given PA coverage (Prot) well above the 17% level of Aichi Target 11, and may have designed the PA system very well regarding connectivity, but may find that its ProtConn score falls below the 17% of Aichi Target 11, simply because of factors outside its control (cases II and IIIa, or their combination in case IV). This is for example the case of Greece (Fig. D1 in Appendix D). Similarly, there is a theoretical extreme but interesting example to consider: the case in which all the land of the country is covered by PAs, i.e. Prot = 100%. This case would certainly be one in which the country has done all that is in its power to reinforce the terrestrial PA system; there is nothing else that the country can do, regarding the design of the PA system, to promote the continuity and connectivity of PAs in the country. Although in such a case one might expect that ProtConn would reach its maximum possible value (ProtConn = 100%), and that ProtUnconn would be 0%, the effect of isolation by the sea and/or foreign unprotected lands (cases II, IIIa and IV above) can still make ProtConn fall below 100%. Such a shortfall would happen for reasons that are outside the control of the country, and for which, therefore, it would not be fair to penalize the country.

$\text{ProtConn}_{\text{Bound}}$ avoids this penalization of the ProtConn values for factors outside a country's responsibility, by factoring out two of the three ProtUnconn fractions: ProtUnconn[Sea], as related to case II, and ProtUnconn[Outland], as related to case IIIa above. In the example of Greece, $\text{ProtConn}_{\text{Bound}}$ is above 17% even if ProtConn falls below that level (Fig. D1 in Appendix D). In the hypothetical example above of a country with all the land covered by PAs (Prot = 100%), $\text{ProtConn}_{\text{Bound}}$ will also reach 100% even if ProtConn does not.

While ProtConn will never decrease for larger dispersal distances (d_{med}), this can happen for $\text{ProtConn}_{\text{Bound}}$ because of the indicator adjustment to account only for the part of PA connectivity that it is within the power of the country to influence (excluding the isolation due to the sea and foreign lands). Consider, for example, a case of a country with (i) two islands separated by the sea, (ii) one PA in the center of each of the islands, and (iii) the PAs at some distance from the shore (i.e. a movement from the edge of the PA to the shore would need to traverse some unprotected lands). When moving between these PAs, the distance to be traversed through the sea, alone, may result in a low connectivity for a species with limited dispersal abilities. In the extreme, movement between the two PAs on the different islands would be impossible for such species just because of this sea distance, yielding $p^*_{ij} = 0$ in the left term of the ProtUnconn[Design] equation (section B3 in Appendix B). In such case, if we additionally include the distance through unprotected land (from the edge of the PA to the shore, in addition to the sea distance) p^*_{ij} will be also equal to zero in the right term of the ProtUnconn[Design] equation (since p^*_{ij} can never increase for larger distances to be traversed), giving ProtUnconn[Design] = 0 in this case. On the contrary, a species with larger dispersal abilities may be able to cover the sea distance with a relatively high or at least significant p^*_{ij} (left term in the equation for ProtUnconn[Design]). However, if the effect of the unprotected PA-shore distance is included, the increase in the total distance may result, even for this more mobile species, in a lower p^*_{ij} in the right term of the ProtUnconn[Design] equation. This would give ProtUnconn[Design] > 0, as obtained from the difference in the values of the two terms in the ProtUnconn[Design] equation (section B3 in Appendix B) for this mobile species. Situations like this one would hence translate into a lower $\text{ProtConn}_{\text{Bound}}$ value for the species with larger dispersal distance d_{med}, since $\text{ProtConn}_{\text{Bound}}$ is the difference between PA coverage (Prot, which does not vary with d_{med}) and ProtUnconn[Design]. In our actual results for national PA systems, however, the large majority of $\text{ProtConn}_{\text{Bound}}$ values increased with dispersal distance. Only in about 7% of the cases (countries and dispersal distances considered in this study), $\text{ProtConn}_{\text{Bound}}$ decreased to some extent when the considered species median dispersal distance d_{med} increased (from 1 to 10 km, from 10 to 30 km, or from 30 to 100 km). A decrease in $\text{ProtConn}_{\text{Bound}}$ above 0.1% for a larger d_{med} only happened in 3% of the cases, and a decrease in $\text{ProtConn}_{\text{Bound}}$ above 1% for a larger d_{med} only happened in 0.4% of the cases. These cases all correspond to countries with multiple islands of relatively comparable sizes, and with at least some PAs that do not cover the entire island, so that this kind of effect (PA-shore vs. sea distance) is prominent enough in the final indicator values. This is the case, for example, for the Philippines, New Zealand or Antigua and Barbuda.

C3. ProtConn and $\text{ProtConn}_{\text{Bound}}$: when to use which one?

The Protected Connected indicator is, therefore, available in two different versions: the non-adjusted ProtConn, as presented in Saura et al. (2017), and $\text{ProtConn}_{\text{Bound}}$ as presented in this work. It is important to note that none of these two versions can be regarded in general as superior to the other, since this will depend on the objective of the assessment. The question therefore is: which of the two indicator versions, ProtConn and $\text{ProtConn}_{\text{Bound}}$, is to be used for what?

If, for example, we are interested in the ecological effects of the actual levels of PA isolation, then the indicator to use is ProtConn, since it captures together all the factors that drive the potential lack of PA connectivity. If, for instance, gene flow is expected to decrease in PA systems with low connectivity (all other factors being equal), this would be better assessed with ProtConn than with $\text{ProtConn}_{\text{Bound}}$. For example, the gene flow between Alaska and the state of Washington in the USA, or between Hawaii and Alaska, may be low. This would be accounted for in ProtConn, but not in $\text{ProtConn}_{\text{Bound}}$, since the latter discounts the PA isolation caused by the sea or foreign lands, which is not in the power of a country to influence but will indeed have some effect on gene flow or on other ecological patterns and processes that may be considered.

If, however, we are interested in specifically evaluating the efforts of a country towards connectivity targets (such as Aichi Target 11), and in providing a fair comparison of those efforts with those made in other countries, then $\text{ProtConn}_{\text{Bound}}$ is the indicator to use, as we do in this work. This is because $\text{ProtConn}_{\text{Bound}}$ only considers the part of PA isolation that is in the power of the country to influence, while ProtConn would penalize the country for reasons that are out of its control (isolation because of the sea and because of other

countries). Here the interest is not to evaluate the ecological consequences of PA connectivity/isolation, but to provide a more conservation policy oriented evaluation of how much has been done, compared to how much can be actually done, in designing a well-connected system of PAs in a country (as given by what is really feasible to achieve within the possibilities of the country). Therefore, $ProtConn_{Bound}$ is more appropriate for a policy-relevant assessment such as the one here provided for the world's countries.

Appendix D. Examples of countries with remarkable ProtUnconn[Sea] and ProtUnconn[Outland] values

The ProtUnconn[Sea] and ProtUnconn[Outland] values were very unevenly distributed among the countries, particularly for ProtUnconn[Outland]. For d_{med} = 10 km, 69% of the countries had zero or very small ProtUnconn[Sea] fraction (< 0.1%), and 97% had zero or very small ProtUnconn[Outland] fraction (< 0.1%). The global ProtUnconn[Outland] of 0.2% for d_{med} = 10 km (Fig. 1 in the main text) was largely determined by the case of the USA, in which ProtUnconn[Outland] = 3.6%. Apart from the USA, there were only 7 other countries or territories with ProtUnconn[Outland] above 0.1%: Greece (Fig. D1), Croatia, Brunei Darussalam (Fig. D2), Timor-Leste, Equatorial Guinea, Croatia, Belize, and the small island group Bonaire, Sint Eustatius and Saba (three special municipalities of the Netherlands located in the Caribbean Sea).

Five countries meet the 17% target as measured by $ProtConn_{Bound}$ but not as measured by ProtConn, i.e. they only meet the target when the effects of ProtUnconn[Sea] and ProtUnconn[Outland] have been factored out in the assessment. These five countries are Greece (Fig. D1), Denmark, Antigua and Barbuda (Fig. D3), Equatorial Guinea, and Kiribati. Other countries were above the 17% target even before accounting for ProtUnconn[Sea] and ProtUnconn[Outland], i.e. as given by ProtConn, such as Brunei Darussalam (Fig. D2), while others remained below the 17% $ProtConn_{Bound}$ target even when $ProtConn_{Bound}$ was significantly higher than ProtConn, as is the case for example for Italy (Fig. D4).

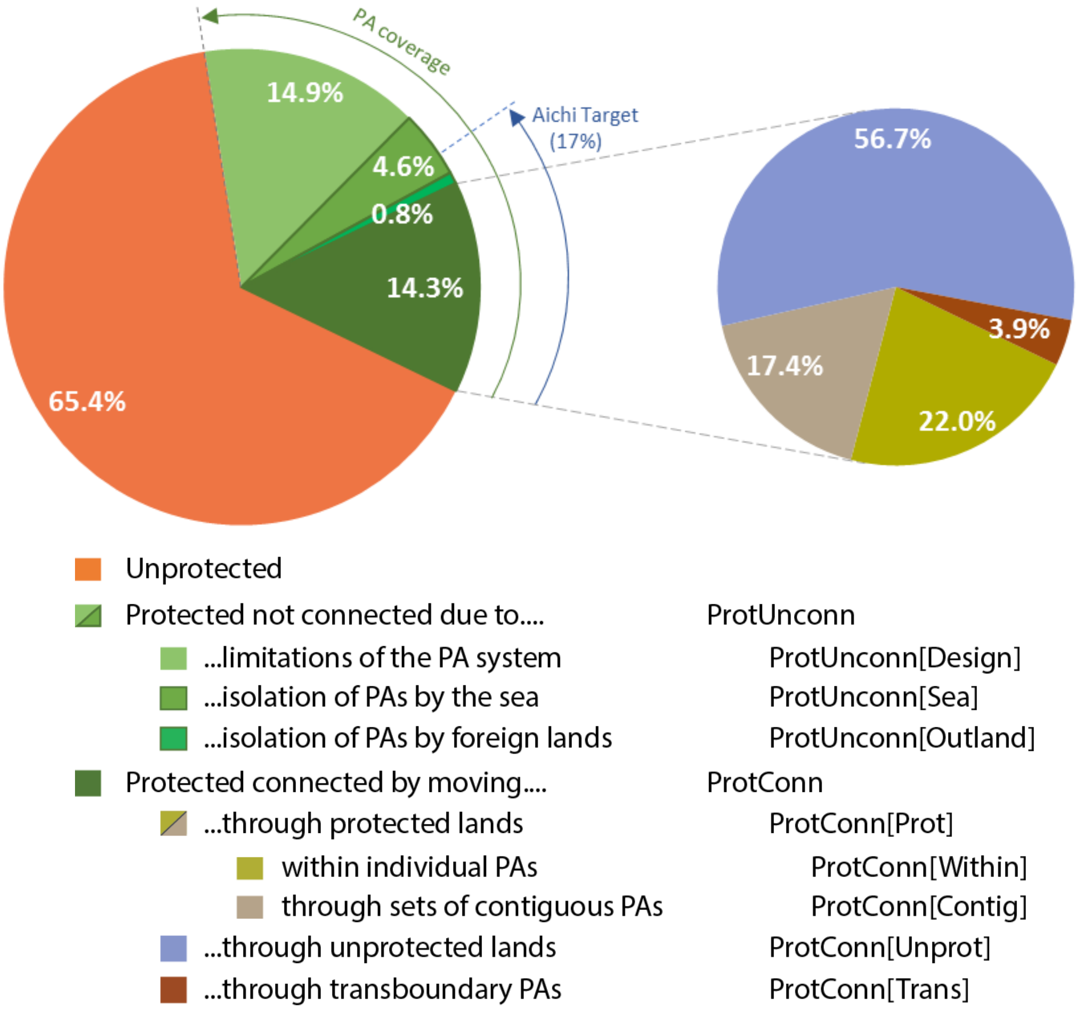

Fig. D1. Example for Greece with the ProtConn-related indicators calculated for d_{med} = 10 km. While the non-adjusted ProtConn for Greece is 14.3%, i.e. below the 17% target, $ProtConn_{Bound}$ equals 19.7%, i.e. clearly above the 17% target. PA coverage is 34.6% (Prot = 34.6% = 100% - 65.4% = 14.9% + 0.8% + 4.6% + 14.3%). The 19.7% value for $ProtConn_{Bound}$ is given by $ProtConn_{Bound}$ = Prot - ProtUnconn[Design] = 34.6% - 14.9% or by $ProtConn_{Bound}$ = ProtConn + ProtUnconn[Sea] + ProtUnconn[Outland] = 14.3% + 4.6% + 0.8%.

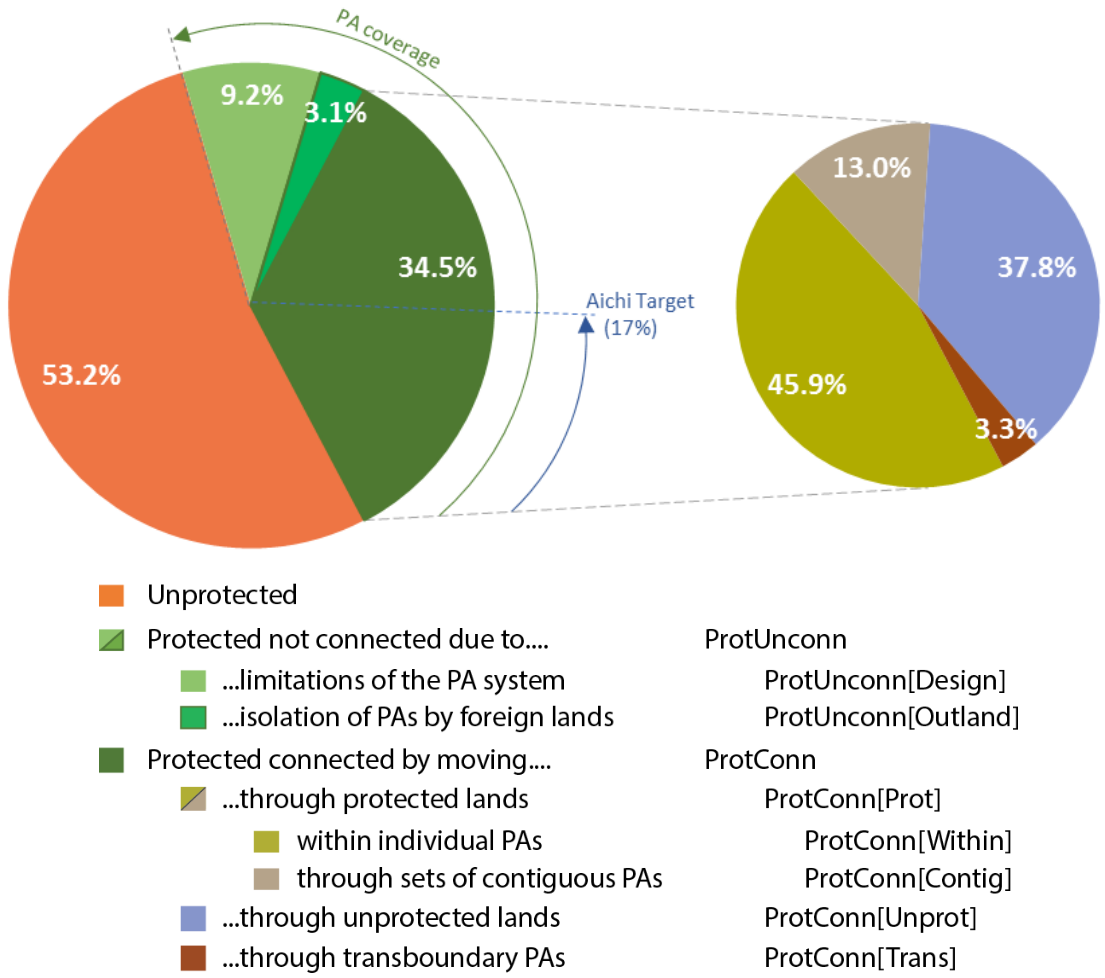

Fig. D2. Example for Brunei with the ProtConn-related indicators calculated for d_{med} = 10 km. The non-adjusted ProtConn for Brunei is 34.5%, and $\text{ProtConn}_{\text{Bound}}$ equals 37.6%, i.e. clearly above the 17% target. In Brunei, ProtUnconn[Sea] is 0% and hence it is not shown in this figure. PA coverage is 46.8% (Prot = 100% - 53.2% = 34.5% + 3.1% + 9.2%). The 37.6% value for $\text{ProtConn}_{\text{Bound}}$ is as given by $\text{ProtConn}_{\text{Bound}}$ = Prot - ProtUnconn[Design] = 46.8% - 9.2% or by $\text{ProtConn}_{\text{Bound}}$ = ProtConn + ProtUnconn[Sea] + ProtUnconn[Outland] = 34.5% + 0% + 3.1%.

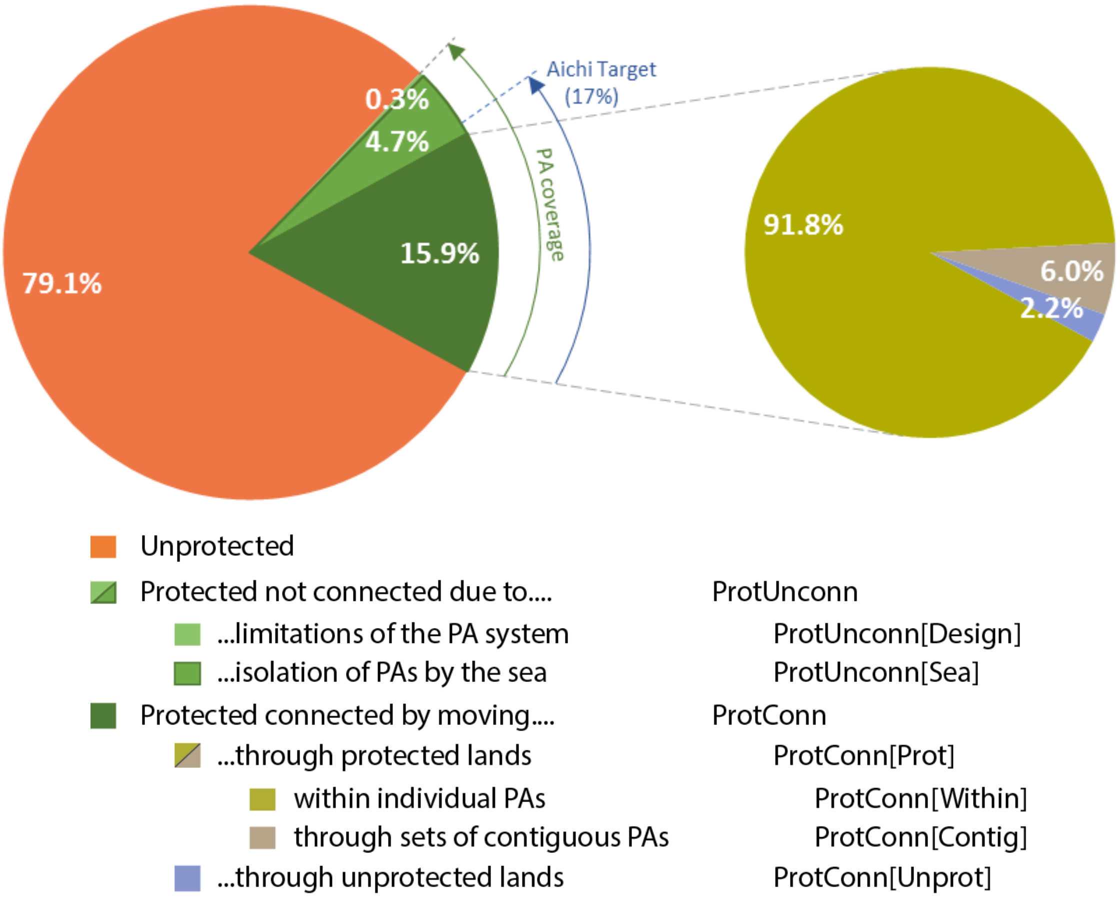

Fig. D3. Example for Antigua and Barbuda with the ProtConn-related indicators calculated for d_{med} = 10 km. While the non-adjusted ProtConn for Antigua and Barbuda is 15.9%, i.e. below the 17% target, $\text{ProtConn}_{\text{Bound}}$ equals 20.6%, i.e. clearly above the 17% target. In the island state of Antigua and Barbuda, ProtUnconn[Design] = 0.3%, ProtUnconn[Sea] = 4.7%, ProtUnconn[Outland] = 0% and ProtConn[Trans] = 0% (the latter two are hence not shown in the figure). PA coverage is 20.9% (Prot = 100% - 79.1% = 15.9% + 4.7% + 0.3%). The 20.6% value for $\text{ProtConn}_{\text{Bound}}$ is given by $\text{ProtConn}_{\text{Bound}}$ = Prot - ProtUnconn[Design] = 20.9% - 0.3% or by $\text{ProtConn}_{\text{Bound}}$ = ProtConn + ProtUnconn[Sea] = 15.9% + 4.7%.

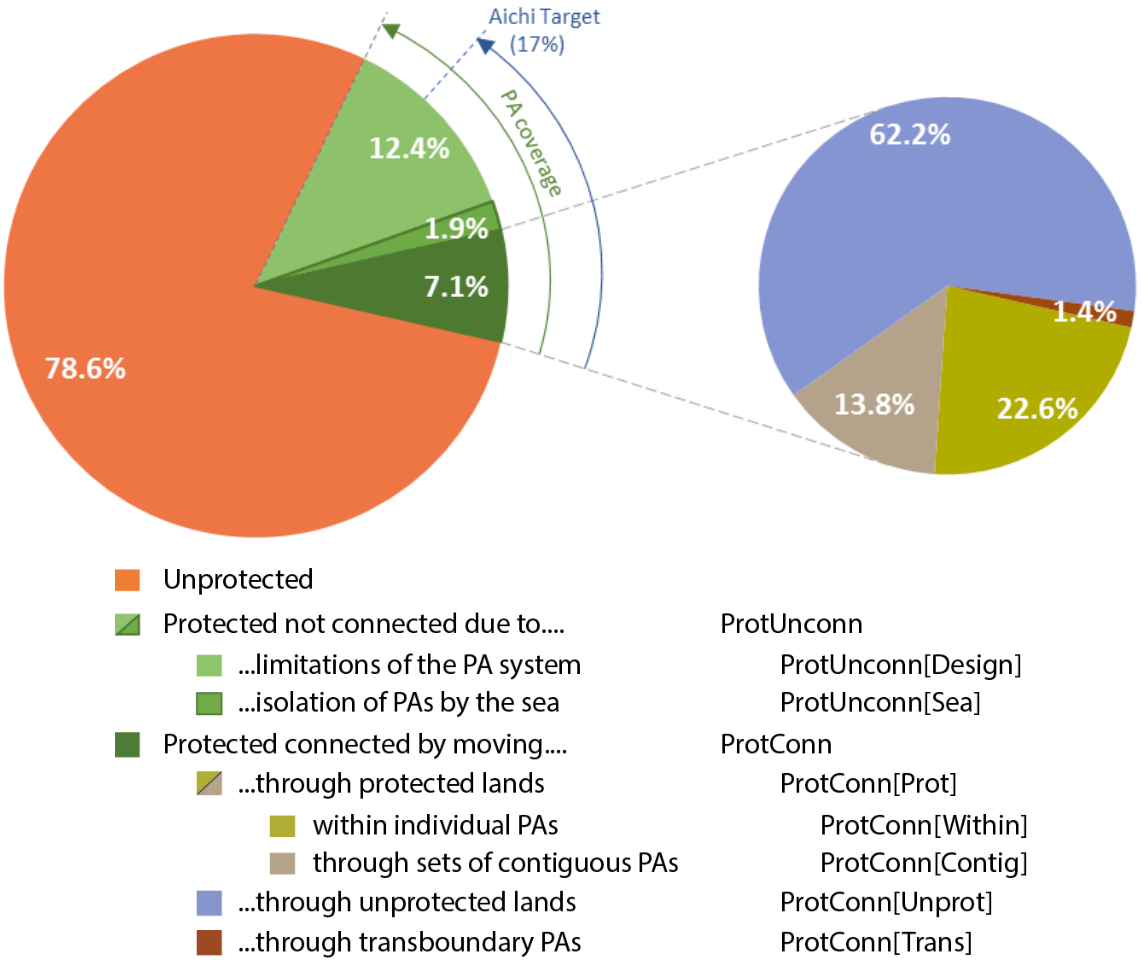

Fig. D4. Example for Italy with the ProtConn-related indicators calculated for d_{med} = 10 km. The non-adjusted ProtConn for Italy is 7.1%, while $\text{ProtConn}_{\text{Bound}}$ equals 9.0% (below the 17% target in both cases). In Italy, ProtUnconn[Outland] is very small (0.009%) and hence it is not shown in this figure and does not make a noticeable difference for the final $\text{ProtConn}_{\text{Bound}}$ value. PA coverage is 21.4% (Prot = 100% - 78.6% = 7.1% + 1.9% + 12.4%). The 9.0% value for $\text{ProtConn}_{\text{Bound}}$ is given by $\text{ProtConn}_{\text{Bound}}$ = Prot - ProtUnconn[Design] = 21.4% - 12.4% or by $\text{ProtConn}_{\text{Bound}}$ = ProtConn + ProtUnconn[Sea] = 7.1% + 1.9%.

Appendix E. Global and regional averages of the ProtConn indicators for other dispersal distances

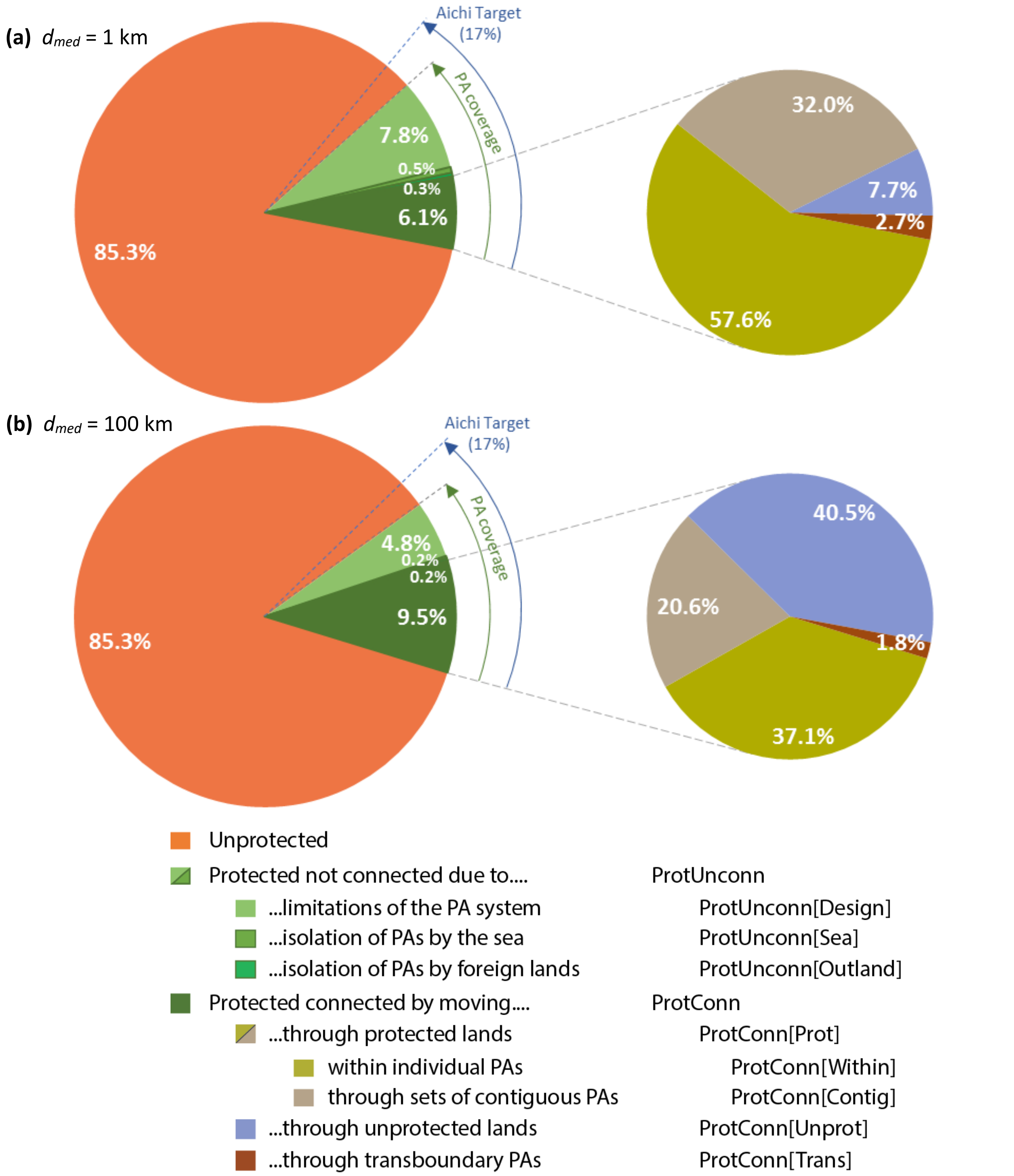

Fig. E1. Global values of the ProtConn-related indicators for a median dispersal distance of d_{med} = 1 km (a) and of d_{med} = 100 km (b), obtained as a weighted average of the calculated country-level values (see Methods). The global PA coverage is 14.7% (100 - 85.3%), while ProtConn = 6.1% for d_{med} = 1 km and ProtConn = 9.5% for d_{med} = 100 km. ProtUnconn[Design] = 7.8% for d_{med} = 1 km and ProtUnconn[Design] = 4.8% for d_{med} = 100 km; therefore, the level of connectivity bounded to the efforts that can be really made by the countries is $ProtConn_{Bound}$ = 6.9% (14.7 - 7.8%) for d_{med} = 1 km and 9.9% (14.7 - 4.8%) for d_{med} = 100 km. The values for ProtUnconn[Sea] (0.5% for d_{med} = 1 km, 0.2% for d_{med} = 100 km) and for ProtUnconn[Outland] (0.3% for d_{med} = 1 km, 0.2% for d_{med} = 100 km) are shown in a smaller font size due to their lower magnitude compared to the other ProtConn-related values in the left-hand pie charts. A pie chart with the global values for d_{med} = 10 km is provided in Figure 1 in the main text.

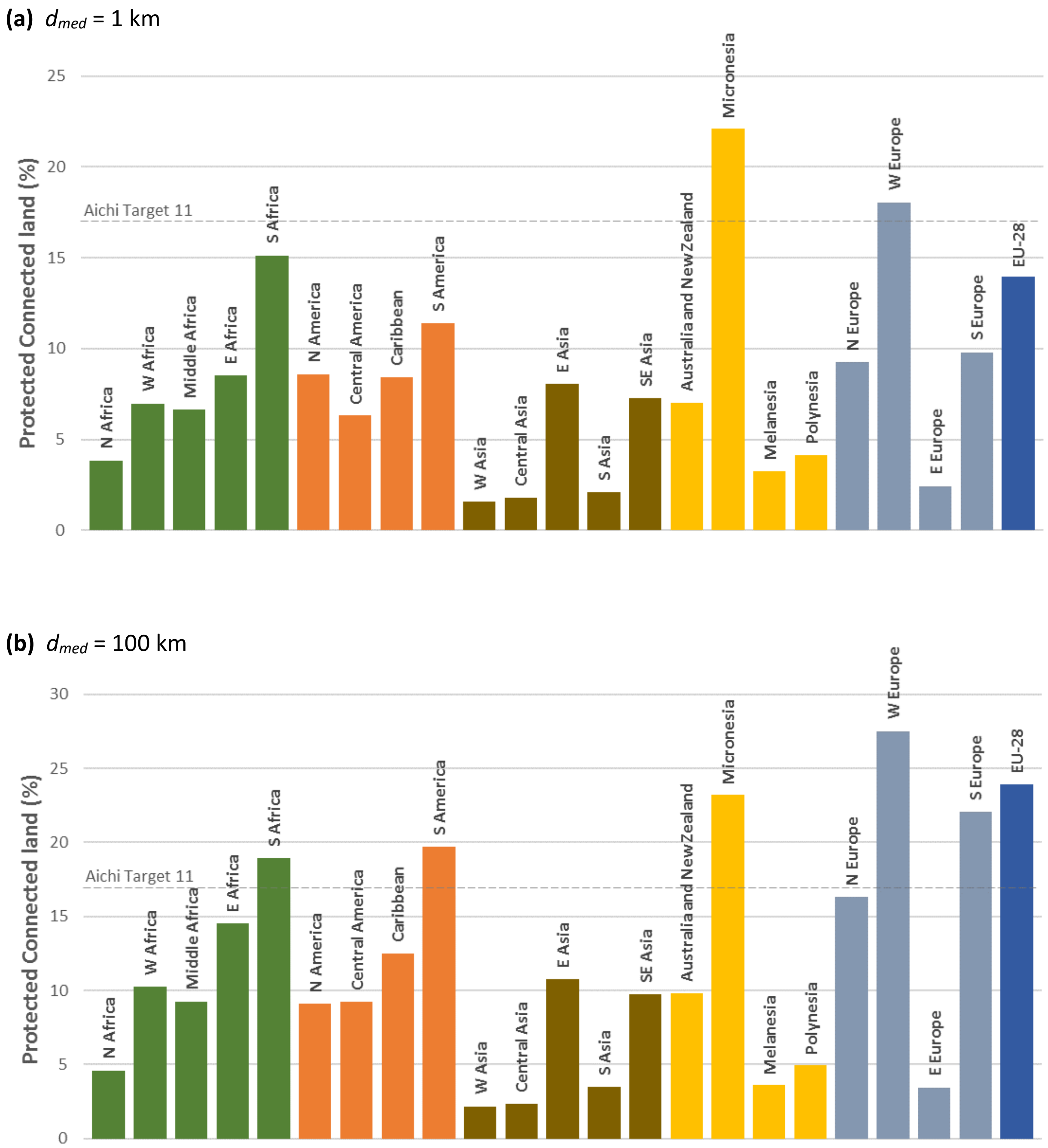

Fig. E2. Protected Connected indicator considering the part of the PA connectivity that is in the power of a country to influence ($ProtConn_{Bound}$) for all regions of the world and for the Europan Union (EU-28) for a median dispersal distance d_{med} = 1 km (a) and d_{med} = 100 km (b). Note that the Russian Federation is included within Eastern Europe, which has a large influence on the values for this region (see Methods). These $ProtConn_{Bound}$ values were aggregated at the regional level using a weighted average of the calculated country values (see Methods). The regional-level $ProtConn_{Bound}$ values for d_{med} = 10 km are shown in Figure 3 in the main text.

Appendix F. Continental averages of the ProtConn indicators

The country-level values of the ProtConn-related indicators were aggregated at the continental level, using a weighted average of country values (see Methods). This appendix shows the resultant continental-level values for $ProtConn_{Bound}$ (Fig. F1), ProtConn[Within] (Fig. F2), ProtConn[Contig] (Fig. F3), ProtConn[Unprot] (Fig. F4) and ProtConn[Trans] (Fig. F5) for a reference median dispersal distance of 10 km.

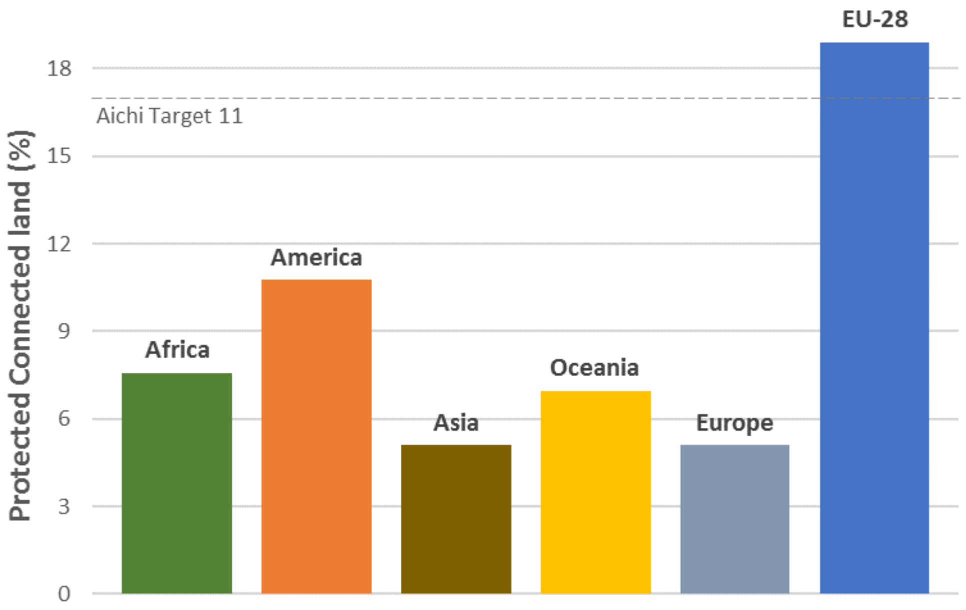

Fig. F1. Protected Connected indicator considering the part of the PA connectivity that is in the power of a country to influence ($ProtConn_{Bound}$) for all continents and for the European Union (EU-28) for a reference median dispersal distance of 10 km. Note that the Russian Federation is included within Europe, which has a large influence on the values for this continent (see Methods).

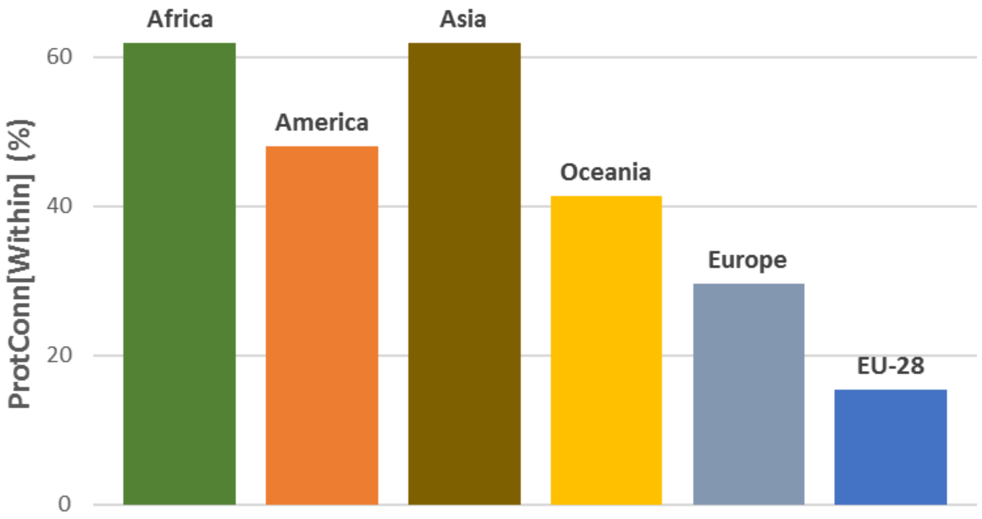

Fig. F2. Percentage of the protected connected land that can be reached within individual PAs, as assessed for all continents and for the EU-28 by aggregating the country-level values of ProtConn[Within] for a reference median dispersal distance of 10 km.

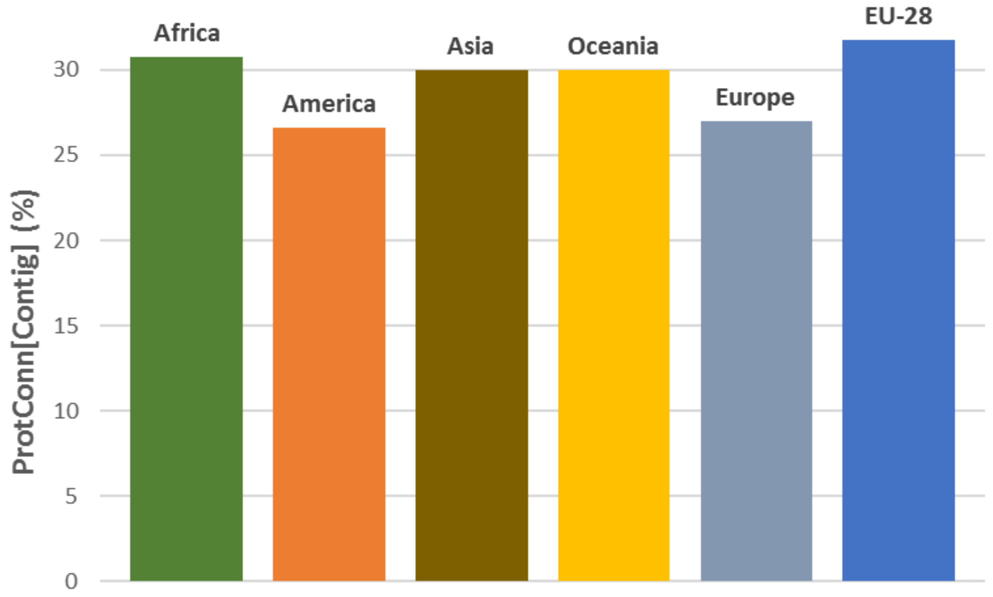

Fig. F3. Percentage of the protected connected land that can be reached by moving through adjacent PAs with different designations, as assessed for all continents and for the EU-28 by aggregating the country-level values of ProtConn[Contig] for a reference median dispersal distance of 10 km.

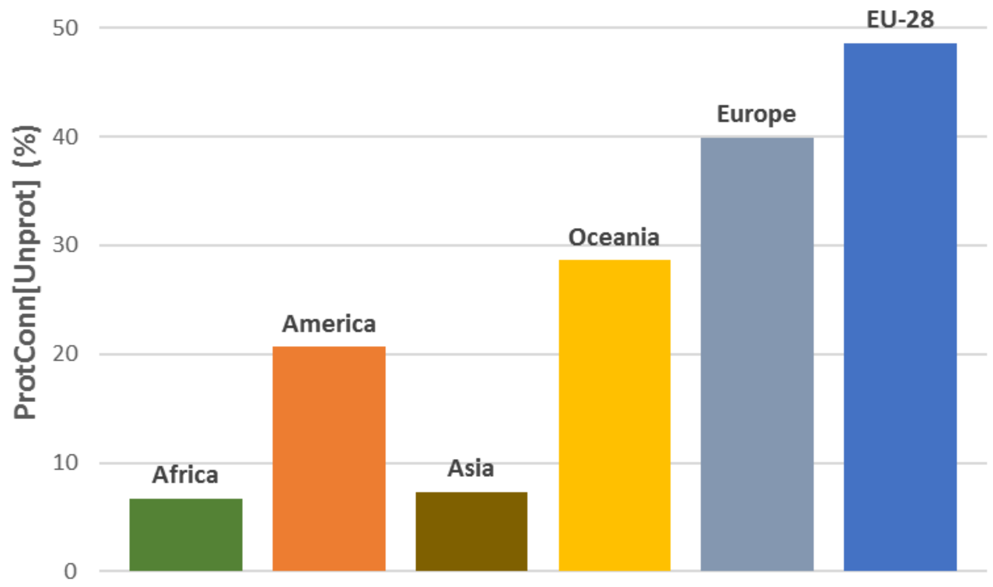

Fig. F4. Percentage of PA connectivity that depends on movement through unprotected lands, as assessed for all continents and for the EU-28 by aggregating the country-level values of ProtConn[Unprot] for a reference median dispersal distance of 10 km.

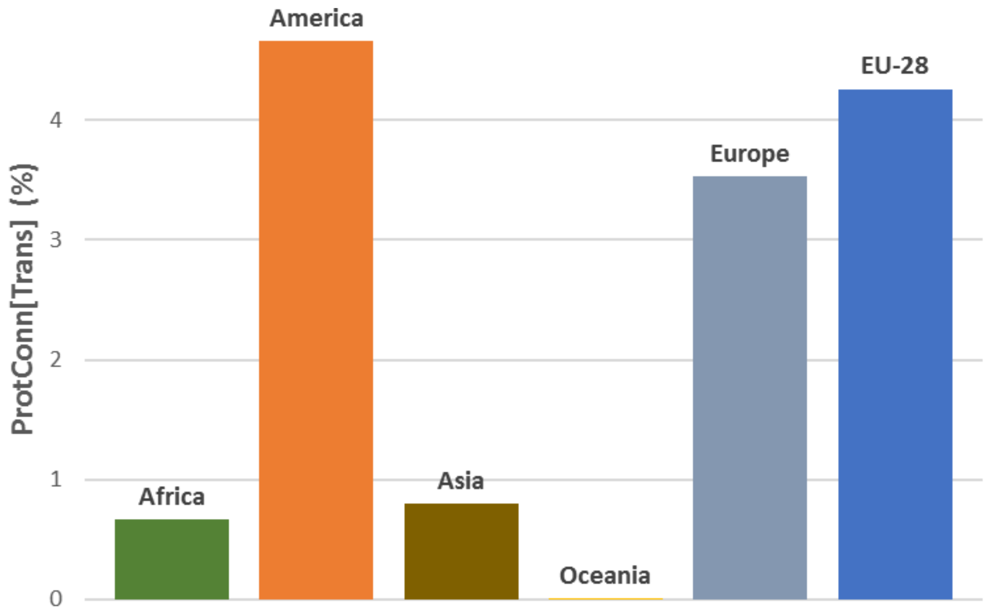

Fig. F5. Percentage of PA connectivity that depends on transnational linkages, i.e. on using PAs outside a country when moving between two PAs of the country, as assessed for all continents and for the EU-28 by aggregating the country-level values of the transboundary fraction of ProtConn (ProtConn[Trans]) for a reference median dispersal distance of 10 km.

Appendix G. Examples of countries with different priorities for PA connectivity

This appendix shows several detailed and illustrative examples of countries falling within each of the priority categories for PA connectivity (Table 2 and Figure 6 in the main text). One example country is provided for each of these priorities, including a map of the PAs in the country and pie charts summarizing the values of the ProtConn-related indicators for the country.

Note that, as for the indicator values calculated in this study, all the examples below are based on the public version of the World Database on Protected Areas (WDPA) of June 2016, and only consider the terrestrial PAs in each country (see Methods for further details). The reported indicator values in the pie charts below are all based on a reference median dispersal distance of d_{med} = 10 km, as in the classification in Table 2 and Figure 6. The country boundaries, and the delineation of disputed territories (those with unsettled sovereignty) shown in the figures below are all based on GAUL 2015 (see Methods), but this does not imply any endorsement by the authors, nor any official position by the European Commission, on the sovereignty of any of these lands. Finally, we here use for simplicity, as in the rest of the study, the term 'countries' to refer to those territories for which PA systems have been individually considered in the analysis, as given by their ISO3 codes reported in the WDPA (see Methods). However, in several cases these ISO3 codes correspond to territories under the sovereignty of other nations; for example in the case of Greenland, a self-governing territory that is part of the Kingdom of Denmark.

The following seven countries have been selected as informative examples, here sequentially numbered as G1-G7, and corresponding to the coding of priorities in Table 2 and Figure 6 in the main text (A1, A2, B1+B2, B1, B2, B3, C):

- G1. A country in which a general increase of PA coverage is a priority (A1): Turkey.
- G2. A country in which the targeted designation of connecting PAs is a priority (A2): Cameroon.
- G3. A country in which the permeability of unprotected lands and the coordinated management of adjacent PAs are priorities (B1+B2): Spain.
- G4. A country in which the permeability of unprotected lands is a priority (B1): New Zealand.
- G5. A country in which the coordinated management of adjacent PAs is a priority (B2): Bhutan.
- G6. A country with no specific priority other than PA management effectiveness for connectivity (B3): Greenland.
- G7. A country in which the coordinated management of transboundary PA linkages is a priority (C): Nepal.

These examples are presented below.

G1. An example of a country in which a general increase of PA coverage is a priority (A1): Turkey

As shown in Figure G1, based on the PAs reported by Turkey to the World Database on Protected Areas (WDPA), the country has a very low PA coverage (Prot = 0.21%). Although some sources report a higher PA coverage (Küçük and Ertürk, 2013; Ministry of Forestry And Water Affairs, 2014), other recent global assessments using the WDPA have also reported this low PA coverage of 0.2% (UNEP-WCMC and IUCN, 2016). In any case, the PA system as reported in the WDPA is a configuration which is useful to illustrate the A1 priority. This PA system consists of very few, small, and distant PAs, which results in very low connectivity, with $ProtConn_{Bound}$ = 0.07%. This connectivity is very low because the PAs are individually small, so that little protected land can be reached by moving within the limits of these individual PAs, and because they are separated from each other by very large distances (compared to the species dispersal distances), so that successful movements between the different PAs are rather unlikely (Fig. G1a). Even for the largest dispersal distance considered, which is d_{med} = 100 km, $ProtConn_{Bound}$ only reaches 0.1%. Even if all the existing PAs were located together in a single PA or in a local cluster of PAs with good inter-PA connectivity, they would jointly provide only a small amount of reachable protected land, never larger than the 0.21% of PA coverage. Therefore, a well-connected PA system in which it is possible to use and reach a sufficiently large amount of protected land can never be achieved unless a large-scale increase in the coverage of PAs (as currently reported in the WDPA) is accomplished in Turkey.

Given the very large tracts of unprotected lands separating most of the PAs compared to the considered species dispersal abilities, there is a low likelihood of successful dispersal between these PAs, which translates into a low value of the ProtConn[Unprot] fraction (4.8%, see Fig. G1b). In fact, nearly all of the protected land that can be reached from each protected location is confined to the limits of an individual PA, as given by ProtConn[Within] = 95.2% (Fig. G1b).

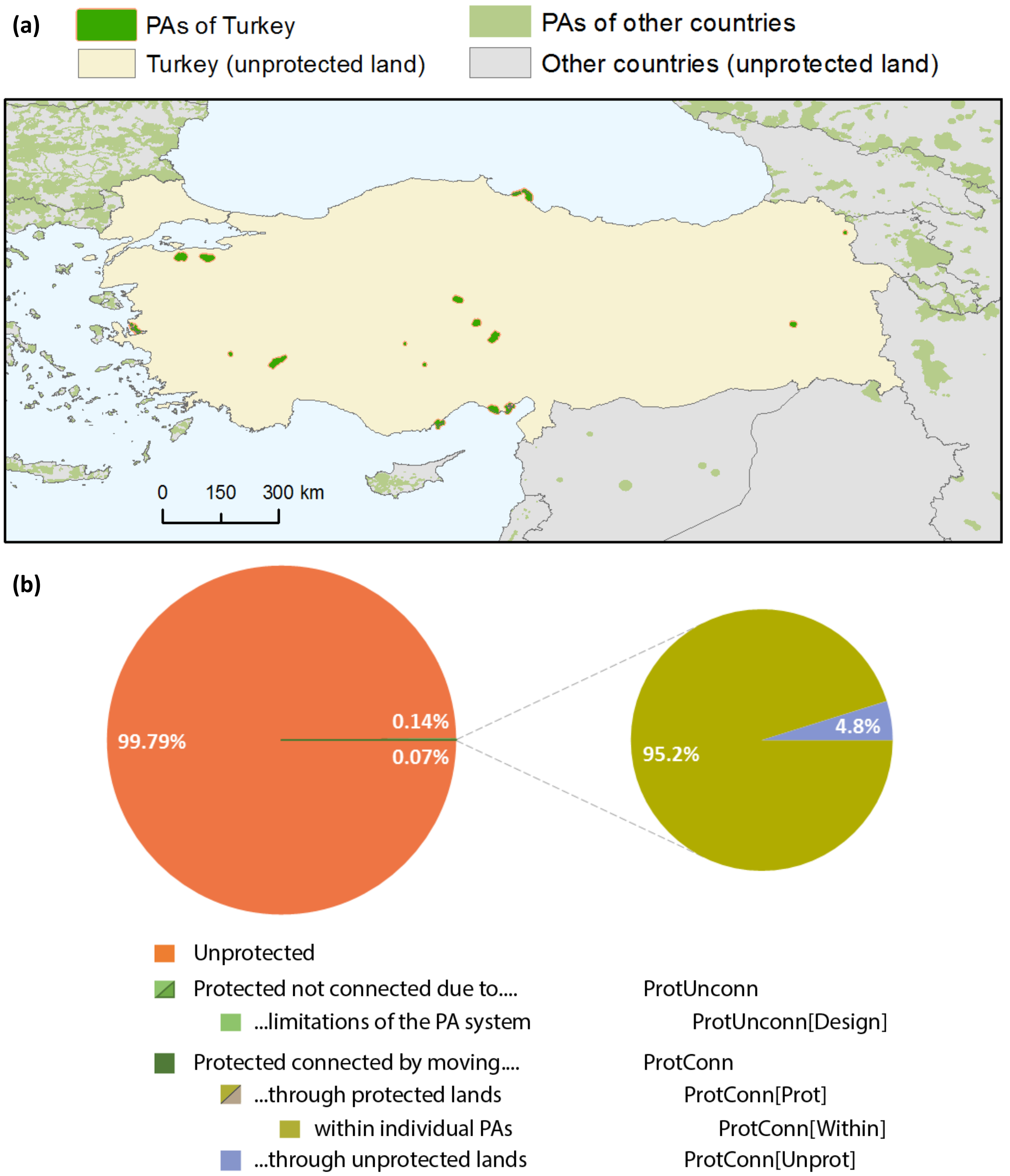

Fig. G1. Protected areas in Turkey and neighbouring countries, as reported in the WDPA (a) and ProtConn pie charts for Turkey and d_{med} = 10 km (b). PAs shown in (a), and the indicator values in (b), are as given by the WDPA of June 2016. Using the information in the WDPA of June 2016, in Turkey the PA coverage is very low (0.21% = 100% - 99.79%) and most of it is unconnected (ProtUnconn[Design] = 0.14%), with ProtConn = 0.07%. ProtUnconn[Sea] = 0 and ProtUnconn[Outland] = 0. Therefore, $ProtConn_{Bound}$ = ProtConn = 0.07%. On the other hand, ProtConn[Trans] = 0 and ProtConn[Contig] = 0.02%; because of their very low values, these two fractions are not shown in the right pie chart for Turkey in (b).

G2. An example of a country in which the targeted designation of connecting PAs is a priority (A2): Cameroon

Cameroon currently has a PA coverage of 10.9%, below the 17% level of Aichi Target 11 for year 2020 (Fig. G2b). However, the performance of the PA system is much weaker regarding connectivity: more than two thirds of the protected lands are not connected, giving a $ProtConn_{Bound}$ of only 3.0% for d_{med} = 10 km (Fig. G2b). This low $ProtConn_{Bound}$ is the result of PAs being scattered throughout the country, generally quite distant from each other, and separated by large tracts of unprotected land (Fig. G2a). The unprotected distances that need to be traversed between PAs are very large compared to the species dispersal distance considered, which results in a low ProtConn[Unprot] of 4.5% and in the reported low $ProtConn_{Bound}$ value (Fig. G2b). If we consider the two largest median dispersal distances for which the connectivity indicators have been calculated in this study (d_{med} = 30 km and d_{med} = 100 km), $ProtConn_{Bound}$ increases but still remains low compared to PA coverage in the country ($ProtConn_{Bound}$ is 3.6% and 5.6% for d_{med} = 30 km and d_{med} = 100 km, respectively).

Given these numbers, the lack of PAs as compared to the 17% target is significant in Cameroon: there is a shortfall in PA coverage of 6.1% (17 - 10.9%). This shortfall in PA coverage is, however, smaller than the percentage of the currently protected land that is not connected because of limitations in the design of the PA system of the country, which equals ProtUnconn[Design] = 7.9% for the reference dispersal distance of d_{med} = 10 km (Fig. G2b). Therefore, further development of the PA system in Cameroon should put an important focus on the targeted designation of PAs in strategic locations for connectivity, so that they can act as corridors or stepping stones between those PAs that are already designated. Such spatially-targeted designation should aim to fill the large unprotected gaps existing between several of the important PAs of Cameroon (Fig. G2a), following for instance the example of Bhutan (see section G5 below), and thereby contributing to a more functional and effective network of PAs in the country.

Regarding the ProtConn fractions in Cameroon (Fig. G2b), the ProtConn[Within] fraction is the dominant one (88.3% of ProtConn); given the difficulty of inter-PA movements due to the large distances separating many of the PAs (ProtConn[Unprot] is only 4.5%), most of the protected land that can be reached by a species is confined to that available within the source PA. The ProtConn[Contig] fraction is low but still noteworthy, with a value of 7.0% (Fig. G2b), which is mainly due to a few adjacent and relatively large PAs in the south of the country; almost all other PAs in the centre and north of Cameroon are not directly adjacent (Fig. G2b). Finally, the ProtConn[Trans] fraction is low because, although there are several PAs in neighbouring countries that are close or adjacent to other PAs in Cameroon, the movement between the PAs of Cameroon (i.e. when the source and destination PAs are both in Cameroon) does not depend to any large extent on using PAs in other countries along the way.

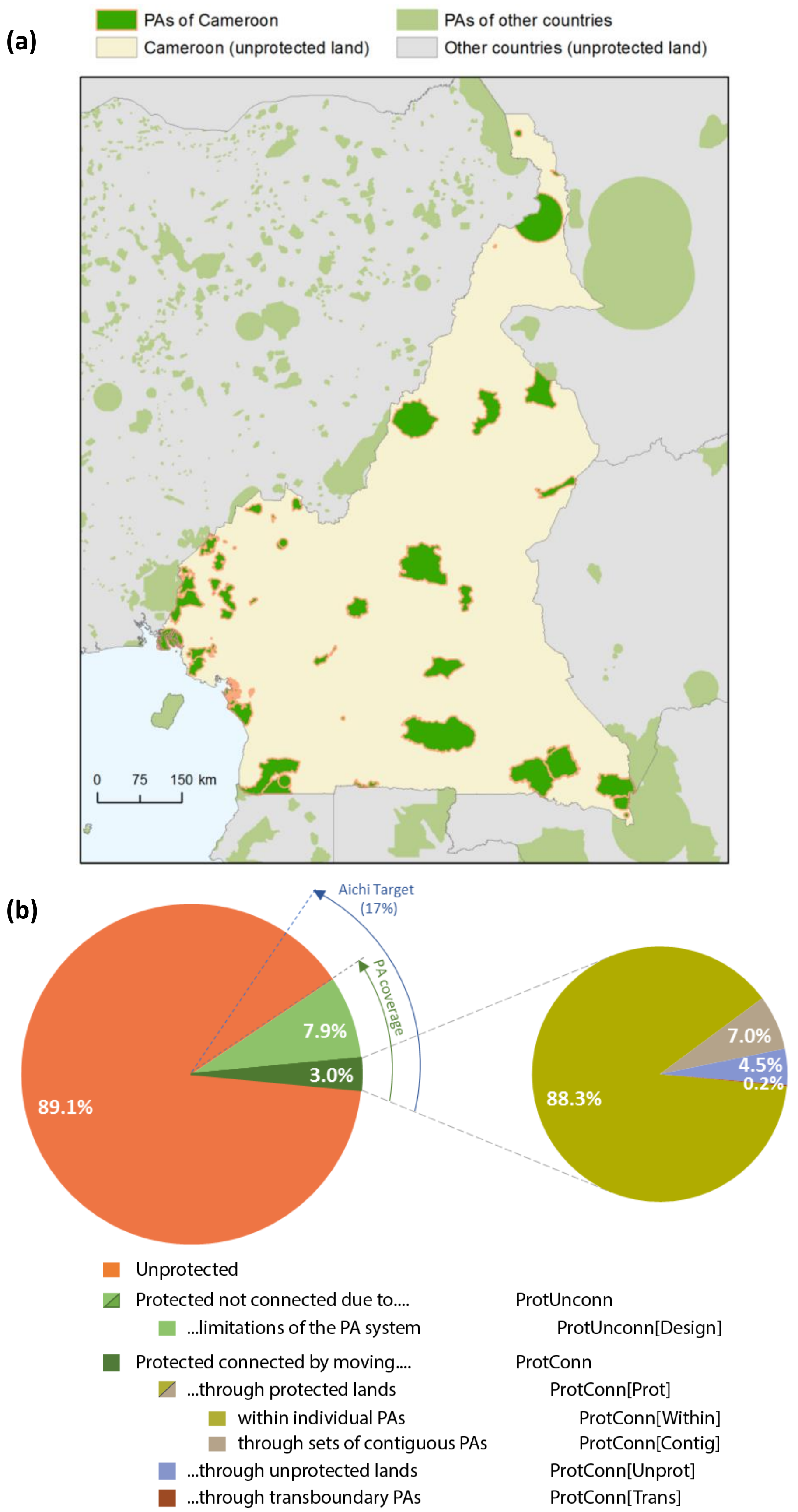

Fig. G2. Protected areas in Cameroon and neighbouring countries (a) and ProtConn pie charts for Cameroon and d_{med} = 10 km (b). PAs shown in (a), and the indicator values in (b), are as given by the WDPA of June 2016. Cameroon has a PA coverage (Prot) of 10.9% (100% - 89.1%), but a $\text{ProtConn}_{\text{Bound}}$ value of only 3.0%, due to a comparatively large ProtUnconn[Design] = 7.9%, as shown in (b). ProtUnconn[Sea] = 0 and ProtUnconn[Outland] = 0 in Cameroon (hence not shown in the left pie chart), which makes $\text{ProtConn}_{\text{Bound}}$ = ProtConn. For small PA polygons in (a) the pink edge colour dominates, making these PAs appear as fully in that colour at this scale of visualization.

G3. An example of a country in which the permeability of unprotected lands and the coordinated management of adjacent PAs are priorities (B1+B2): Spain

Spain has a well-developed PA system regarding PA coverage (Prot = 27.9%), and also has a $ProtConn_{Bound}$ value that is already clearly above the 17% target for year 2020 ($ProtConn_{Bound}$ = 19.9%), as shown in Figure G3b. The PA system in Spain has however a strong dependency on the permeability of unprotected landscapes, given that ProtConn[Unprot] makes up 58.1% of the total ProtConn value (Fig. G3b) and also, though to a comparatively lower extent, on the coordinated management of adjacent PAs, with ProtConn[Contig] = 24.2% (Fig. G3b). This is because, in Spain, individual PAs are usually embedded in unprotected landscapes and are generally small; ProtConn[Within] is only 10.0% (Fig. G3b), which is considerably below the values of this fraction in other countries (Fig. 4a) or regions (Fig. 5a).

Because many of the PAs are small, it is unlikely that they are sufficient to individually ensure some of the conservation goals for which they were declared. Meeting these goals will only be possible if PAs function as an effective network of linked sites, which necessarily involves the conservation or restoration of green infrastructure elements in the unprotected landscapes. Promoting the permeability of the lands located in between PAs is, therefore, a priority for Spain, as well as for other countries in a similar situation and classified as B1 priority (or B1+B2) in Figure 6. Coordinated management of different adjacent PAs for connectivity (priority B2) would also help to compensate for the relatively small size of PAs in Spain, concatenating them into coherent movement pathways supporting larger-scale ecological flows and processes throughout the country.

Spain is also within the set of countries that need to pay significant attention to the functionality and coordinated management of transboundary linkages with PAs in other countries (priority C). This is because the connectivity between Spain´s PAs depends significantly on movement through PAs in Portugal and France (Fig. G3a), as indicated by the ProtConn[Trans] = 7.7%, which is much higher than in other countries (Fig. 4d) or regions (Fig. 5d).

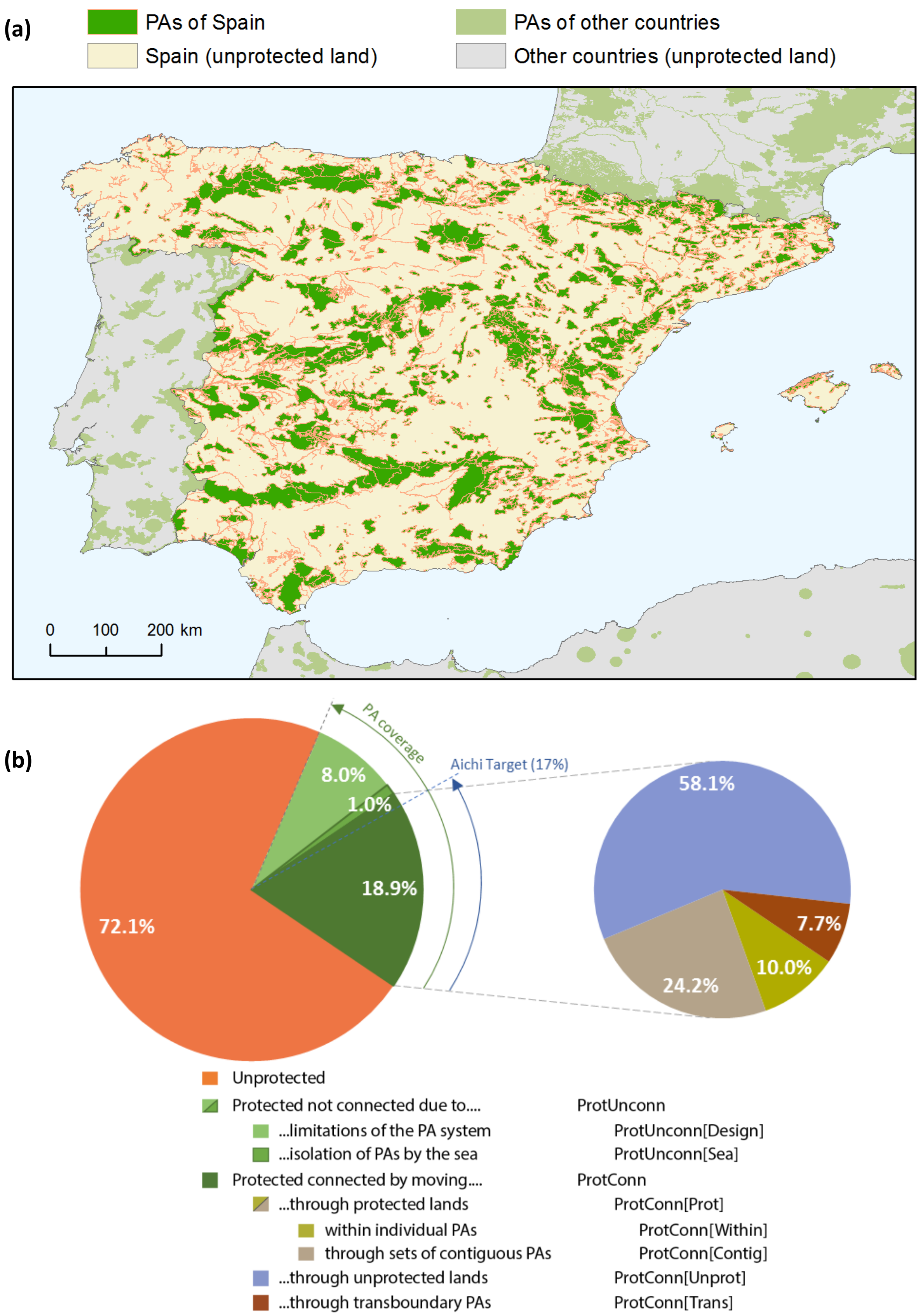

Fig. G3. Protected areas in Spain and neighbouring countries (a) and ProtConn pie charts for Spain and d_{med} = 10 km (b). PAs shown in (a) are as given by the WDPA of June 2016. In Spain, ProtConn = 18.9%, ProtUnconn[Sea] = 1.0%, and ProtUnconn[Outland] is almost negligible (0.0002%), which gives $\text{ProtConn}_{\text{Bound}}$ = 19.9%. Both the PA coverage in Spain (27.9% = 18.9% + 1.0% + 8.0% = 100% - 72.1%) and $\text{ProtConn}_{\text{Bound}}$ are above the 17% level of Aichi Target 11. For small PA polygons in (a) the pink edge colour dominates, making these PAs appear as fully in that colour at this scale of visualization. Note that the Canary Islands are not shown in Figure G3a but that they contribute to the values for Spain reported in Figure G3b and elsewhere in this study.

G4. An example of a country in which the permeability of unprotected lands is a priority (B1): New Zealand

The PA system of New Zealand has already achieved almost two-fold the 17% level of Aichi Target 11 in terms of PA coverage (Prot = 32.1%), and it also scores high in PA connectivity ($ProtConn_{Bound}$ = 28.2%) (Fig. G4b). However, the connectivity of the PA system in New Zealand strongly depends on ensuring the permeability of the unprotected landscapes, with ProtConn[Unprot] = 46.3% (Fig. G4b), much higher than in most other countries (Fig. 4c) and regions (Fig. 5c), but below the levels for Spain (Fig. G3b). Note that ProtConn[Unprot] = 46.3% is an overall value at the level of the entire country, but that the dependency on unprotected landscapes is significantly higher in the North Island than in the South Island. The North Island is more highly populated and has smaller PAs than the South Island (Fig. G4a). In the South Island, some large or very large PAs are found along the Southern Alps of New Zealand, providing comparatively larger tracts of land that can be reached without traversing any unprotected land (Fig. G4a). There are, however, some PAs towards the southeast of the South Island that are separated by relatively large portions of unprotected lands from the rest of the larger PAs in the Southern Alps (Fig. G4a). The most prominent example of these large PAs along the Southern Alps is Te Wahipounamu – South West New Zealand, a World Heritage Site in the southwest of the South Island that covers ≈26,000 km^2, and encompasses other large PAs such as the National Parks of Fiordland (≈12,800 km^2) and Mount Aspiring (≈3,600 km^2). Large PAs in this part of New Zealand are mainly responsible for the overall ProtConn[Within] = 38.8% in New Zealand (Fig. G4b), which is higher than in some other countries (Fig. 4a) and particularly than in those countries that have B1 as a priority; for example, Spain, whose ProtConn[Within] = 10.0% (Fig. G3b).

The ProtConn[Contig] fraction for New Zealand has a significant value of 14.9%, which suggests some importance of coordinated management for connectivity of sets of adjacent PAs in the country. This value, however, is not above the global average, and hence B2 is not highlighted as a top priority in New Zealand, by contrast with Spain (Fig. G3) and some other countries (Fig. 6).

Note that in New Zealand there is a remarkable natural isolation of PAs by the sea, which mainly weakens the connections between PAs located in the North and South Islands, and to a lesser extent (given the smaller relative size of the PAs affected) the connections of Rakiura National Park and other PAs in Stewart Island with the rest of the PAs in New Zealand. This isolation of PAs by the sea results in a ProtUnconn[Sea] = 5.6% (Fig. G4b), which is amongst the highest in the world (within the 10% of countries with highest ProtUnconn[Sea]).

(a)

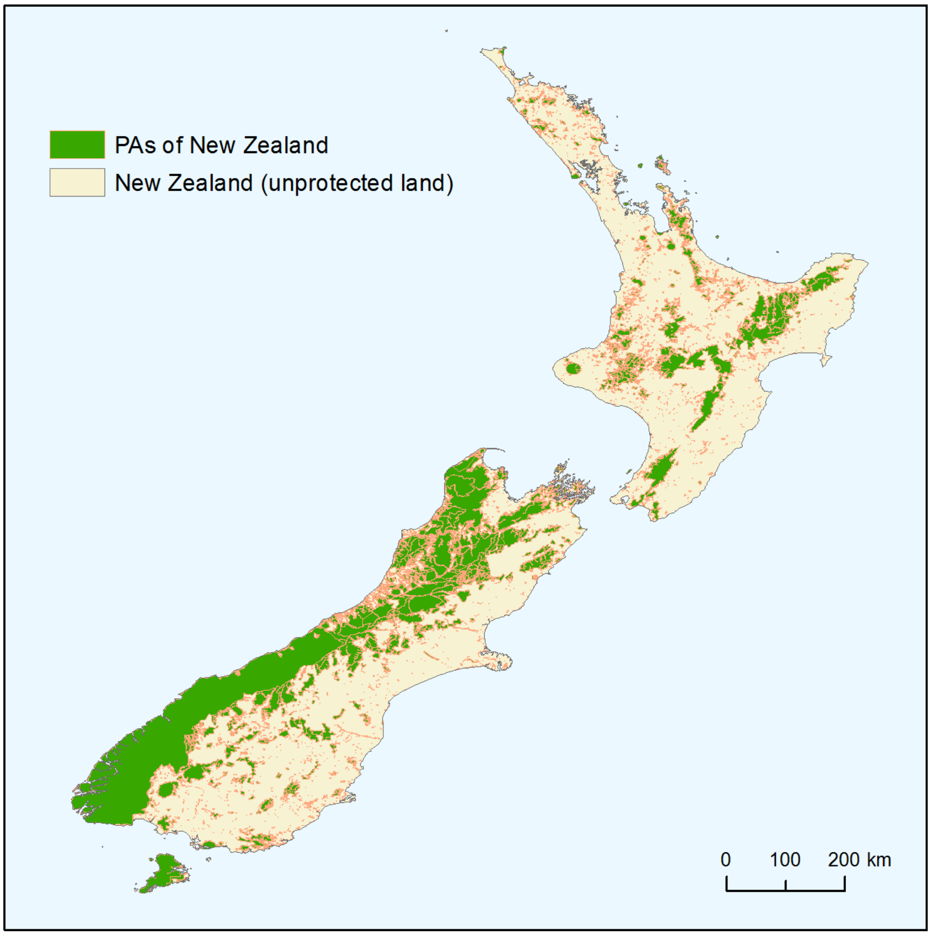

(b)

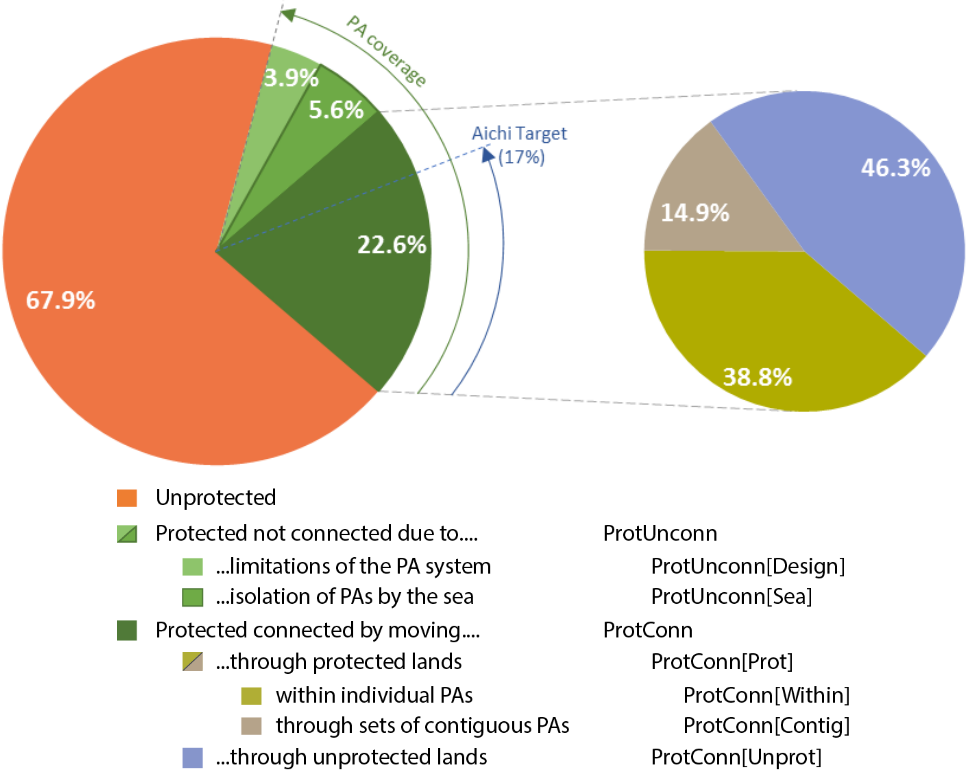

Fig. G4. Protected areas in New Zealand (a) and ProtConn pie charts for New Zealand and d_{med} = 10 km (b). PAs shown in (a) are as given by the WDPA of June 2016. In New Zealand, ProtConn = 22.6%, ProtUnconn[Sea] = 5.6%, and ProtUnconn[Outland] = 0, which gives $\text{ProtConn}_{\text{Bound}}$ = 28.2%. Both the PA coverage in New Zealand (32.1% = 22.6 + 5.6% + 3.9% = 100% - 67.9%) and $\text{ProtConn}_{\text{Bound}}$ = 28.2% are well above the 17% level of Aichi Target 11. For small PA polygons in (a) the pink edge colour dominates, making these PAs appear as fully in that colour at this scale of visualization.

G5. An example of a country in which the coordinated management of adjacent PAs is a priority (B2): Bhutan

PAs in Bhutan are all connected to each other, either directly or through other intermediate PAs, so that movement between all PAs is possible without traversing any unprotected lands (Fig. G5a). There is no PA isolation due to limitations in the design of the PA system in the country. In addition, ProtUnconn[Sea] = 0 and ProtUnconn[Outland] = 0. Therefore, the Protected Connected indicator has the same value as Prot (PA coverage), i.e. $ProtConn_{Bound}$ = ProtConn = Prot = 50.7% (Fig. G5b). In this sense, Bhutan has not only a high PA coverage but also an optimum design of the PA system regarding connectivity, almost tripling the 17% level of Aichi Target 11 for 2020 both for PA coverage and connectivity. In fact, of the 20 PAs in Bhutan, eight are deliberately designated and named as Biological Corridors, which gives one of the most remarkable country examples of how PAs can be established to link the entire set of PAs as a connected system. The Biological Corridors were designated in 1999, recognizing that even though the individual PAs were important there was a need to consider them as “building blocks” of the overall conservation landscape rather than as independent conservation units (Wangchuck, 2007). These Biological Corridors have been reported to be used by different animal species, particularly by large mammals such as tigers, leopards and takins (Wangchuck, 2007). The combination of these Biological Corridors with other PAs in the country (National Parks, Wildlife Reserves, etc.) forms the Bhutan Biological Conservation Complex (B2C2) as described by Wangchuck (2007). As in all cases and countries, the ProtConn levels reported here, and the actual functionality of these Biological Corridors, will depend on the effective conservation and management of these PAs, which is assumed by the ProtConn indicator and is hence a priority to be ensured by Bhutan and by all other countries.

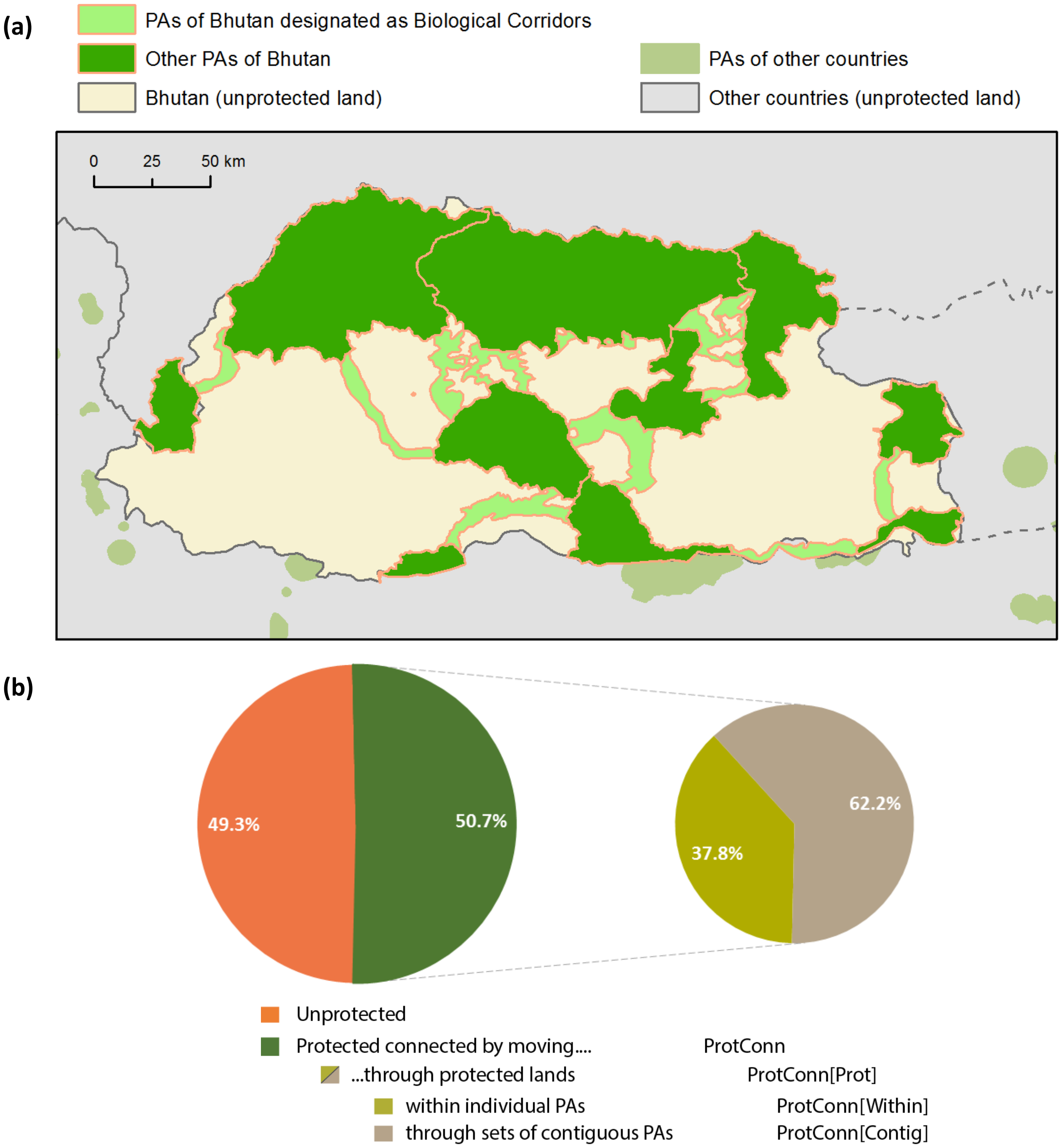

Fig. G5. Protected areas in Bhutan and neighbouring countries[i] (a) and ProtConn pie charts for Buthan and d_{med} = 10 km (b). PAs shown in (a) are as given by the WDPA of June 2016. In Bhutan, ProtUnconn[Sea] = 0, ProtUnconn[Outland] = 0 and ProtUnconn[Design] = 0[ii], which gives $ProtConn_{Bound}$ = ProtConn = Prot = 50.7%. Two of the fractions of ProtConn, ProtConn[Unprot] and ProtConn[Trans], are also 0[ii]. Altogether, this considerably reduces the number of ProtConn-related indicators to be shown in the pie charts for Bhutan, as given by the simplified legend in (b).

---

[i] The country boundaries are as given by the GAUL 2015 layer (see Methods). The dashed lines to the east of Bhutan correspond to Arunachal Pradesh, a territory with unsettled sovereignty according to (and as mapped by) GAUL 2015 which is disputed by India and China. The delineation of the boundaries of the country and of the disputed territories here shown is therefore taken from GAUL 2015 and does not imply any endorsement by the authors, nor any official position by the European Commission, on the sovereignty of any of these lands (either if reported as disputed or as non-disputed by GAUL 2015).

[ii] To be fully precise, there is a small exception of a very small PA (with an area of 1.14 km^2) that is to some degree isolated (not connected through protected lands to other PAs and relatively distant from them), which in fact gives ProtConn[Unprot]=0.003% and ProtUnconn[Design] = 0.001% for Bhutan. These ProtConn[Unprot] and ProtUnconn[Design] values have been rounded to 0 in Figure G5b and in the related discussion in the text.

G6. An example of a country with no specific priority other than PA management effectiveness for connectivity (B3): Greenland

The PA coverage in Greenland is very high (Prot = 41.2%) and most of the protected land is connected and actually found within a single and very large PA (National Park) in the northeast of Greenland (Nationalparken i Nord- og Østgrønland), which has an area of ≈972,000 km² (Fig G6a). This translates to a $ProtConn_{Bound}$ value that is also very high and almost as large as Prot ($ProtConn_{Bound}$ = 40.5%), as shown in Fig. G6b. Both Prot and $ProtConn_{Bound}$ are well above twice the 17% level of Aichi Target 11 for year 2020.

Given that this large PA, alone, is responsible for the vast majority of the reachable protected land in Greenland, and of the reported $ProtConn_{Bound}$ value, the single priority for this territory is to ensure an effective conservation management for connectivity of this PA. Note that PA management effectiveness for connectivity is an assumption of the ProtConn indicator, and hence is a priority for all countries. The difference is that in the case of Greenland, the very dominant contribution of such a large (and assumed to be internally connected) PA gives a very high value to the ProtConn[Within] fraction, which is 98.1% (Fig. G6b). This very large ProtConn[Within] value overrides the other ProtConn fractions, which get very low values (Fig. G6b). Therefore, none of the other priorities that could hypothetically apply for countries above Aichi Target 11 (priorities B1, B2 or C) are specifically highlighted for Greenland, which remains with no other specific priority than PA management effectiveness for connectivity (B3).

The small value of ProtUnconn[Sea] = 0.2% is due to a few small PAs located in islands such as Disko Island (Qeqertarsuaq), which are separated by the sea from the rest of Greenland.

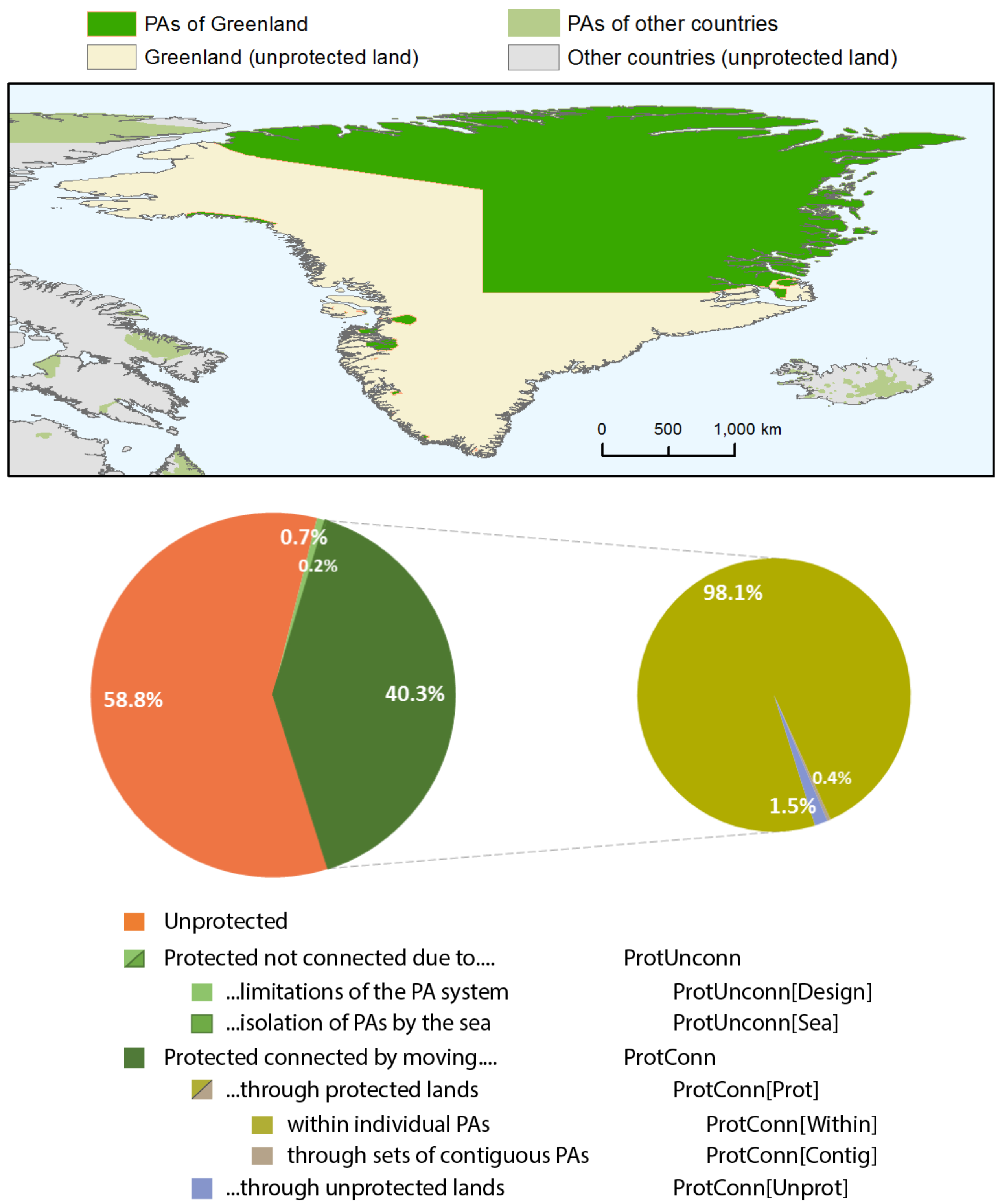

Fig. G6. Protected areas in Greenland and neighbouring countries (a) and ProtConn pie charts for Greenland and d_{med} = 10 km (b). PAs shown in (a) are as given by the WDPA of June 2016. In Greenland, ProtUnconn[Sea] = 0.2%, ProtUnconn[Outland] = 0, ProtUnconn[Design] = 0.7%, and ProtConn = 40.3%, which gives $\text{ProtConn}_{\text{Bound}}$ = 40.5%. PA coverage (Prot) is 41.2% (100% - 58.8% = 40.5% + 0.7%). ProtConn[Within]=98.1%, ProtConn[Unprot] = 1.5%, ProtConn[Contig] = 0.4%, and ProtConn[Trans] = 0 (this latter fraction is hence not shown in the right pie chart).

G7. An example of a country in which the coordinated management of transboundary PA linkages is a priority (C): Nepal

A considerable proportion of the connectivity between the PAs in Nepal is dependent on movement through PAs in other countries, as given by ProtConn[Trans] = 26.4% (Fig. G7b), the third largest ProtConn[Trans] value in the world (after the Czech Republic and Portugal). This is mainly because of the connecting role that the Quomolangma Nature Preserve in China (a large PA along the north-northeast border of Nepal, see Fig. G7a) has in linking several important PAs in Nepal. For example, it links the Annapurna and Manaslu Conservation Areas with Langtang National Park, and links the Sagarmatha and Makalu-Barun National Parks with the Kangchenjunga Conservation Area. This latter PA is in fact part of the Kangchenjunga Landscape, which is shared by Bhutan, India and Nepal, and is considered one of the most important transboundary landscapes in the eastern Himalayas (Chettri et al., 2007). PAs in the west-southwest of Nepal are also connected by PAs outside Nepal, though to a lower extent. In particular, the connectivity between the Bardia National Park and the Shuklaphanta Wildlife Reserve (both in Nepal) is enhanced by the Dudhwa National Park and the Katarniaghat Wildlife Sanctuary (both in India), which can be used as stepping stones or protected pathways in movements between the Nepalese PAs. These PAs are part of the Teraic Arc Landscape, a transnational region that has been highlighted as important for tiger conservation efforts and other connectivity-related processes (Harihar and Pandav, 2012; Chanchani et al., 2014). Therefore, the connectivity of these PAs in Nepal, and of a significant portion of the PA system in Nepal, will considerably benefit from a coordinated transboundary management with specific PAs in China and India that ensures the functionality and actual connectivity benefits of these transnational linkages.

While in Nepal the PA coverage (Prot) is already above the 17% level of Aichi Target 11 (Fig. G7b), there is still a shortfall on PA connectivity as measured by $ProtConn_{Bound}$ ($ProtConn_{Bound}$ = 14.6% < 17%), as shown in Figure G7b. This shortfall is mainly because, despite the good size and coverage of PAs in Nepal, several of them are separated by large tracts of unprotected landscapes; this is particularly true for the large distances to be traversed in movements from the PAs along the northern border of the country to those along the southern or western border (Fig. G7a). This arrangement makes it less likely that successful movements will happen between these PAs in Nepal, either now or in a future of potential further habitat loss and fragmentation in the unprotected landscapes, given the lack of protected pathways linking those PAs in the country. Given this situation, a priority for Nepal is the targeted designation of PAs in strategic locations for connectivity (priority A2), as done for example in Bhutan (see section G5), so that these new PAs can function as corridors or stepping stones between the rest of the PAs already designated in the country, thereby contributing to the long-term self-sufficiency of the PA system for connectivity.

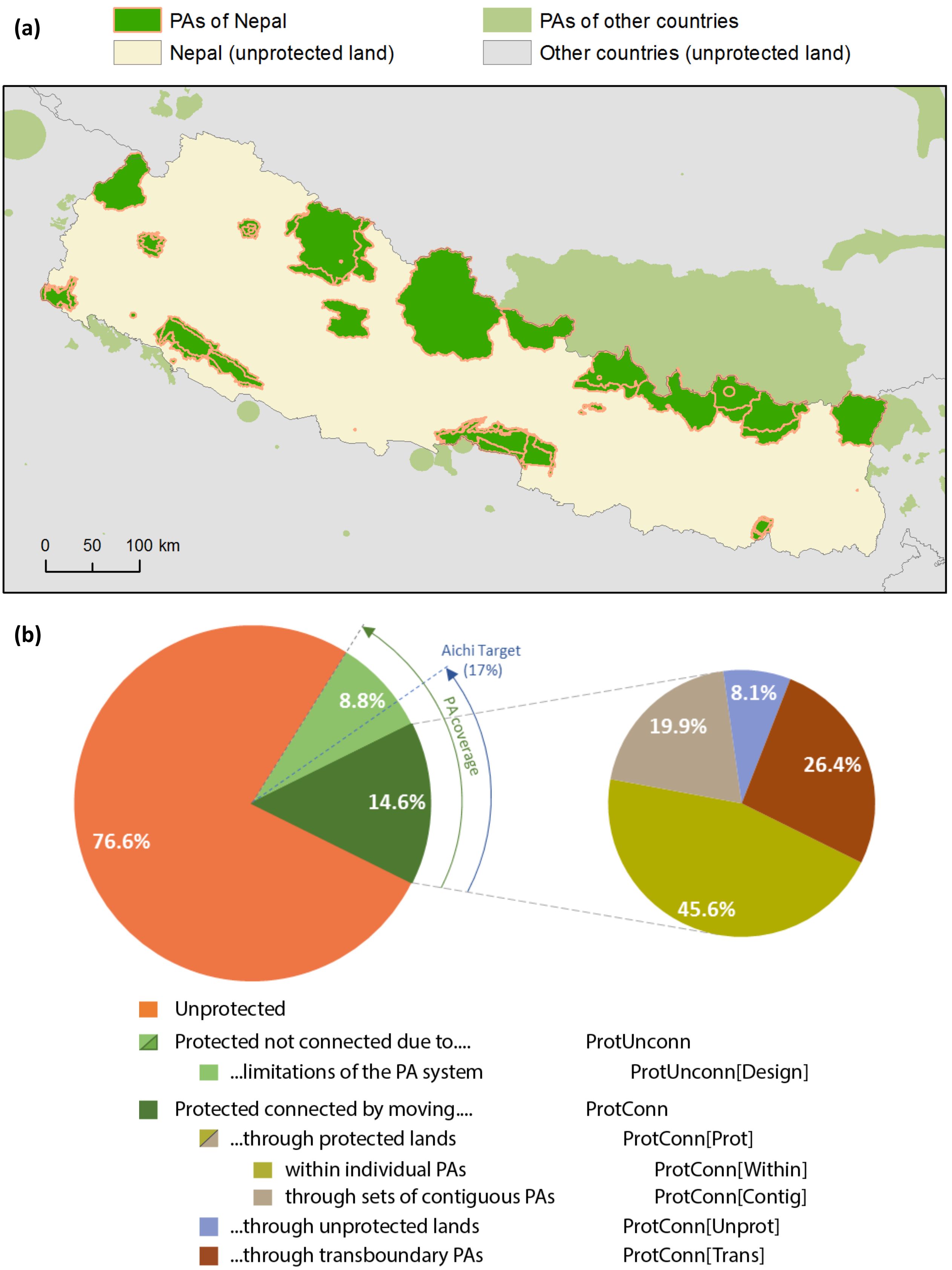

Fig. G7. Protected areas in Nepal and neighbouring countries (a) and ProtConn pie charts for Nepal and d_{med} = 10 km (b). PAs shown in (a) are as given by the WDPA of June 2016. ProtUnconn[Sea] = 0 and ProtUnconn[Outland] = 0 in Nepal, which gives $\text{ProtConn}_{\text{Bound}}$ = ProtConn = 14.6%. PA coverage in Nepal (23.4% = 14.6% + 8.8% = 100% - 76.6%) is above the 17% level of Aichi Target 11, but $\text{ProtConn}_{\text{Bound}}$ still falls below this 17% target.

References cited in Appendix G

Chanchani, P., Lamichhane, B. R., Malla, S., Maurya, K., Bista, A., Warrier, R., Nair, S., Almeida, M., Ravi, R., Sharma, R., Dhakal, M., Yadav, S. P., Thapa, M., Jnawali, S. R., Pradhan, N. M. B., Subedi, N., Thapa, G. J., Yadav, H., Jhala, Y. V., Qureshi, Q., Vattakaven J., Borah, J., 2014. Tigers of the Transboundary Terai Arc Landscape: status, distribution and movement in the Terai of India and Nepal. National Tiger Conservation Authority, Government of India, and Department of National Park and Wildlife Conservation, Government of Nepal.

Chettri, N., Sharma, E., Shakya, B., Bajracharya, B., 2007. Developing forested conservation corridors in the Kangchenjunga Landscape, Eastern Himalaya. Mountain Research and Development 27, 211–214.

Harihar, A., Pandav, B., 2012. Influence of connectivity, wild prey and disturbance on occupancy of tigers in the human-dominated Western Terai Arc Landscape. PLoS ONE 7, e40105.

Küçük, M., Ertürk, E., 2013. Biodiversity and protected areas in Turkey. Sains Malaysiana 42, 1455–1460.

Ministry of Forestry and Water Affairs, 2014. Fifth National Report to the United Nations Convention on Biological Diversity. General Directorate of Nature Conservation and National Parks under Ministry of Forestry and Water Affairs of Republic of Turkey. Available at https://www.cbd.int/doc/world/tr/tr-nr-05-en.pdf

UNEP-WCMC, IUCN, 2016. Protected Planet Report 2016. UNEP-WCMC and IUCN, Cambridge, UK and Gland, Switzerland. The calculated protected area coverage for each country based on WDPA of April 2016, which accompanies this report, is available at https://www.protectedplanet.net/c/protected-planet-report-2016/protected-planet-report-2016--data--maps-figures

Wangchuk, S., 2007. Maintaining ecological resilience by linking protected areas through biological corridors in Bhutan. Tropical Ecology 48, 176-187.
